# Viral Replication, Persistence in Water and Genetic Characterization of Two Influenza A Viruses Isolated from Surface Lake Water

**DOI:** 10.1371/journal.pone.0026566

**Published:** 2011-10-20

**Authors:** Camille Lebarbenchon, My Yang, Shamus P. Keeler, Muthannan A. Ramakrishnan, Justin D. Brown, David E. Stallknecht, Srinand Sreevatsan

**Affiliations:** 1 Southeastern Cooperative Wildlife Disease Study, Department of Population Health, College of Veterinary Medicine, University of Georgia, Athens, Georgia, United States of America; 2 Veterinary Population Medicine, College of Veterinary Medicine, University of Minnesota, St. Paul, Minnesota, United States of America; Duke-NUS Graduate Medical School, Singapore

## Abstract

Water-borne transmission has been suggested as an important transmission mechanism for Influenza A (IA) viruses in wild duck populations; however, relatively few studies have attempted to detect IA viruses from aquatic habitats. Water-isolated viruses have rarely been genetically characterized and evaluation for persistence in water and infectivity in natural hosts has never been documented. In this study, we focused on two IA viruses (H3N8 and H4N6 subtypes) isolated from surface lake water in Minnesota, USA. We investigated the relative prevalence of the two virus subtypes in wild duck populations at the sampling site and their genetic relatedness to IA viruses isolated in wild waterbirds in North America. Viral persistence under different laboratory conditions (temperature and pH) and replication in experimentally infected Mallards (*Anas platyrhynchos*) were also characterized. Both viruses were the most prevalent subtype one year following their isolation in lake water. The viruses persisted in water for an extended time period at constant temperature (several weeks) but infectivity rapidly reduced under multiple freeze-thaw cycles. Furthermore, the two isolates efficiently replicated in Mallards. The complete genome characterization supported that these isolates originated from genetic reassortments with other IA viruses circulating in wild duck populations during the year of sampling. Based on phylogenetic analyses, we couldn't identify genetically similar viruses in duck populations in the years following their isolation from lake water. Our study supports the role for water-borne transmission for IA viruses but also highlights that additional field and experimental studies are required to support inter-annual persistence in aquatic habitats.

## Introduction

Water-borne transmission of influenza A (IA) viruses has been suggested as an important transmission mechanism in wild and domestic duck populations [1], [2]. Experimentally, both low pathogenic (LP) and highly pathogenic (HP) viruses have been shown to persist for extended periods of time in distilled water (several weeks), dependent on temperature, pH and salinity [3]–[6]. Similar results have been reported related to viral infectivity and these same physico-chemical characteristics using field water samples [5], [7]–[9]. In addition, biotic components including aquatic invertebrates (e.g. filter-feeding bivalves) and microorganisms have recently been proposed as potential factors affecting virus removal or accumulation in the environment [10]–[12]. Finally, viral persistence in aquatic habitats has been demonstrated to be a determinant for IA virus transmission dynamics in wild duck populations [13]–[15].

Several studies have attempted to isolate or detect IA viruses from surface water in habitats utilized by waterfowl [16]–[22]. In these studies, virus subtypes detected in local aquatic habitats reflected the current subtype diversity circulating in waterfowl populations. Considering that water-borne transmission drives IA virus dynamics in wild birds [13]–[15], one could expect that a strong selective pressure may exist for IA virus maintenance in aquatic habitats. To date, environmental persistence and replication in the natural host have not been documented for water-isolated viruses, limiting our understanding of viral fitness in ducks and aquatic habitats.

In this study, we performed the complete genome sequencing and estimated the persistence in water and replication in duck of two IA viruses isolated from surface lake water in Minnesota, USA (A/Surface water/Minnesota/NW1-T/2006 H4N6; A/Surface water/Minnesota/W07-2241/2007 H3N8). In particular, we investigated: (i) the relative abundance of the two subtypes in viruses isolated in wild duck populations during three consecutive seasons, at the same sampling site; (ii) the genetic relatedness of these environmental isolates to IA recovered from wild ducks in Minnesota and North America; (iii) the persistence of the two viruses in water under different laboratory-conditions (temperature and pH); and (iv) the ability of these isolates to replicate in experimentally infected Mallards (*Anas platyrhynchos*). We discuss the significance of the results regarding the role of water-borne transmission in IA virus ecology and epidemiology in wild duck populations.

## Results

### Influenza A virus prevalence in duck populations

Based on IA virus isolation, the prevalence of infected waterfowl was 13.1±5.8% and 9.9±1.2% in 2006 and 2007, respectively. The H4N6 subtype represented 5.9±11.2% of the total viruses isolated from ducks in 2006 (1 of 17 isolated viruses, unpublished data), but was the predominant subtype in 2007 (20.7±5.3%; 46 of 222 isolated viruses [23]). Similarly, the H3N8 subtype represented 15.3±4.7% of the total viruses isolated in 2007 (34 of 222 isolated viruses [23]) and was the predominant subtype in 2008 (26±4.1%; 114 of 438 isolated viruses [23]).

### Genetic characterization

The genetic comparisons of the two water isolates showed variable percentages of similarity for the internal segments: PB2: 91%; PB1: 91%; PA: 87%; NP: 95%; M: 98%; NS: 71%. Results from the phylogenetic analysis indicate that, except for the Matrix gene, these genes belonged to different genetic lineages (Figures S1, S2, S3, S4, S5, S6). When considering the internal segments, both viruses showed high genetic relatedness with a large diversity of IA virus subtypes circulating in wild waterbirds in Minnesota during the same season. In particular, A/Surface water/Minnesota/W07-2241/2007 (H3N8) was closely related to different virus subtypes (H1N3, H3N8, H4N6, H6N6, H10N1, H10N3, H10N6, H11N2, H11N9) for PB1, PA and M genes and A/Surface water/Minnesota/NW1-T/2006 (H4N6) was genetically related to A/Mallard/MN/464334/2006 (H5N2) for PB2 and NP genes (99% and 96% similarity, respectively).

**Figure 1:**
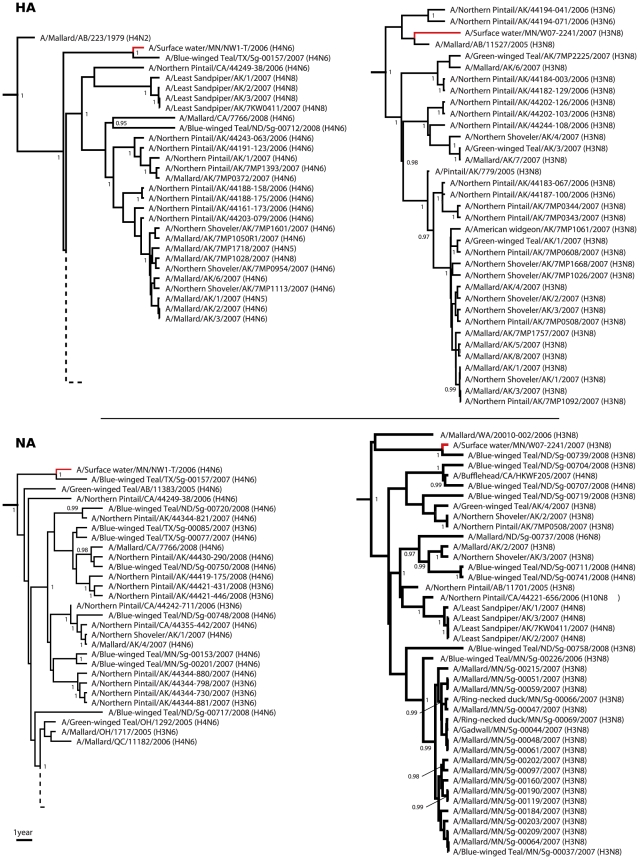
Genetic sub-lineages including the two water-isolated viruses, for the HA and NA genes. Posterior probability values are indicated when higher than 0.95. Complete maximum clade credibility trees are available in Figures S7, S8, S9 and S10.

Analysis of the HA and NA segments provided additional information concerning the circulation of these two subtypes in wild waterbirds in North America (Figures 1 and S7, S8, S9, S10). A/Surface water/Minnesota/NW1-T/2006 (H4N6) was closely related to A/Blue-winged Teal/TX/Sg-00157/2007 (H4N6) for both genes. For A/Surface water/Minnesota/W07-2241/2007 (H3N8), the HA was closely related to H3N6 and H3N8 viruses isolated in Alaska and Alberta the previous years (2005 and 2006) and, for the NA, to a virus isolated in 2008 in North-Dakota: A/Blue-winged Teal/ND/Sg-00739/2008 (H3N8).

### Persistence under laboratory conditions

Results of viral persistence trials are presented in Table 1 (adjusted R^2^ and P values are detailed in Table S1). Overall, increasing temperature decreased virus persistence. A slight decrease of viral titer was observed at 4°C; both viruses were detected 365 days post-inoculation. Infectivity was quickly reduced when exposed to repeated freeze-thaw cycles (−20°C/4°C); however, viruses remained viable when the temperature was changed between 17°C and 23°C, consistent with the results of maintenance at constant temperatures (Table 1).

**Table 1:** Estimated time (in days) required for a decrease of the viral titer by 1 log_10_ TCID_50_/mL.

Virus	pH	Constant temperature					Variable temperature	
		4°C	10°C	17°C	23°C	28°C	−20°C/4°C	17°C/23°C
	6.2			13		6		
A/Surface water/MN/W07-2241/2007 (H3N8)	7.2	211	175	79	51	11	6	82
	8.2			22		8		
	6.2			9		3		
A/Surface water/MN/NW1-T/2006 (H4N6)	7.2	270	193	102	51	13	11	62
	8.2			18		7		

### Replication in Mallards

None of the birds were shedding virus at the time of inoculation and all tested negative for IA virus antibodies. Both viruses were isolated from all ducks from day 1 to day 5 post-inoculation and intermittently from day 7 to 14 post-inoculation (Table 2). Viruses were isolated from both oropharyngeal and cloacal swabs. Real-time RT-PCR results were consistent with viral isolation results for the first five days post-inoculations, except for day 1 for A/Surface water/Minnesota/W07-2241/2007 (H3N8) and day 5 for one oropharyngeal swab for A/Surface water/Minnesota/NW1-T/2006 (H4N6).

**Table 2:** Virus shedding pattern based on oropharyngeal (OP) and cloacal (CL) swab samples.

Virus	Sample	Days post-inoculation								
		1	2	3	4	5	7	9	11	14
A/Surface water/MN/W07-2241/2007 (H3N8)	OP	5 (2)	5 (5)	5 (5)	5 (5)	5 (5)	1 (0)	1 (0)	2 (0)	1 (0)
	CL	5 (1)	5 (5)	5 (5)	5 (5)	5 (5)	3 (2)	3 (2)	2 (1)	0 (0)
A/Surface water/MN/NW1-T/2006 (H4N6)	OP	5 (5)	5 (5)	5 (5)	5 (5)	5 (4)	2 (1)	3 (0)	4 (0)	2 (0)
	CL	5 (5)	5 (5)	5 (5)	5 (5)	5 (5)	2 (5)	2 (3)	5 (1)	2 (0)

Number of ducks tested positive for viral isolation and real-time RT-PCR (in parenthesis).

For A/Surface water/Minnesota/W07-2241/2007 (H3N8), four of the five ducks were ELISA positive at day 4 post-inoculation and all ducks were positive at days 7, 10 and 14. All ducks inoculated with A/Surface water/Minnesota/NW1-T/2006 (H4N6) tested positive for IA antibody from day 4 until the end of the experiment (day 14).

## Discussion

Both the H3N8 and H4N6 subtypes that were isolated from surface lake water have been commonly detected in North American waterfowl [24]. At our study sites, the prevalence of these subtypes was relatively low in wild duck populations during the years of detection; however, the limited number of viruses recovered during 2006 provided limited information on virus subtypes circulating in local duck populations during that year. Interestingly, both the H3 and H4 virus subtypes were the most prevalent the year following their isolation in lake water [23]. Although circumstantial, this pattern is consistent with a possible inter-annual environmental persistence.

Phylogenetic analyses suggested that both isolates were reassortants related to viruses circulating in wild ducks in Minnesota at the time of sampling. In addition, we provide evidence that surface glycoproteins were highly similar to others identified in viruses circulating in North American ducks both before and after their detection in Minnesota. Based on phylogenetic analyses, no similar viral strain (i.e. when considering all gene segments) were however identified the years following their isolation in lake water, in Minnesota. This result suggests that, for the two water isolates, local persistence in aquatic habitats over winter was unlikely; although we recognize that this conclusion is based on sequences from limited number of viruses that may not fully represent the diversity of IA virus circulating in Minnesota duck populations [23]. Genetic characterization of viruses isolated in the environment can provide interesting information regarding gene prevalence and circulation in habitats used by waterfowl. Such information is critical to assess the possibility of inter-annual persistence in water, long-term circulation of environmentally-isolated virus strains in wild duck populations, and identify potential genetic basis for the persistence of IA viruses in water.

Previous studies have demonstrated differences in the ability of IA viruses to remain infective in water and have suggested that subtype-related variations in persistence may exist, especially at low temperatures [3], [4]. Both isolates tested in our study persisted for several months in distilled water at constant temperatures, below 17°C and at neutral pH. Overall, the estimated persistence was higher than reported for 12 different waterfowl derived viruses (including a H3 and H4 subtypes; [3]). Limited data precludes conclusions related to increased environmental persistence associated with viruses recovered from the environment. Future experiments should however consider such a possibility as the demonstration of long-time persistence of water-isolated viruses would suggest the potential for inter-annual persistence in aquatic habitats.

Freeze-thaw cycles have been documented to significantly reduce virus infectivity [18]. As expected, cycles of temperature changes between −20°C and 4°C reduced viral persistence (6–11 days) as compared to a constant temperature of 4°C (211–270 days). We propose that freeze-thaw cycles could be limiting for the persistence of IA viruses in aquatic habitats especially before and after winter, when surface water is subjected to diurnal temperature changes around 0°C. Dabbling ducks utilize shallow water for feeding that are likely to undergo important temperature changes and freeze-thaw cycles during winter, potentially precluding long-time persistence of IA viruses. Inversely, habitats characterized by deep water may prevent freezing and strong temperature changes affecting viral persistence, but are less likely to be used by dabbling ducks. Viral persistence in frozen pond and lake water has been suggested to be an potential mechanisms for inter-annual persistence and source of contamination for waterfowl in breeding grounds [16], [17]. In high latitude habitats, shallow lakes and ponds may freeze relatively quickly during fall and winter, potentially reducing effects of such temperature changes. The effects of latitudinal-dependent temperature regimes on surface water viruses warrant future experimental work.

Because diurnal surface water temperature fluctuations could represent a constraint on viral persistence, we also investigated the effects of temperature variations between 17°C and 23°C. Compared to results from constant temperature, no effects on viral persistence were observed. This suggests that diurnal surface water temperature fluctuations are not likely to significantly affect virus persistence, although as for freeze-thaw cycles additional environmental simulation studies would be required to confirm this trend.

Experimental infections provided evidence that both water-isolated IA viruses replicated efficiently in Mallards and, in both cases, shedding patterns were consistent with a previous study performed with duck-isolated LP IA viruses [25]. These results support that viruses isolated from surface water replicate in ducks as well as LP virus isolates that have a wild duck-origin.

In this study, we highlighted that water-isolated IA viruses can provide complementary information regarding circulating viruses and viral genes in wild waterfowl populations. With current methodological limitations the recovery of viruses from aquatic habitats should not be considered as a replacement of traditional surveillance approaches utilizing wild bird or fecal sampling. We also demonstrated that both viruses could persist for extended periods of time in water (an attribute that might explain their original isolation) and could efficiently replicate in Mallards. However, data on freeze-thaw cycles presented herein could preclude long-term persistence in habitats that freeze during winter, suggesting that the environmental reservoir may not be surface water but other components of aquatic habitats. Our study supports the role for water-borne transmission for IA viruses; however, it also raises questions regarding inter-annual persistence in aquatic habitats.

## Materials and Methods

### Viruses

Both viruses were isolated from surface lake water in Minnesota, USA: A/Surface water/Minnesota/NW1-T/2006 H4N6 was isolated from a water sample collected in Thief Lake Wildlife Management Area (48°30′40″N, 95°55′12″W), in September 18, 2006; and A/Surface water/Minnesota/W07-2241/2007 H3N8 from a sample collected in Agassiz National Wildlife Refuge (48°18′58″N, 96°00′23″W), in October 28, 2007. Information related to water sample collection and virus isolation have been previously described [18]. Second (H4N6) or third (H3N8) passages of stock viruses were propagated in 9 to 11 days old specific pathogen free (SPF) embryonating chicken eggs. Amnio-allantoic fluids were harvested 5 days post infection and were titrated in both 9 to 11 days old SPF embryonating chicken eggs and Madin Darby canine kidney (MDCK) cells [3]; titers were determined according to the method described by Reed and Muench [26].

### Influenza A virus prevalence in duck populations

Prevalence estimates and subtype diversity were available from ongoing long-term population studies of wild duck populations in Northwestern Minnesota [23]. In total, 130 and 2246 cloacal samples were collected from wild ducks in 2006 and 2007, respectively. Sampling was performed in September in 2006 and from July to October in 2007. Detailed method for sample collection, virus isolation and subtyping were previously described [23]. In brief, cloacal swabs were obtained using sterile cotton-tipped applicators (Puritan, Medical Products Company LLC, Guilford, ME) and placed in 2 ml of Brain Heart Infusion media (Becton Dickinson and Co., Sparks, MD) supplemented with penicillin G (1,000 units/ml), streptomycin (1 mg/ml), kanamycin (0.5 mg/ml), gentamicin (0.25 mg/ml), and amphotericin B (0.025 mg/ml) (Sigma Chemical Company, St. Louis, MO). Samples were stored at 4°C (24–72 hrs) and then frozen at −80°C until processed. Each sample was inoculated into four 9–11 day old SPF embryonated chicken eggs via the allantoic route (0.25 ml/egg). Amnio allantoic fluid were tested by hemagglutination assay and positive samples were confirmed by RT-PCR [27]. Subtyping was done at the National Veterinary Services Laboratories (Ames, Iowa) using hemagglutination inhibition and neuraminidase inhibition tests and via genotyping of the HA and NA genes, at the University of Minnesota, St. Paul, Minnesota and the University of California Davis, using standard Sanger sequencing for HA and NA segments.

For virus and subtype prevalences, 95% confidence intervals (CI) were calculated with the equation for the normal approximation for the binomial CI (CI = 1.96√(p(1−p)/n; where p is the observed prevalence and n is the sample size).

### Full-genome sequencing

Sample preparation, viral RNA extraction, and amplification of all segments of the genome in a single step multiplex PCR were performed as described by Zhou et al. [28] with a modification of single base in primer MBTuni-12 (the new primer is MBTuni-12(M): 5′- ACGCGTGATCAGCRAAAGCAGG-3′). cDNA libraries were prepared from PCR-purified multiplex amplicons using QIAquick PCR Purification kit (Qiagen, Valencia, California USA), following manufacturer's instructions, and subjected to adaptor ligation and whole genome sequencing using 454 FLX technology using protocols described by Ramakrishnan et al. [29]. Obtained DNA sequences were analyzed as previously described [29]. In brief, all the sequencing reads were aligned against influenza genomes using BlastN algorithm of NCBI BLAST 2.2.16. The ‘non influenza’ sequences were filtered out and only influenza reads were assembled in GS De nova Assembler 2.0.00.20 and mapped in GS Reference Mapper 2.0.00.20. The influenza contigs obtained using the above software were reassembled in Sequencher Version 4.9 (Genecodes) and annotated based on BLAST analyses.

Sequences generated in this study have been deposited in GenBank under the accession numbers CY073707 to CY073714 (A/Surface water/Minnesota/NW1-T/2006) and CY073717 to CY073724 (A/Surface water/Minnesota/W07-2241/2007).

### Phylogenetic analysis

Complete nucleotide sequences of the 8 segments of IA viruses isolated in wild waterbirds in North America were retrieved from the Influenza Sequence Database [30]. For computational reasons, we selected only sequences obtained from viruses isolated in Minnesota for internal segments (PB2, PB1, PA, NP, MP and NS). For the HA and NA segments, the dataset was extended to viruses isolated at other sites in North America. For each sequence, the following information was collected: subtype, geographic origin (country and state), strain name, bird host species and year of collection. Analysis was restricted to viruses isolated from wild avian species belonging to the Anseriformes and Charadriiformes orders, as they are recognized to be the natural host reservoir for IA viruses. Sequences for which bird species were not identified (e.g., ‘duck’ or ‘bird’, without any additional information concerning their origin) were not included. Duplicate sequences from the same strains, undefined subtypes and mixed infections, as well as sequences previously identified as reflecting potential laboratory errors [31], also were not included. Sequences were aligned with CLC sequence viewer 6.3 (CLC bio, Aarhus, Denmark) and edited in order to restrict the alignment to the coding region for each gene. The definitive data sets included 2121 nucleotide sequences of viruses isolated between 1976 and 2009 (c.f. Table S2 and S3 for details).

Phylogenetic trees were generated with a Bayesian Markov chain Monte Carlo (MCMC) coalescent approach, implemented in the program BEAST 1.5.3 [32]. The Shapiro-Rambaut-Drummond-2006 (SRD06) nucleotide substitution model was used in all simulations as this model is recognized to provide better resolution for coding regions [33]. Three molecular clock models were tested for each gene: the strict clock (SC) that assumes a single evolutionary rate in the phylogenetic trees and two relaxed clocks: the uncorrelated exponential (UE) and the uncorrelated lognormal (UL) that allows evolutionary rates to vary along branches, within an exponential or lognormal distribution [34]. Molecular clock models were evaluated and tested with the Bayes Factor (BF) [35], [36] implemented in the program TRACER 1.5.0 [37]. The ratio of marginal likelihoods were compared between models and BF significance was determined from the values of 2 ln(BF) as described in Brandley et al. [38]. A Bayesian skyline coalescent tree prior was used in all simulations as it makes the fewest a priori assumptions about the data [39] and has been shown to be more appropriate to describe the population dynamics of IA virus [40]. Three independent analysis were performed (with the SC, UE and UL molecular clocks) for each segment, with a chain length of 140–320 million generations sampled every 1000 iterations. Results were analyzed with TRACER 1.5.0, phylogenetic consensus trees were produced using the program TREEANNOTATOR 1.5.3 and edited for generation of figure captions with the program FigTree 1.3.1.

### Persistence in distilled water

Distilled water buffered with 10 mM HEPES was adjusted with 1 N solutions of NaOH or HCl to provide 3 different pH conditions (6.2, 7.2 and 8.2). Five constant temperatures (4°C, 10°C, 17°C, 23°C and 28°C) were tested at pH = 7.2. Two additional pH conditions (6.2, 8.2) were tested at 17°C and 28°C. For both viruses, infective amnio-allantoic fluids were diluted 1∶100 in the water samples. Inoculated water samples were divided into 2 mL aliquots in 5 mL polystyrene round-bottom tubes and placed in incubators set to the appropriate temperature. The effect of temperature changes was also investigated at two conditions (pH = 7.2): −20°C/4°C and 17°C/23°C. Inoculated water samples were transferred daily into the appropriate incubators in order to reflect cyclic temperature changes: samples were maintained 14 hours under low temperatures (−20°C and 17°C) and 10 hours under high temperature (4°C and 23°C).

For each treatment, the viral-inoculated water was sampled at the time of inoculation (day 0) and between 9 to 23 times post-inoculation. Sampling frequency varied between treatments (from 1 to 28 days) and was adjusted during the course of the experiment to ensure at least a 1 log_10_ decrease of the TCID_50_/mL. Experiments were conducted during 10 to 365 days post-inoculation, depending on the treatment, with a final sampling performed the last day of the experiment. At the time of sampling, duplicate 0.5 mL samples of IA virus-inoculated water were diluted 1∶1 by addition of 0.5 mL of 2× MEM. Ten-fold dilutions (10^−1^ to 10^−6^) were made in 1× MEM supplemented with antibiotics (10000 U Penicillin G, 10 mg Streptomycin, 25 µg Amphotericin B/mL).

Infectivity assays were performed on MDCK, following the method described by Brown et al. [3]. Results from duplicate titrations were log_10_ transformed and averaged. Simple linear regressions were used to calculate the time required for a 90% reduction in infectivity (i.e. time required for a decrease of the viral titer by 1 log_10_ TCID_50_/mL); previous studies indicated that loss of IA virus infectivity in water over time decreased at a log-linear rate [3], [5]. Statistical analysis was performed in R 2.10.1 [41].

### Experimental infections

One-day-old Mallards were purchased from a commercial source (Murray McMurray Hatchery, Webster City, Iowa, USA) and raised for one month under confined conditions until the beginning of the experiments. Three days before inoculation, ducks were separated into two groups of five and transferred to self-contained isolation units ventilated under negative pressure with high-efficiency particulate air-filters. Each duck was weighed before inoculation and samples were collected to test for IA virus shedding (oropharyngeal and cloacal swabs) or antibodies (serum). General care was provided in accordance to an animal use protocol (AUP # A2010 6-101) approved by the Institutional Animal Care and Use Committee at the University of Georgia, Athens, Georgia, USA. All work was conducted at an enhanced Biosafety Level 2 facility at the Poultry Diagnostic Research Center, Athens, Georgia, USA.

Five birds in each group were inoculated in the choanal cleft and the trachea with a volume of 0.2 mL containing an infectious titer of 10^5.63^ EID_50_ for A/Surface water/Minnesota/NW1-T/2006 and 10^6.54^ EID_50_ for A/Surface water/Minnesota/W07-2241/2007 (based on back titers). Oropharyngeal and cloacal swabs were collected from all birds on days 1, 2, 3, 4, 5, 7, 9, 11 and 14 post-inoculation and stored at −80°C until viral isolation and RNA extractions were performed. Blood samples were collected from the right jugular vein on days 4, 7, 10 and 14 post-inoculation, centrifuged for 30 minutes at 1500 rpm, and sera stored at −20°C until testing. Birds were evaluated twice daily and were weighed on days 4, 7, 10 and 14 post-inoculation to ensure that they gained weight during the course of the experiment.

Viral isolation was performed on cloacal and oropharyngeal swabs using 9 to 11 days old SPF embryonating chicken eggs following previously described procedures [42]. For each sample, RNA was extracted the same day as viral isolation was performed, with the MagMAX™-96 AI/ND Viral RNA Isolation kit (Ambion, Austin, Texas, USA) using the Thermo Electron KingFisher magnetic particle processor (Thermo Electron Corporation, Waltham, Massachusetts, USA) according to the modified protocol proposed by Das et al. [43]. Real-time RT-PCR targeting the Matrix gene was conducted with the QIAGEN OneStep RT-PCR kit (QIAGEN Valencia, California, USA) and the Cepheid SmartCycler System (Cepheid, Sunnyvale, California, USA) following the protocol described by Spackman et al. [44]. Samples with a cycle threshold value equal or less than 40 were considered positive.

Serum samples were tested with a commercial ELISA assay (IDEXX FlockCheck IA MultiS-Screen Antibody Test Kit, IDEXX Laboratories, Westbrook, Maine, USA) according to the manufacturer's instructions.

## Supporting Information

Figure S1 **Maximum clade credibility tree for PB2 of viruses isolated in wild waterbirds in Minnesota, between 1979 and 2008.** Red dots represent nodes with posterior probability values superior to 0.95. Viruses characterized in this study are colored in blue. Viral strain names and sequence accession numbers are listed in Table S3.
(PDF)

Figure S2 **Maximum clade credibility tree for PB1 of viruses isolated in wild waterbirds in Minnesota, between 1979 and 2008.** Red dots represent nodes with posterior probability values superior to 0.95. Viruses characterized in this study are colored in blue. Viral strain names and sequence accession numbers are listed in Table S3.
(PDF)

Figure S3 **Maximum clade credibility tree for PA of viruses isolated in wild waterbirds in Minnesota, between 1979 and 2008.** Red dots represent nodes with posterior probability values superior to 0.95. Viruses characterized in this study are colored in blue. Viral strain names and sequence accession numbers are listed in Table S3.
(PDF)

Figure S4 **Maximum clade credibility tree for NP of viruses isolated in wild waterbirds in Minnesota, between 1979 and 2007.** Red dots represent nodes with posterior probability values superior to 0.95. Viruses characterized in this study are colored in blue. Viral strain names and sequence accession numbers are listed in Table S3.
(PDF)

Figure S5 **Maximum clade credibility tree for M (M1) of viruses isolated in wild waterbirds in Minnesota, between 1979 and 2008.** Red dots represent nodes with posterior probability values superior to 0.95. Viruses characterized in this study are colored in blue. Viral strain names and sequence accession numbers are listed in Table S3.
(PDF)

Figure S6 **Maximum clade credibility tree for NS of viruses isolated in wild waterbirds in Minnesota, between 1979 and 2007.** Red dots represent nodes with posterior probability values superior to 0.95. Viruses characterized in this study are colored in blue. Viral strain names and sequence accession numbers are listed in Table S3.
(PDF)

Figure S7 **Maximum clade credibility tree for HA (H3) of viruses isolated in wild waterbirds in North America, between 1976 and 2008.** Red dots represent nodes with posterior probability values superior to 0.95. The virus characterized in this study is colored in blue and the box represents the genetic sub-lineage detailed in Figure 1. Viral strain names and sequence accession numbers are listed in Table S3.
(PDF)

Figure S8 **Maximum clade credibility tree for HA (H4) of viruses isolated in wild waterbirds in North America, between 1977 and 2008.** Red dots represent nodes with posterior probability values superior to 0.95. The virus characterized in this study is colored in blue and the box represents the genetic sub-lineage detailed in Figure 1. Viral strain names and sequence accession numbers are listed in Table S3.
(PDF)

Figure S9 **Maximum clade credibility tree for NA (N6) of viruses isolated in wild waterbirds in North America, between 1976 and 2009.** Red dots represent nodes with posterior probability values superior to 0.95. The virus characterized in this study is colored in blue and the box represents the genetic sub-lineage detailed in Figure 1. Viral strain names and sequence accession numbers are listed in Table S3.
(PDF)

Figure S10 **Maximum clade credibility tree for NA (N8) of viruses isolated in wild waterbirds in North America, between 1979 and 2007.** Red dots represent nodes with posterior probability values superior to 0.95. The virus characterized in this study is colored in blue and the box represents the genetic sub-lineage detailed in Figure 1. Viral strain names and sequence accession numbers are listed in Table S3.
(PDF)

Table S1 **Adjusted R^2^ and P value obtained for linear regression models.**
(PDF)

Table S2 **Information related to nucleotide sequences used for phylogenetic analysis.**
(PDF)

Table S3 **Viral strain names, accession numbers and taxa codes used in Figures S1, S2, S3, S4, S5, S6, S7, S8, S9, S10.**
(PDF)
