# Supplementary figures and images for: Viral Replication, Persistence in Water and Genetic Characterization of Two Influenza A Viruses Isolated from Surface Lake Water
Source: PLoS One. 2011 Oct 20;6(10):e26566. doi: 10.1371/journal.pone.0026566 (PMC3197669; doi:10.1371/journal.pone.0026566)

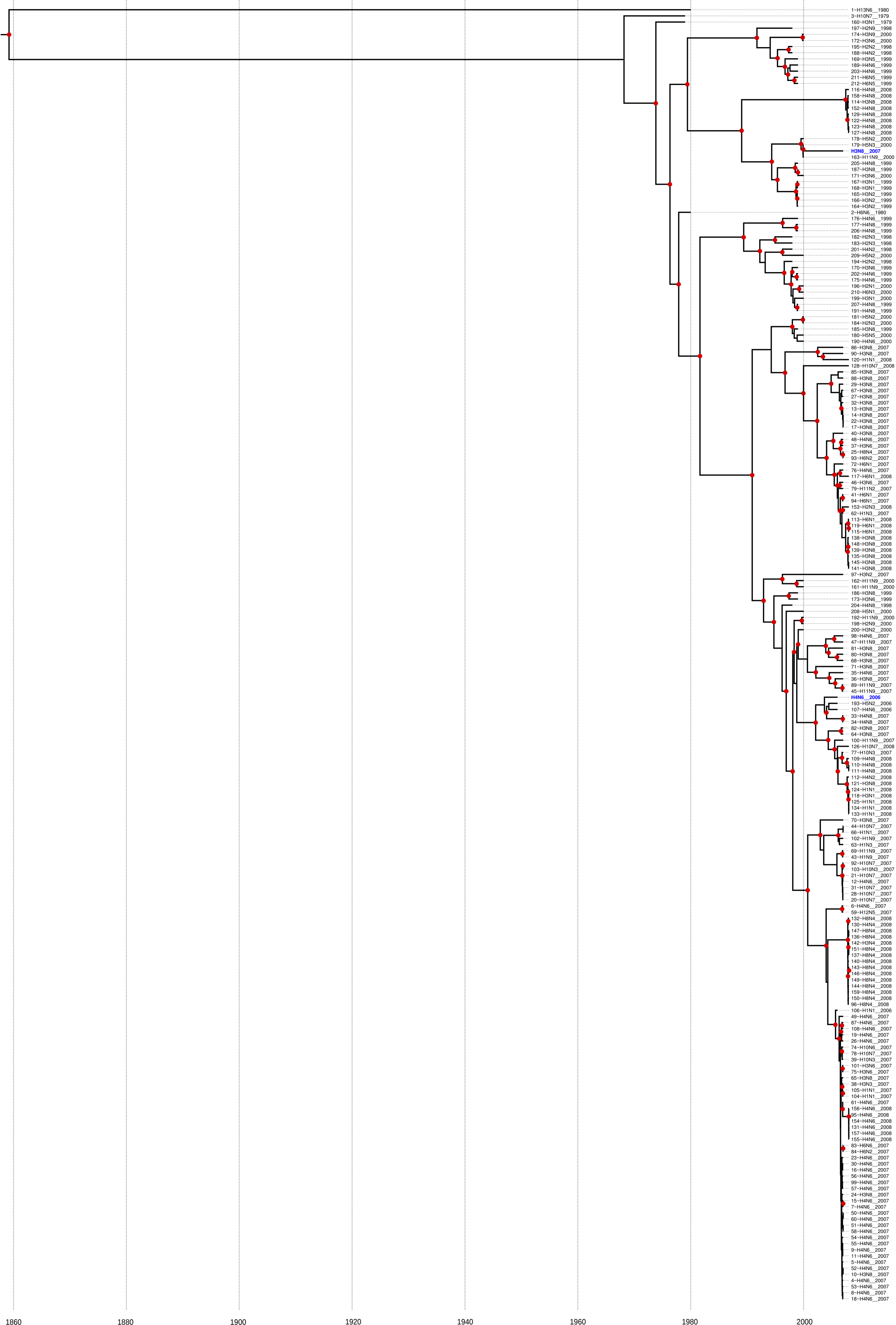

Supplement: Figure S1 — Maximum clade credibility tree for PB2 of viruses isolated in wild waterbirds in Minnesota, between 1979 and 2008. Red dots represent nodes with posterior probability values superior to 0.95. Viruses characterized in this study are colored in blue. Viral strain names and sequence accession numbers are listed in Table S3. (PDF) [file pone.0026566.s001.pdf]

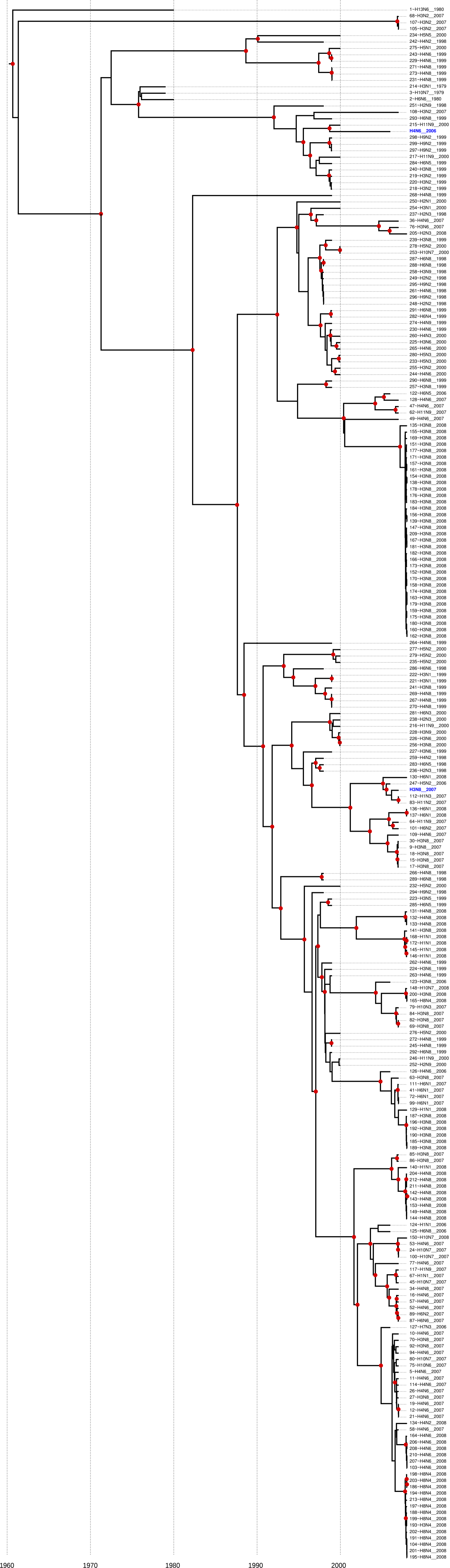

Supplement: Figure S2 — Maximum clade credibility tree for PB1 of viruses isolated in wild waterbirds in Minnesota, between 1979 and 2008. Red dots represent nodes with posterior probability values superior to 0.95. Viruses characterized in this study are colored in blue. Viral strain names and sequence accession numbers are listed in Table S3. (PDF) [file pone.0026566.s002.pdf]

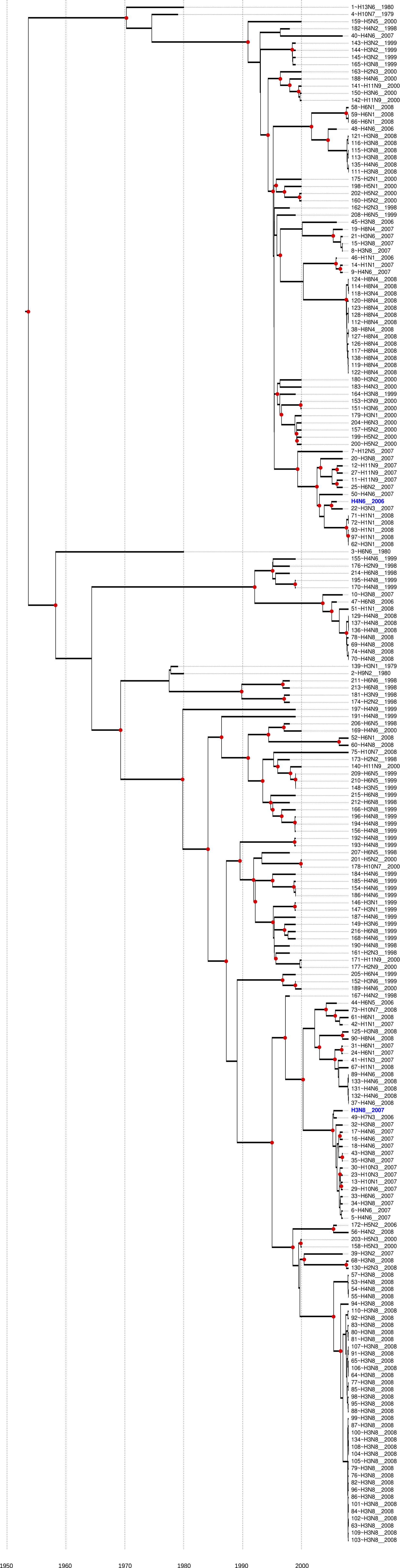

Supplement: Figure S3 — Maximum clade credibility tree for PA of viruses isolated in wild waterbirds in Minnesota, between 1979 and 2008. Red dots represent nodes with posterior probability values superior to 0.95. Viruses characterized in this study are colored in blue. Viral strain names and sequence accession numbers are listed in Table S3. (PDF) [file pone.0026566.s003.pdf]

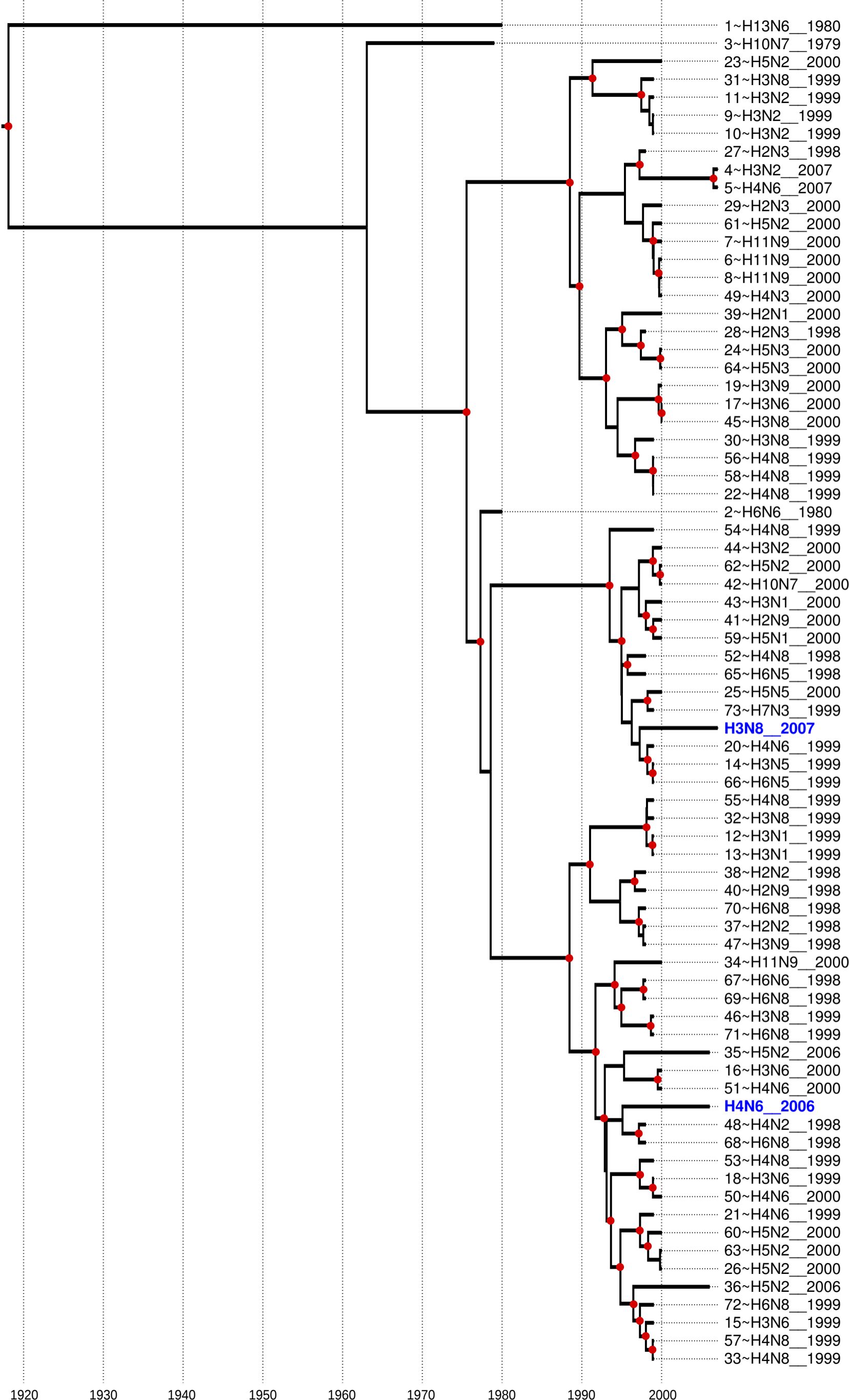

Supplement: Figure S4 — Maximum clade credibility tree for NP of viruses isolated in wild waterbirds in Minnesota, between 1979 and 2007. Red dots represent nodes with posterior probability values superior to 0.95. Viruses characterized in this study are colored in blue. Viral strain names and sequence accession numbers are listed in Table S3. (PDF) [file pone.0026566.s004.pdf]

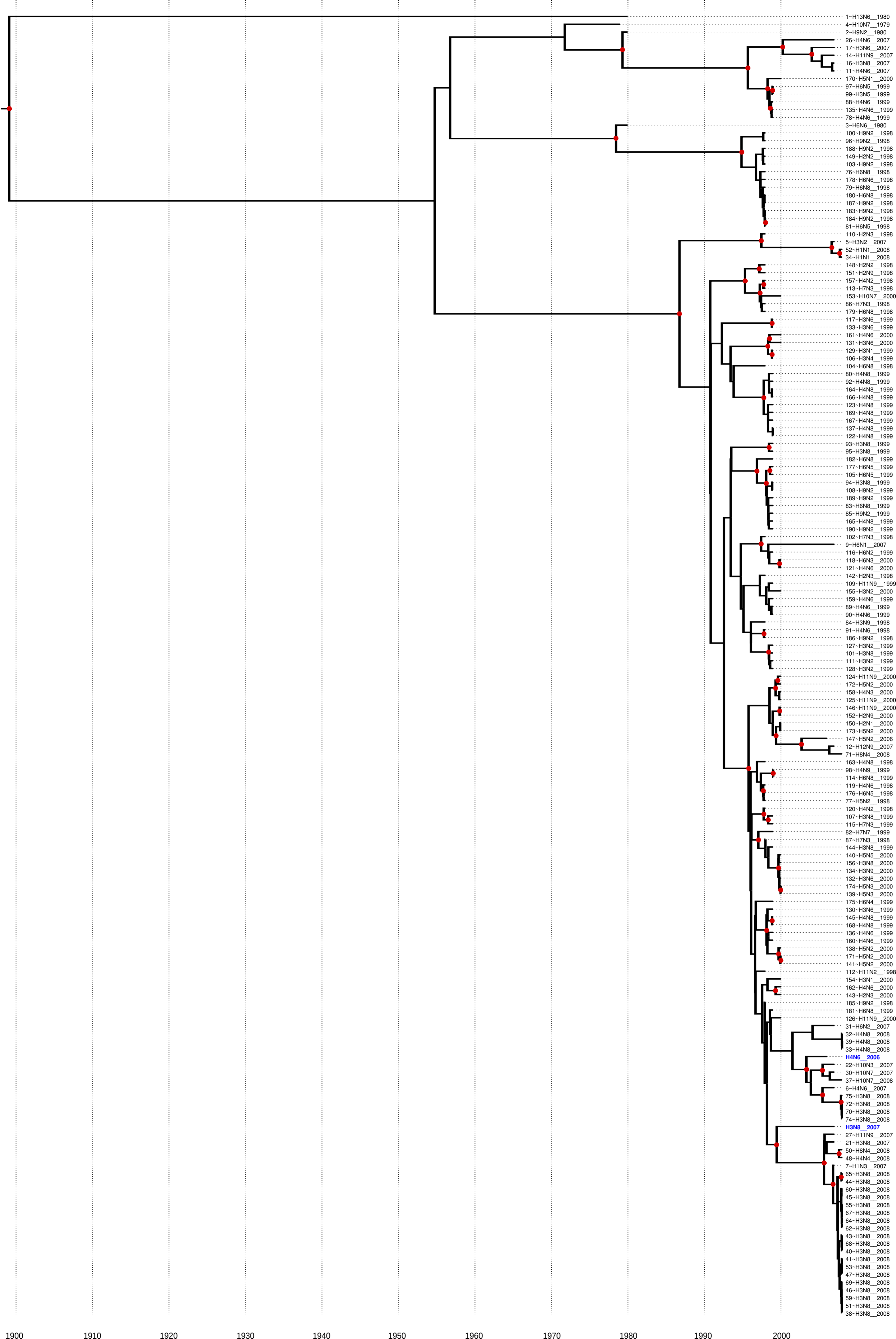

Supplement: Figure S5 — Maximum clade credibility tree for M (M1) of viruses isolated in wild waterbirds in Minnesota, between 1979 and 2008. Red dots represent nodes with posterior probability values superior to 0.95. Viruses characterized in this study are colored in blue. Viral strain names and sequence accession numbers are listed in Table S3. (PDF) [file pone.0026566.s005.pdf]

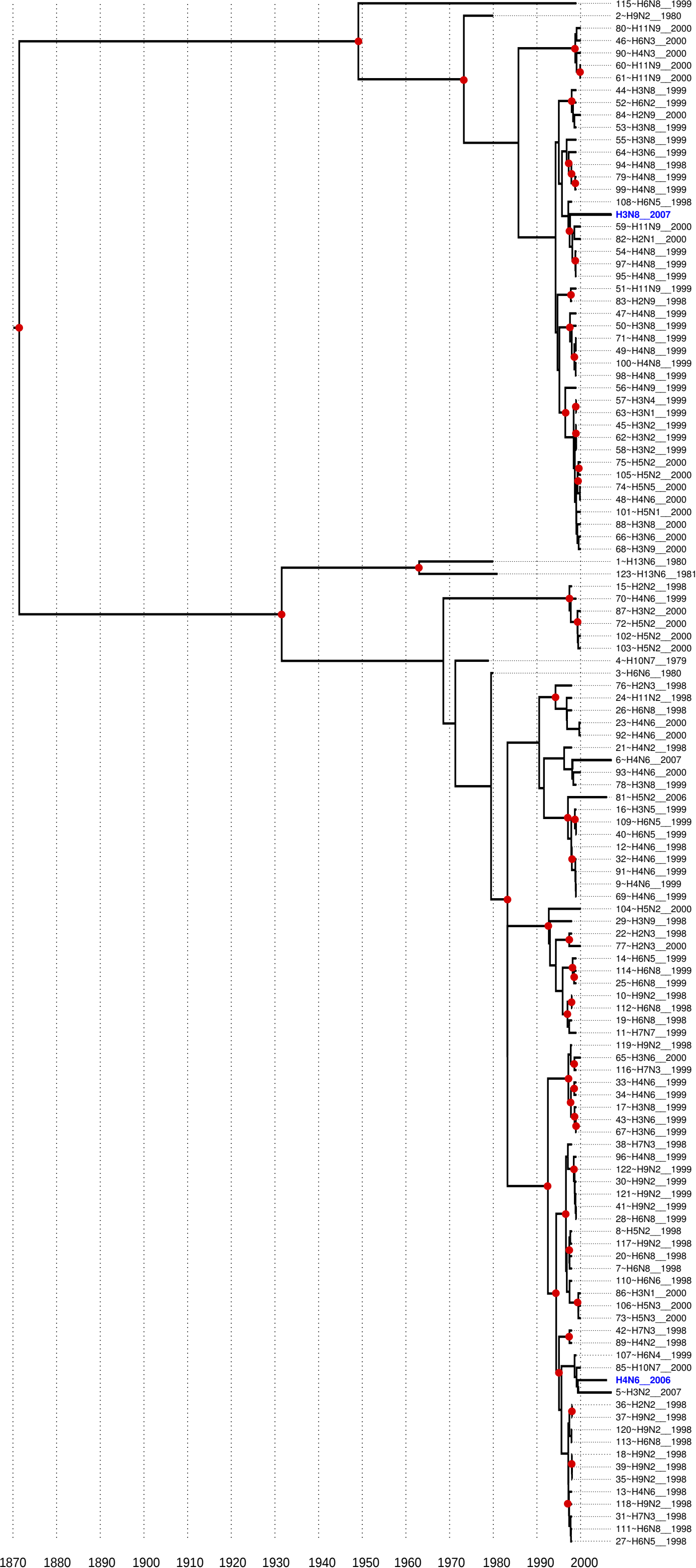

Supplement: Figure S6 — Maximum clade credibility tree for NS of viruses isolated in wild waterbirds in Minnesota, between 1979 and 2007. Red dots represent nodes with posterior probability values superior to 0.95. Viruses characterized in this study are colored in blue. Viral strain names and sequence accession numbers are listed in Table S3. (PDF) [file pone.0026566.s006.pdf]

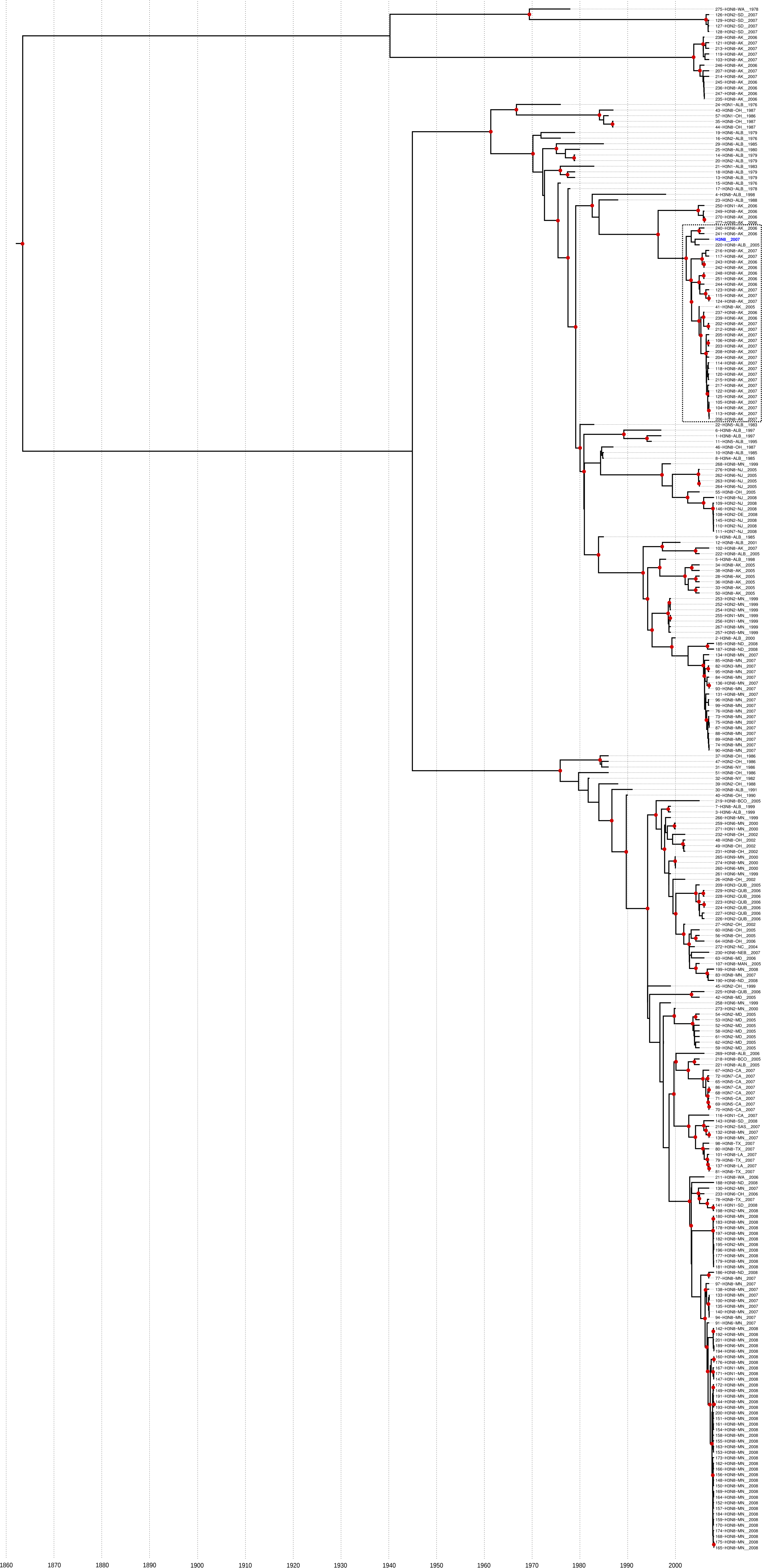

Supplement: Figure S7 — Maximum clade credibility tree for HA (H3) of viruses isolated in wild waterbirds in North America, between 1976 and 2008. Red dots represent nodes with posterior probability values superior to 0.95. The virus characterized in this study is colored in blue and the box represents the genetic sub-lineage detailed in Figure 1. Viral strain names and sequence accession numbers are listed in Table S3. (PDF) [file pone.0026566.s007.pdf]

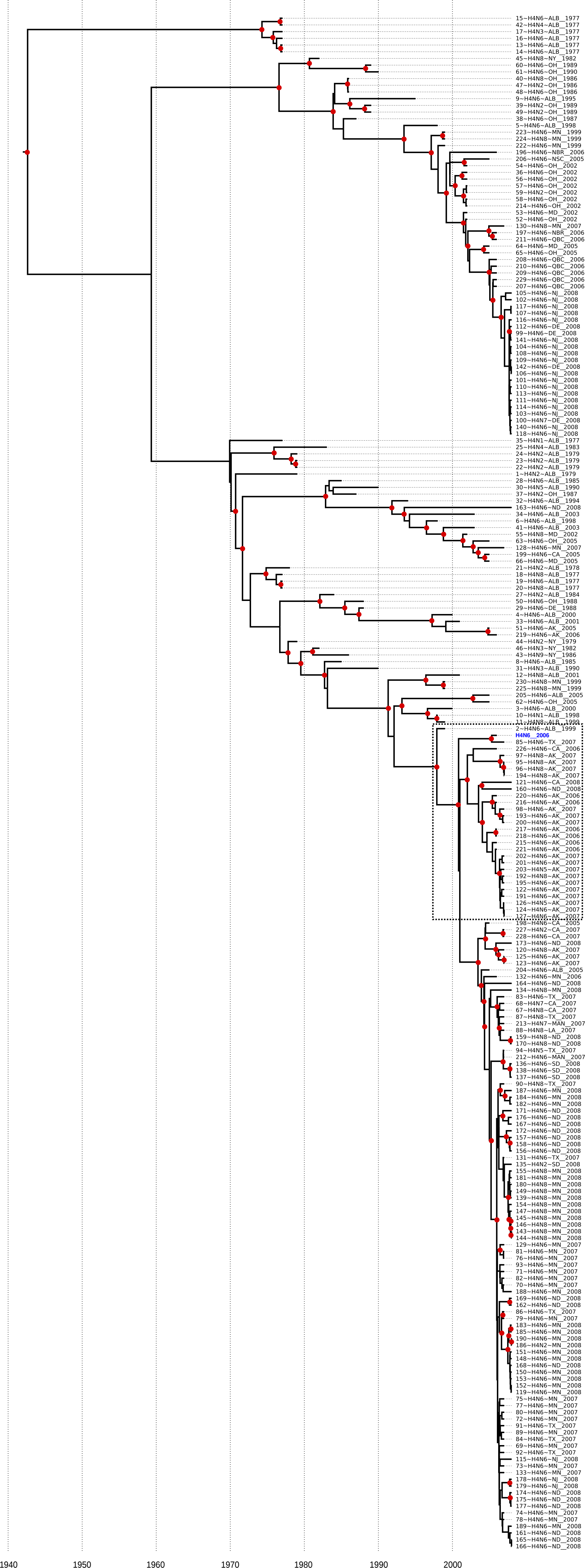

Supplement: Figure S8 — Maximum clade credibility tree for HA (H4) of viruses isolated in wild waterbirds in North America, between 1977 and 2008. Red dots represent nodes with posterior probability values superior to 0.95. The virus characterized in this study is colored in blue and the box represents the genetic sub-lineage detailed in Figure 1. Viral strain names and sequence accession numbers are listed in Table S3. (PDF) [file pone.0026566.s008.pdf]

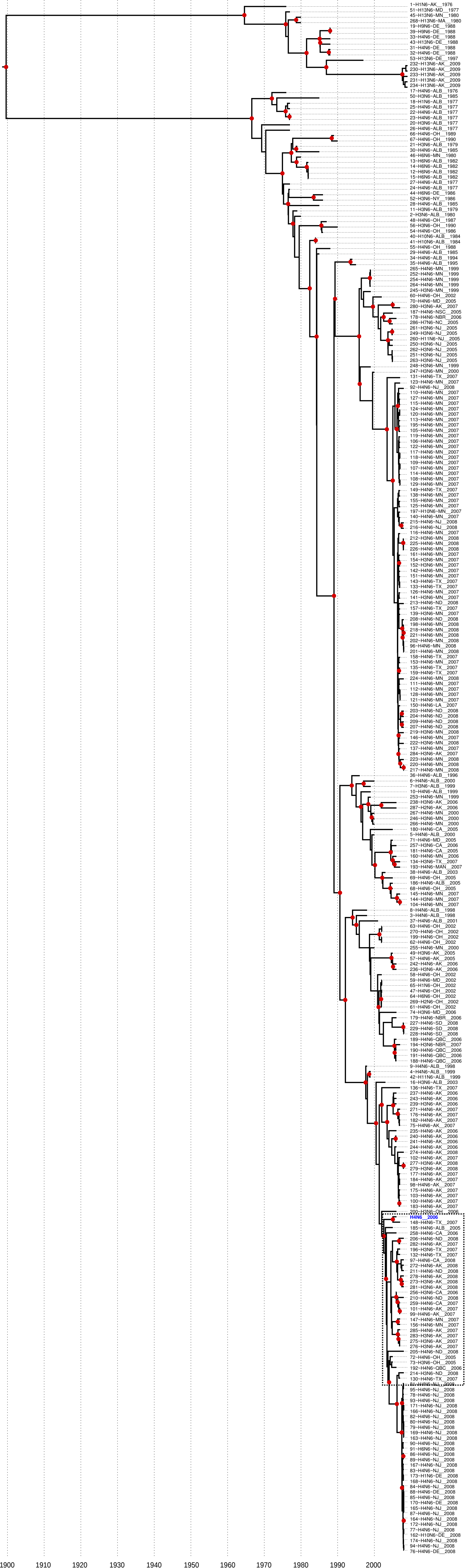

Supplement: Figure S9 — Maximum clade credibility tree for NA (N6) of viruses isolated in wild waterbirds in North America, between 1976 and 2009. Red dots represent nodes with posterior probability values superior to 0.95. The virus characterized in this study is colored in blue and the box represents the genetic sub-lineage detailed in Figure 1. Viral strain names and sequence accession numbers are listed in Table S3. (PDF) [file pone.0026566.s009.pdf]

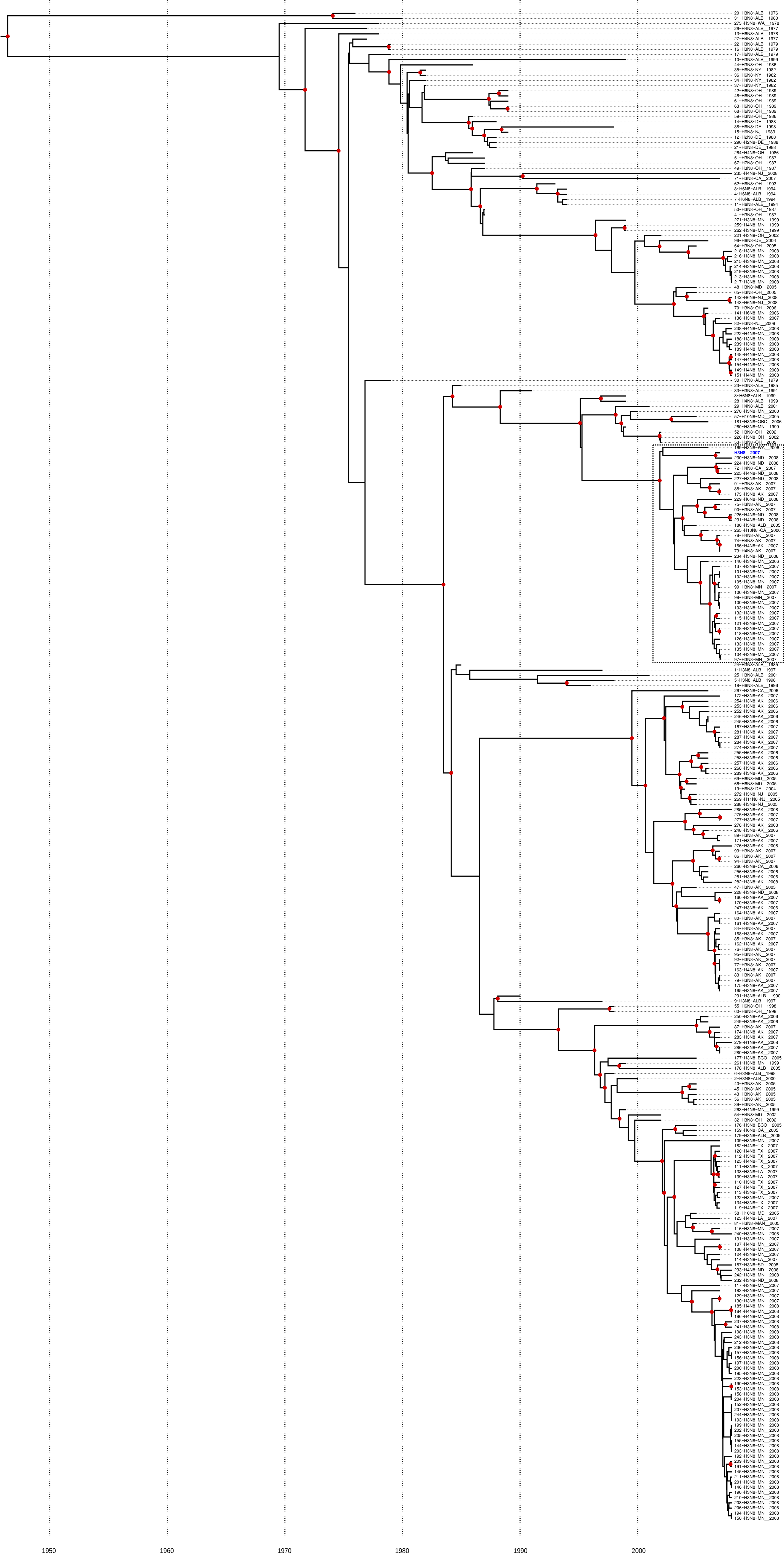

Supplement: Figure S10 — Maximum clade credibility tree for NA (N8) of viruses isolated in wild waterbirds in North America, between 1979 and 2007. Red dots represent nodes with posterior probability values superior to 0.95. The virus characterized in this study is colored in blue and the box represents the genetic sub-lineage detailed in Figure 1. Viral strain names and sequence accession numbers are listed in Table S3. (PDF) [file pone.0026566.s010.pdf]
