# Supplementary material for: Viral Replication, Persistence in Water and Genetic Characterization of Two Influenza A Viruses Isolated from Surface Lake Water
Source: PLoS One. 2011 Oct 20;6(10):e26566. doi: 10.1371/journal.pone.0026566 (PMC3197669; doi:10.1371/journal.pone.0026566)
Supplement: Table S1 — Adjusted R2 and P value obtained for linear regression models. (PDF) [file pone.0026566.s011.pdf]

A/Surface water/MN/W07-2241/2007 (H3N8)				A/Surface water/MN/NW1-T/2006 (H4N6)			
T (°C)	pH	R^2	P	T (°C)	pH	R^2	P
4	7.2	0.86	<0.001	4	7.2	0.78	<0.001
10	7.2	0.86	<0.001	10	7.2	0.84	<0.001
17	6.2	0.86	<0.001	17	6.2	0.83	<0.001
17	7.2	0.78	<0.001	17	7.2	0.61	<0.001
17	8.2	0.93	<0.001	17	8.2	0.77	<0.001
23	7.2	0.85	<0.001	23	7.2	0.86	<0.001
28	6.2	0.60	<0.01	28	6.2	0.97	<0.001
28	7.2	0.95	<0.001	28	7.2	0.92	<0.001
28	8.2	0.84	<0.001	28	8.2	0.91	<0.001
