# Supplementary material for: Viral Replication, Persistence in Water and Genetic Characterization of Two Influenza A Viruses Isolated from Surface Lake Water
Source: PLoS One. 2011 Oct 20;6(10):e26566. doi: 10.1371/journal.pone.0026566 (PMC3197669; doi:10.1371/journal.pone.0026566)
Supplement: Table S2 — Information related to nucleotide sequences used for phylogenetic analysis. (PDF) [file pone.0026566.s012.pdf]

Segment		N	Alignment length (bp)	Location	Time period
**PB2**		211	2280	MN	1979-2008
**PB1**		243	2277	MN	1979-2008
**PA**		215	2146	MN	1979-2008
**HA**	*H3*	275	1704	Canada: AB, BC, MB, NB, QC, SK.<br>USA: AK, CA, DE, LA, MD, MN, NC, ND, NJ, NY, OH, SD, TX, WA.	1976-2008
	H4	229	1695	Canada: AB, MB, NB, NS, QC.<br>USA: AK, CA, DE, LA, MD, MN, ND, NJ, NY, OH, SD, TX.	1977-2008
**NP**		75	1497	MN	1979-2007
**NA**	*N6*	288	1421	Canada: AB, MB, NB, NS, QC.<br>USA: AK, CA, DE, LA, MA, MD, MN, NC, ND, NJ, NY, OH, SD, TX.	1976-2009
	N8	292	1413	Canada: AB, BC, MB, QC.<br>USA: AK, CA, DE, LA, MD, MN, ND, NJ, NY, OH, SD, TX, WA.	1976-2008
**M**	M1	168	759	MN	1979-2008
**NS**		125	838	MN	1979-2007
