# Supplementary material for: Viral Replication, Persistence in Water and Genetic Characterization of Two Influenza A Viruses Isolated from Surface Lake Water
Source: PLoS One. 2011 Oct 20;6(10):e26566. doi: 10.1371/journal.pone.0026566 (PMC3197669; doi:10.1371/journal.pone.0026566)
Supplement: Table S3 — Viral strain names, accession numbers and taxa codes used in Figures S1, S2, S3, S4, S5, S6, S7, S8, S9, S10. (PDF) [file pone.0026566.s013.pdf]

Segment	Code in phylogenetic trees	Sequence accession number	Virus strain name
PB2	1~H13N6_1980	CY005865	A/gull/Minnesota/945/1980
PB2	10~H3N8_2007	CY032759	A/gadwall duck/Minnesota/Sg-00044/2007
PB2	100~H11N9_2007	CY042144	A/mallard/Minnesota/Sg-00179/2007
PB2	101~H3N6_2007	CY042153	A/mallard/Minnesota/Sg-00185/2007
PB2	102~H1N9_2007	CY042158	A/mallard/Minnesota/Sg-00186/2007
PB2	103~H10N3_2007	CY042173	A/mallard/Minnesota/Sg-00194/2007
PB2	104~H1N1_2007	CY042202	A/northern pintail/Minnesota/Sg-00216/2007
PB2	105~H1N1_2007	CY042206	A/mallard/Minnesota/Sg-00217/2007
PB2	106~H1N1_2006	CY042226	A/northern pintail/Minnesota/Sg-00227/2006
PB2	107~H4N6_2006	CY042236	A/blue-winged teal/Minnesota/Sg-00229/2006
PB2	108~H4N6_2007	CY042246	A/American wigeon/Minnesota/Sg-00231/2007
PB2	109~H4N8_2008	CY042333	A/ring-necked duck/Minnesota/Sg-00449/2008
PB2	11~H4N6_2007	CY032761	A/mallard duck/Minnesota/Sg-00045/2007
PB2	110~H4N8_2008	CY042337	A/blue-winged teal/Minnesota/Sg-00450/2008
PB2	111~H4N8_2008	CY042341	A/blue-winged teal/Minnesota/Sg-00451/2008
PB2	112~H4N2_2008	CY042345	A/blue-winged teal/Minnesota/Sg-00452/2008
PB2	113~H6N1_2008	CY042385	A/mallard/Minnesota/Sg-00462/2008
PB2	114~H3N8_2008	CY042389	A/mallard/Minnesota/Sg-00463/2008
PB2	115~H6N1_2008	CY042392	A/mallard/Minnesota/Sg-00464/2008
PB2	116~H4N8_2008	CY042396	A/mallard/Minnesota/Sg-00465/2008
PB2	117~H6N1_2008	CY042400	A/mallard/Minnesota/Sg-00467/2008
PB2	118~H3N1_2008	CY042611	A/mallard/Minnesota/Sg-00572/2008
PB2	119~H6N1_2008	CY042629	A/mallard/Minnesota/Sg-00576/2008
PB2	12~H4N6_2007	CY032763	A/mallard duck/Minnesota/Sg-00046/2007
PB2	120~H1N1_2008	CY042633	A/mallard/Minnesota/Sg-00579/2008
PB2	121~H3N8_2008	CY042637	A/mallard/Minnesota/Sg-00580/2008
PB2	122~H4N8_2008	CY042640	A/mallard/Minnesota/Sg-00624/2008
PB2	123~H4N8_2008	CY042645	A/mallard/Minnesota/Sg-00625/2008
PB2	124~H1N1_2008	CY042655	A/mallard/Minnesota/Sg-00627/2008
PB2	125~H1N1_2008	CY042659	A/mallard/Minnesota/Sg-00628/2008
PB2	126~H10N7_2008	CY042668	A/mallard/Minnesota/Sg-00630/2008
PB2	127~H4N8_2008	CY042672	A/mallard/Minnesota/Sg-00631/2008
PB2	128~H10N7_2008	CY042677	A/mallard/Minnesota/Sg-00632/2008
PB2	129~H4N8_2008	CY042690	A/mallard/Minnesota/Sg-00635/2008
PB2	13~H3N8_2007	CY032765	A/mallard duck/Minnesota/Sg-00047/2007
PB2	130~H4N4_2008	CY042739	A/northern shoveler/Minnesota/Sg-00646/2008
PB2	131~H4N6_2008	CY042741	A/northern shoveler/Minnesota/Sg-00647/2008
PB2	132~H8N4_2008	CY042745	A/northern shoveler/Minnesota/Sg-00648/2008
PB2	133~H1N1_2008	CY042757	A/northern shoveler/Minnesota/Sg-00651/2008
PB2	134~H1N1_2008	CY042773	A/northern shoveler/Minnesota/Sg-00655/2008
PB2	135~H3N8_2008	CY042832	A/mallard/Minnesota/Sg-00672/2008
PB2	136~H8N4_2008	CY042836	A/mallard/Minnesota/Sg-00673/2008
PB2	137~H8N4_2008	CY042844	A/mallard/Minnesota/Sg-00675/2008
PB2	138~H3N8_2008	CY042848	A/mallard/Minnesota/Sg-00676/2008
PB2	139~H3N8_2008	CY042852	A/mallard/Minnesota/Sg-00677/2008
PB2	14~H3N8_2007	CY032767	A/mallard duck/Minnesota/Sg-00048/2007
PB2	140~H8N4_2008	CY042856	A/mallard/Minnesota/Sg-00678/2008
PB2	141~H3N8_2008	CY042860	A/mallard/Minnesota/Sg-00679/2008
PB2	142~H3N4_2008	CY042863	A/mallard/Minnesota/Sg-00680/2008
PB2	143~H8N4_2008	CY042867	A/mallard/Minnesota/Sg-00681/2008
PB2	144~H8N4_2008	CY042871	A/mallard/Minnesota/Sg-00682/2008
PB2	145~H3N8_2008	CY042875	A/mallard/Minnesota/Sg-00683/2008
PB2	146~H8N4_2008	CY042879	A/mallard/Minnesota/Sg-00684/2008
PB2	147~H8N4_2008	CY042883	A/mallard/Minnesota/Sg-00685/2008
PB2	148~H3N8_2008	CY042891	A/mallard/Minnesota/Sg-00687/2008
PB2	149~H8N4_2008	CY042895	A/mallard/Minnesota/Sg-00688/2008
PB2	15~H4N6_2007	CY032769	A/mallard duck/Minnesota/Sg-00049/2007
PB2	150~H8N4_2008	CY042899	A/mallard/Minnesota/Sg-00689/2008
PB2	151~H8N4_2008	CY042903	A/mallard/Minnesota/Sg-00690/2008
PB2	152~H4N8_2008	CY042907	A/blue-winged teal/Minnesota/Sg-00691/2008
PB2	153~H2N3_2008	CY042911	A/mallard/Minnesota/Sg-00692/2008
PB2	154~H4N6_2008	CY042915	A/mallard/Minnesota/Sg-00693/2008
PB2	155~H4N6_2008	CY042919	A/mallard/Minnesota/Sg-00694/2008
PB2	156~H4N6_2008	CY042923	A/mallard/Minnesota/Sg-00695/2008
PB2	157~H4N6_2008	CY042931	A/mallard/Minnesota/Sg-00697/2008
PB2	158~H4N8_2008	CY042939	A/mallard/Minnesota/Sg-00700/2008
PB2	159~H8N4_2008	CY042943	A/mallard/Minnesota/Sg-00701/2008
PB2	16~H4N6_2007	CY032771	A/mallard duck/Minnesota/Sg-00050/2007
PB2	160~H3N1_1979	EF554800	A/mallard duck/Minnesota/1979
PB2	161~H11N9_2000	EU743464	A/mallard/Minnesota/42/2000
PB2	162~H11N9_2000	EU743472	A/mallard/Minnesota/107/2000
PB2	163~H11N9_2000	EU743479	A/mallard/Minnesota/109/2000
PB2	164~H3N2_1999	EU743500	A/mallard/Minnesota/68/1999

PB2	165~H3N2_1999	EU743507	A/mallard/Minnesota/290/1999
PB2	166~H3N2_1999	EU743515	A/mallard/Minnesota/380/1999
PB2	167~H3N1_1999	EU743523	A/mallard/MN/330/1999
PB2	168~H3N1_1999	EU743529	A/mallard/MN/351/1999
PB2	169~H3N5_1999	EU743535	A/mallard/Minnesota/280/1999
PB2	17~H3N8_2007	CY032774	A/mallard duck/Minnesota/Sg-00051/2007
PB2	170~H3N6_1999	EU743543	A/mallard/MN/259/1999
PB2	171~H3N6_2000	EU743551	A/mallard/MN/99/2000
PB2	172~H3N6_2000	EU743559	A/mallard/MN/515/2000
PB2	173~H3N6_1999	EU743567	A/pintail/Minnesota/479/1999
PB2	174~H3N9_2000	EU871875	A/mallard/MN/158/2000
PB2	175~H4N6_1999	EU871883	A/mallard/Minnesota/160/1999
PB2	176~H4N6_1999	EU871891	A/mallard/Minnesota/269/1999
PB2	177~H4N8_1999	EU871899	A/mallard/MN/323/1999
PB2	178~H5N2_2000	EU871906	A/mallard/MN/113/2000
PB2	179~H5N3_2000	EU871913	A/mallard/MN/479/2000
PB2	18~H4N6_2007	CY032776	A/mallard duck/Minnesota/Sg-00052/2007
PB2	180~H5N5_2000	EU871920	A/mallard/MN/105/2000
PB2	181~H5N2_2000	FJ357076	A/mallard/MN/1/2000
PB2	182~H2N3_1998	FJ357084	A/mallard/MN/14/1998
PB2	183~H2N3_1998	FJ357090	A/mallard/MN/51/1998
PB2	184~H2N3_2000	FJ517274	A/mallard/MN/506/2000
PB2	185~H3N8_1999	FJ517288	A/mallard/Minnesota/190/1999
PB2	186~H3N8_1999	FJ517295	A/mallard/Minnesota/283/1999
PB2	187~H3N8_1999	FJ517301	A/mallard/Minnesota/354/1999
PB2	188~H4N2_1998	FJ517305	A/mallard/Minnesota/371/1998
PB2	189~H4N6_1999	FJ517309	A/mallard/Minnesota/192/1999
PB2	19~H4N6_2007	CY032778	A/mallard duck/Minnesota/Sg-00053/2007
PB2	190~H4N6_2000	FJ517313	A/mallard/Minnesota/348/2000
PB2	191~H4N8_1999	FJ517321	A/mallard/MN/327/1999
PB2	192~H11N9_2000	GQ257380	A/mallard/Minnesota/249/2000
PB2	193~H5N2_2006	GQ923220	A/mallard/Minnesota/464334/2006
PB2	194~H2N2_1998	GU050730	A/mallard/Minnesota/26/1998
PB2	195~H2N2_1998	GU050736	A/mallard/Minnesota/27/1998
PB2	196~H2N1_2000	GU050750	A/mallard/Minnesota/550/2000
PB2	197~H2N9_1998	GU050806	A/mallard/Minnesota/34/1998
PB2	198~H2N9_2000	GU050813	A/mallard/Minnesota/31/2000
PB2	199~H3N1_2000	GU051134	A/mallard/Minnesota/417/2000
PB2	2~H6N6_1980	CY005888	A/blue-winged teal/MN/993/1980
PB2	20~H10N7_2007	CY032780	A/mallard duck/Minnesota/Sg-00056/2007
PB2	200~H3N2_2000	GU051146	A/mallard/Minnesota/282/2000
PB2	201~H4N2_1998	GU051211	A/mallard/Minnesota/95/1998
PB2	202~H4N6_1999	GU051231	A/mallard/Minnesota/145/1999
PB2	203~H4N6_1999	GU051239	A/mallard/Minnesota/195/1999
PB2	204~H4N8_1998	GU051257	A/mallard/Minnesota/114/1998
PB2	205~H4N8_1999	GU051274	A/mallard/Minnesota/212/1999
PB2	206~H4N8_1999	GU051280	A/mallard/Minnesota/237/1999
PB2	207~H4N8_1999	GU051287	A/mallard/Minnesota/271/1999
PB2	208~H5N1_2000	GU051308	A/mallard/Minnesota/24/2000
PB2	209~H5N2_2000	GU051323	A/mallard/Minnesota/283/2000
PB2	21~H10N7_2007	CY032783	A/mallard duck/Minnesota/Sg-00057/2007
PB2	210~H6N3_2000	GU051377	A/mallard/Minnesota/38/2000
PB2	211~H6N5_1999	GU051425	A/mallard/Minnesota/253/1999
PB2	212~H6N5_1999	GU051432	A/mallard/Minnesota/334/1999
PB2	22~H3N8_2007	CY032786	A/mallard duck/Minnesota/Sg-00059/2007
PB2	23~H4N6_2007	CY032789	A/mallard duck/Minnesota/Sg-00060/2007
PB2	24~H3N8_2007	CY032791	A/mallard duck/Minnesota/Sg-00061/2007
PB2	25~H8N4_2007	CY032793	A/mallard duck/Minnesota/Sg-00062/2007
PB2	26~H4N6_2007	CY032795	A/mallard duck/Minnesota/Sg-00063/2007
PB2	27~H3N8_2007	CY032797	A/mallard duck/Minnesota/Sg-00064/2007
PB2	28~H10N7_2007	CY032800	A/mallard duck/Minnesota/Sg-00065/2007
PB2	29~H3N8_2007	CY032802	A/ring-necked duck/Minnesota/Sg-00066/2007
PB2	3~H10N7_1979	CY014746	A/mallard duck/Minnesota/19/1979
PB2	30~H4N6_2007	CY032803	A/ring-necked duck/Minnesota/Sg-00067/2007
PB2	31~H10N7_2007	CY032804	A/ring-necked duck/Minnesota/Sg-00068/2007
PB2	32~H3N8_2007	CY032807	A/ring-necked duck/Minnesota/Sg-00069/2007
PB2	33~H4N8_2007	CY032810	A/mallard duck/Minnesota/Sg-00070/2007
PB2	34~H4N8_2007	CY032813	A/mallard duck/Minnesota/Sg-00071/2007
PB2	35~H4N6_2007	CY032844	A/blue-winged teal/Minnesota/Sg-00095/2007
PB2	36~H3N8_2007	CY032846	A/mallard duck/Minnesota/Sg-00097/2007
PB2	37~H3N6_2007	CY032847	A/mallard duck/Minnesota/Sg-00098/2007
PB2	38~H3N3_2007	CY032851	A/mallard duck/Minnesota/Sg-00100/2007
PB2	39~H10N3_2007	CY032852	A/mallard duck/Minnesota/Sg-00101/2007
PB2	4~H4N6_2007	CY032745	A/blue-winged teal/Minnesota/Sg-00038/2007

PB2	40~H3N8_2007	CY032856	A/mallard duck/Minnesota/Sg-00103/2007
PB2	41~H6N1_2007	CY032858	A/mallard duck/Minnesota/Sg-00105/2007
PB2	43~H1N9_2007	CY032863	A/mallard duck/Minnesota/Sg-00113/2007
PB2	44~H10N7_2007	CY032865	A/mallard duck/Minnesota/Sg-00114/2007
PB2	45~H11N9_2007	CY032867	A/mallard duck/Minnesota/Sg-00115/2007
PB2	46~H3N6_2007	CY032868	A/mallard duck/Minnesota/Sg-00116/2007
PB2	47~H11N9_2007	CY032871	A/mallard duck/Minnesota/Sg-00118/2007
PB2	48~H4N6_2007	CY032873	A/mallard duck/Minnesota/Sg-00120/2007
PB2	49~H4N6_2007	CY033657	A/blue-winged teal/Minnesota/Sg-00028/2007
PB2	5~H4N6_2007	CY032747	A/blue-winged teal/Minnesota/Sg-00039/2007
PB2	50~H4N6_2007	CY033661	A/blue-winged teal/Minnesota/Sg-00029/2007
PB2	51~H4N6_2007	CY033664	A/blue-winged teal/Minnesota/Sg-00030/2007
PB2	52~H4N6_2007	CY033668	A/blue-winged teal/Minnesota/Sg-00031/2007
PB2	53~H4N6_2007	CY033669	A/blue-winged teal/Minnesota/Sg-00032/2007
PB2	54~H4N6_2007	CY033672	A/blue-winged teal/Minnesota/Sg-00033/2007
PB2	55~H4N6_2007	CY033674	A/blue-winged teal/Minnesota/Sg-00034/2007
PB2	56~H4N6_2007	CY033676	A/blue-winged teal/Minnesota/Sg-00035/2007
PB2	57~H4N6_2007	CY033678	A/blue-winged teal/Minnesota/Sg-00036/2007
PB2	58~H4N6_2007	CY033696	A/mallard duck/Minnesota/Sg-00054/2007
PB2	59~H12N5_2007	CY033697	A/mallard duck/Minnesota/Sg-00055/2007
PB2	6~H4N6_2007	CY032750	A/blue-winged teal/Minnesota/Sg-00040/2007
PB2	60~H4N6_2007	CY033701	A/mallard duck/Minnesota/Sg-00058/2007
PB2	61~H4N6_2007	CY033751	A/mallard duck/Minnesota/Sg-00096/2007
PB2	62~H1N3_2007	CY033762	A/mallard/Minnesota/Sg-00108/2007
PB2	63~H1N3_2007	CY033765	A/mallard/Minnesota/Sg-00111/2007
PB2	64~H3N8_2007	CY033772	A/mallard duck/Minnesota/Sg-00117/2007
PB2	65~H3N8_2007	CY033776	A/mallard duck/Minnesota/Sg-00119/2007
PB2	66~H1N1_2007	CY033784	A/mallard duck/Minnesota/Sg-00122/2007
PB2	67~H3N8_2007	CY034625	A/blue-winged teal/Minnesota/Sg-00037/2007
PB2	68~H3N8_2007	CY034643	A/mallard duck/Minnesota/Sg-00072/2007
PB2	69~H11N9_2007	CY034674	A/mallard duck/Minnesota/Sg-00110/2007
PB2	7~H4N6_2007	CY032751	A/blue-winged teal/Minnesota/Sg-00041/2007
PB2	70~H3N8_2007	CY035268	A/mallard/Minnesota/Sg-00161/2007
PB2	71~H3N8_2007	CY035274	A/mallard/Minnesota/Sg-00164/2007
PB2	72~H6N1_2007	CY035279	A/mallard/Minnesota/Sg-00167/2007
PB2	74~H10N6_2007	CY035289	A/mallard/Minnesota/Sg-00172/2007
PB2	75~H3N6_2007	CY035296	A/mallard/Minnesota/Sg-00174/2007
PB2	76~H4N6_2007	CY035300	A/mallard/Minnesota/Sg-00176/2007
PB2	77~H10N3_2007	CY035305	A/mallard/Minnesota/Sg-00178/2007
PB2	78~H10N7_2007	CY035309	A/mallard/Minnesota/Sg-00181/2007
PB2	79~H11N2_2007	CY035318	A/mallard/Minnesota/Sg-00187/2007
PB2	8~H4N6_2007	CY032754	A/blue-winged teal/Minnesota/Sg-00042/2007
PB2	80~H3N8_2007	CY035325	A/mallard/Minnesota/Sg-00190/2007
PB2	81~H3N8_2007	CY035328	A/mallard/Minnesota/Sg-00191/2007
PB2	82~H3N8_2007	CY035331	A/mallard/Minnesota/Sg-00192/2007
PB2	83~H6N6_2007	CY035335	A/northern pintail/Minnesota/Sg-00196/2007
PB2	84~H6N2_2007	CY035341	A/green-winged teal/Minnesota/Sg-00199/2007
PB2	85~H3N8_2007	CY035349	A/mallard/Minnesota/Sg-00202/2007
PB2	86~H3N8_2007	CY035353	A/mallard/Minnesota/Sg-00203/2007
PB2	87~H4N6_2007	CY035372	A/mallard/Minnesota/Sg-00210/2007
PB2	88~H3N8_2007	CY035376	A/mallard/Minnesota/Sg-00211/2007
PB2	89~H11N9_2007	CY035383	A/green-winged teal/Minnesota/Sg-00213/2007
PB2	9~H4N6_2007	CY032757	A/blue-winged teal/Minnesota/Sg-00043/2007
PB2	90~H3N8_2007	CY035388	A/mallard/Minnesota/Sg-00215/2007
PB2	92~H10N7_2007	CY035403	A/mallard/Minnesota/Sg-00221/2007
PB2	93~H6N2_2007	CY035405	A/green-winged teal/Minnesota/Sg-00222/2007
PB2	94~H6N1_2007	CY035408	A/mallard/Minnesota/Sg-00223/2007
PB2	95~H4N6_2008	CY038253	A/mallard/Minnesota/Sg-00569/2008
PB2	96~H8N4_2008	CY038258	A/mallard/Minnesota/Sg-00570/2008
PB2	97~H3N2_2007	CY041884	A/green-winged teal/Minnesota/Sg-00131/2007
PB2	98~H4N6_2007	CY041892	A/mallard/Minnesota/Sg-00133/2007
PB2	99~H4N6_2007	CY042136	A/mallard/Minnesota/Sg-00171/2007
PB1	1~H13N6_1980	CY005864	A/gull/Minnesota/945/1980
PB1	10~H4N6_2007	CY032746	A/blue-winged teal/Minnesota/Sg-00038/2007
PB1	100~H10N7_2007	CY035404	A/mallard/Minnesota/Sg-00221/2007
PB1	101~H6N2_2007	CY035406	A/green-winged teal/Minnesota/Sg-00222/2007
PB1	103~H4N6_2008	CY038254	A/mallard/Minnesota/Sg-00569/2008
PB1	104~H8N4_2008	CY038259	A/mallard/Minnesota/Sg-00570/2008
PB1	105~H3N2_2007	CY041861	A/northern pintail/South Dakota/Sg-00126/2007
PB1	107~H3N2_2007	CY041877	A/mallard/South Dakota/Sg-00128/2007
PB1	108~H3N2_2007	CY041885	A/green-winged teal/Minnesota/Sg-00131/2007
PB1	109~H4N6_2007	CY041893	A/mallard/Minnesota/Sg-00133/2007
PB1	11~H4N6_2007	CY032748	A/blue-winged teal/Minnesota/Sg-00039/2007
PB1	111~H6N1_2007	CY042071	A/mallard duck/Minnesota/Sg-00104/2007

PB1	112~H1N3_2007	CY042123	A/mallard/Minnesota/Sg-00162/2007
PB1	114~H4N6_2007	CY042137	A/mallard/Minnesota/Sg-00171/2007
PB1	117~H1N9_2007	CY042159	A/mallard/Minnesota/Sg-00186/2007
PB1	12~H4N6_2007	CY032752	A/blue-winged teal/Minnesota/Sg-00041/2007
PB1	122~H6N5_2006	CY042217	A/blue-winged teal/Minnesota/Sg-00225/2006
PB1	123~H3N8_2006	CY042222	A/blue-winged teal/Minnesota/Sg-00226/2006
PB1	124~H1N1_2006	CY042227	A/northern pintail/Minnesota/Sg-00227/2006
PB1	125~H6N8_2006	CY042232	A/green-winged teal/Minnesota/Sg-00228/2006
PB1	126~H4N6_2006	CY042237	A/blue-winged teal/Minnesota/Sg-00229/2006
PB1	127~H7N3_2006	CY042242	A/mallard/Minnesota/Sg-00230/2006
PB1	128~H4N6_2007	CY042247	A/American wigeon/Minnesota/Sg-00231/2007
PB1	129~H1N1_2008	CY042326	A/mallard/Minnesota/Sg-00447/2008
PB1	130~H6N1_2008	CY042330	A/mallard/Minnesota/Sg-00448/2008
PB1	131~H4N8_2008	CY042334	A/ring-necked duck/Minnesota/Sg-00449/2008
PB1	132~H4N8_2008	CY042338	A/blue-winged teal/Minnesota/Sg-00450/2008
PB1	133~H4N8_2008	CY042342	A/blue-winged teal/Minnesota/Sg-00451/2008
PB1	134~H4N2_2008	CY042346	A/blue-winged teal/Minnesota/Sg-00452/2008
PB1	135~H3N8_2008	CY042350	A/blue-winged teal/Minnesota/Sg-00453/2008
PB1	136~H6N1_2008	CY042386	A/mallard/Minnesota/Sg-00462/2008
PB1	137~H6N1_2008	CY042393	A/mallard/Minnesota/Sg-00464/2008
PB1	138~H3N8_2008	CY042616	A/mallard/Minnesota/Sg-00573/2008
PB1	139~H3N8_2008	CY042621	A/mallard/Minnesota/Sg-00574/2008
PB1	140~H1N1_2008	CY042634	A/mallard/Minnesota/Sg-00579/2008
PB1	141~H3N8_2008	CY042638	A/mallard/Minnesota/Sg-00580/2008
PB1	142~H4N8_2008	CY042641	A/mallard/Minnesota/Sg-00624/2008
PB1	143~H4N8_2008	CY042646	A/mallard/Minnesota/Sg-00625/2008
PB1	144~H4N8_2008	CY042651	A/mallard/Minnesota/Sg-00626/2008
PB1	145~H1N1_2008	CY042656	A/mallard/Minnesota/Sg-00627/2008
PB1	146~H1N1_2008	CY042660	A/mallard/Minnesota/Sg-00628/2008
PB1	147~H3N8_2008	CY042664	A/mallard/Minnesota/Sg-00629/2008
PB1	148~H10N7_2008	CY042669	A/mallard/Minnesota/Sg-00630/2008
PB1	149~H4N8_2008	CY042673	A/mallard/Minnesota/Sg-00631/2008
PB1	15~H3N8_2007	CY032760	A/gadwall duck/Minnesota/Sg-00044/2007
PB1	150~H10N7_2008	CY042678	A/mallard/Minnesota/Sg-00632/2008
PB1	151~H3N8_2008	CY042682	A/mallard/Minnesota/Sg-00633/2008
PB1	152~H3N8_2008	CY042686	A/mallard/Minnesota/Sg-00634/2008
PB1	153~H4N8_2008	CY042691	A/mallard/Minnesota/Sg-00635/2008
PB1	154~H3N8_2008	CY042696	A/mallard/Minnesota/Sg-00636/2008
PB1	155~H3N8_2008	CY042701	A/mallard/Minnesota/Sg-00637/2008
PB1	156~H3N8_2008	CY042706	A/mallard/Minnesota/Sg-00638/2008
PB1	157~H3N8_2008	CY042711	A/mallard/Minnesota/Sg-00639/2008
PB1	158~H3N8_2008	CY042716	A/mallard/Minnesota/Sg-00640/2008
PB1	159~H3N8_2008	CY042720	A/mallard/Minnesota/Sg-00641/2008
PB1	16~H4N6_2007	CY032762	A/mallard duck/Minnesota/Sg-00045/2007
PB1	160~H3N8_2008	CY042724	A/mallard/Minnesota/Sg-00642/2008
PB1	161~H3N8_2008	CY042728	A/mallard/Minnesota/Sg-00643/2008
PB1	162~H3N8_2008	CY042732	A/mallard/Minnesota/Sg-00644/2008
PB1	163~H3N8_2008	CY042736	A/northern shoveler/Minnesota/Sg-00645/2008
PB1	164~H4N6_2008	CY042742	A/northern shoveler/Minnesota/Sg-00647/2008
PB1	165~H8N4_2008	CY042746	A/northern shoveler/Minnesota/Sg-00648/2008
PB1	166~H3N8_2008	CY042750	A/blue-winged teal/Minnesota/Sg-00649/2008
PB1	167~H3N8_2008	CY042754	A/blue-winged teal/Minnesota/Sg-00650/2008
PB1	168~H1N1_2008	CY042758	A/northern shoveler/Minnesota/Sg-00651/2008
PB1	169~H3N8_2008	CY042762	A/northern shoveler/Minnesota/Sg-00652/2008
PB1	17~H3N8_2007	CY032766	A/mallard duck/Minnesota/Sg-00047/2007
PB1	170~H3N8_2008	CY042766	A/northern shoveler/Minnesota/Sg-00653/2008
PB1	171~H3N8_2008	CY042770	A/northern shoveler/Minnesota/Sg-00654/2008
PB1	172~H1N1_2008	CY042774	A/northern shoveler/Minnesota/Sg-00655/2008
PB1	173~H3N8_2008	CY042778	A/northern shoveler/Minnesota/Sg-00656/2008
PB1	174~H3N8_2008	CY042782	A/northern shoveler/Minnesota/Sg-00657/2008
PB1	175~H3N8_2008	CY042786	A/northern shoveler/Minnesota/Sg-00658/2008
PB1	176~H3N8_2008	CY042790	A/northern shoveler/Minnesota/Sg-00659/2008
PB1	177~H3N8_2008	CY042794	A/northern shoveler/Minnesota/Sg-00660/2008
PB1	178~H3N8_2008	CY042800	A/northern shoveler/Minnesota/Sg-00662/2008
PB1	179~H3N8_2008	CY042804	A/northern shoveler/Minnesota/Sg-00663/2008
PB1	18~H3N8_2007	CY032768	A/mallard duck/Minnesota/Sg-00048/2007
PB1	180~H3N8_2008	CY042813	A/northern shoveler/Minnesota/Sg-00667/2008
PB1	181~H3N8_2008	CY042817	A/northern shoveler/Minnesota/Sg-00668/2008
PB1	182~H3N8_2008	CY042821	A/northern shoveler/Minnesota/Sg-00669/2008
PB1	183~H3N8_2008	CY042825	A/northern shoveler/Minnesota/Sg-00670/2008
PB1	184~H3N8_2008	CY042829	A/northern shoveler/Minnesota/Sg-00671/2008
PB1	185~H3N8_2008	CY042833	A/mallard/Minnesota/Sg-00672/2008
PB1	186~H8N4_2008	CY042837	A/mallard/Minnesota/Sg-00673/2008
PB1	187~H3N8_2008	CY042841	A/mallard/Minnesota/Sg-00674/2008

PB1	188~H8N4_2008	CY042845	A/mallard/Minnesota/Sg-00675/2008
PB1	189~H3N8_2008	CY042849	A/mallard/Minnesota/Sg-00676/2008
PB1	19~H4N6_2007	CY032770	A/mallard duck/Minnesota/Sg-00049/2007
PB1	190~H3N8_2008	CY042853	A/mallard/Minnesota/Sg-00677/2008
PB1	191~H8N4_2008	CY042857	A/mallard/Minnesota/Sg-00678/2008
PB1	192~H3N8_2008	CY042861	A/mallard/Minnesota/Sg-00679/2008
PB1	193~H3N4_2008	CY042864	A/mallard/Minnesota/Sg-00680/2008
PB1	194~H8N4_2008	CY042868	A/mallard/Minnesota/Sg-00681/2008
PB1	195~H8N4_2008	CY042872	A/mallard/Minnesota/Sg-00682/2008
PB1	196~H3N8_2008	CY042876	A/mallard/Minnesota/Sg-00683/2008
PB1	197~H8N4_2008	CY042880	A/mallard/Minnesota/Sg-00684/2008
PB1	198~H8N4_2008	CY042884	A/mallard/Minnesota/Sg-00685/2008
PB1	199~H8N4_2008	CY042888	A/mallard/Minnesota/Sg-00686/2008
PB1	2~H6N6_1980	CY005887	A/blue-winged teal/MN/993/1980
PB1	200~H3N8_2008	CY042892	A/mallard/Minnesota/Sg-00687/2008
PB1	201~H8N4_2008	CY042896	A/mallard/Minnesota/Sg-00688/2008
PB1	202~H8N4_2008	CY042900	A/mallard/Minnesota/Sg-00689/2008
PB1	203~H8N4_2008	CY042904	A/mallard/Minnesota/Sg-00690/2008
PB1	204~H4N8_2008	CY042908	A/blue-winged teal/Minnesota/Sg-00691/2008
PB1	205~H2N3_2008	CY042912	A/mallard/Minnesota/Sg-00692/2008
PB1	206~H4N6_2008	CY042916	A/mallard/Minnesota/Sg-00693/2008
PB1	207~H4N6_2008	CY042920	A/mallard/Minnesota/Sg-00694/2008
PB1	208~H4N6_2008	CY042924	A/mallard/Minnesota/Sg-00695/2008
PB1	209~H3N8_2008	CY042928	A/mallard/Minnesota/Sg-00696/2008
PB1	21~H4N6_2007	CY032777	A/mallard duck/Minnesota/Sg-00052/2007
PB1	210~H4N6_2008	CY042932	A/mallard/Minnesota/Sg-00697/2008
PB1	211~H4N8_2008	CY042936	A/mallard/Minnesota/Sg-00698/2008
PB1	212~H4N8_2008	CY042940	A/mallard/Minnesota/Sg-00700/2008
PB1	213~H8N4_2008	CY042944	A/mallard/Minnesota/Sg-00701/2008
PB1	214~H3N1_1979	EF554801	A/mallard duck/Minnesota/1979
PB1	215~H11N9_2000	EU743463	A/mallard/Minnesota/42/2000
PB1	216~H11N9_2000	EU743471	A/mallard/Minnesota/107/2000
PB1	217~H11N9_2000	EU743478	A/mallard/Minnesota/109/2000
PB1	218~H3N2_1999	EU743499	A/mallard/Minnesota/68/1999
PB1	219~H3N2_1999	EU743506	A/mallard/Minnesota/290/1999
PB1	220~H3N2_1999	EU743514	A/mallard/Minnesota/380/1999
PB1	221~H3N1_1999	EU743522	A/mallard/MN/330/1999
PB1	222~H3N1_1999	EU743528	A/mallard/MN/351/1999
PB1	223~H3N5_1999	EU743534	A/mallard/Minnesota/280/1999
PB1	224~H3N6_1999	EU743542	A/mallard/MN/259/1999
PB1	225~H3N6_2000	EU743550	A/mallard/MN/99/2000
PB1	226~H3N6_2000	EU743558	A/mallard/MN/515/2000
PB1	227~H3N6_1999	EU743566	A/pintail/Minnesota/479/1999
PB1	228~H3N9_2000	EU871874	A/mallard/MN/158/2000
PB1	229~H4N6_1999	EU871882	A/mallard/Minnesota/160/1999
PB1	230~H4N6_1999	EU871890	A/mallard/Minnesota/269/1999
PB1	231~H4N8_1999	EU871898	A/mallard/MN/323/1999
PB1	232~H5N2_2000	EU871905	A/mallard/MN/113/2000
PB1	233~H5N3_2000	EU871912	A/mallard/MN/479/2000
PB1	234~H5N5_2000	EU871919	A/mallard/MN/105/2000
PB1	235~H5N2_2000	FJ357075	A/mallard/MN/1/2000
PB1	236~H2N3_1998	FJ357083	A/mallard/MN/14/1998
PB1	237~H2N3_1998	FJ357089	A/mallard/MN/51/1998
PB1	238~H2N3_2000	FJ517273	A/mallard/MN/506/2000
PB1	239~H3N8_1999	FJ517287	A/mallard/Minnesota/190/1999
PB1	24~H10N7_2007	CY032784	A/mallard duck/Minnesota/Sg-00057/2007
PB1	240~H3N8_1999	FJ517294	A/mallard/Minnesota/283/1999
PB1	241~H3N8_1999	FJ517300	A/mallard/Minnesota/354/1999
PB1	242~H4N2_1998	FJ517304	A/mallard/Minnesota/371/1998
PB1	243~H4N6_1999	FJ517308	A/mallard/Minnesota/192/1999
PB1	244~H4N6_2000	FJ517312	A/mallard/Minnesota/348/2000
PB1	245~H4N8_1999	FJ517320	A/mallard/MN/327/1999
PB1	246~H11N9_2000	GQ257379	A/mallard/Minnesota/249/2000
PB1	247~H5N2_2006	GQ923219	A/mallard/Minnesota/464334/2006
PB1	248~H2N2_1998	GU050729	A/mallard/Minnesota/26/1998
PB1	249~H2N2_1998	GU050735	A/mallard/Minnesota/27/1998
PB1	250~H2N1_2000	GU050749	A/mallard/Minnesota/550/2000
PB1	251~H2N9_1998	GU050805	A/mallard/Minnesota/34/1998
PB1	252~H2N9_2000	GU050812	A/mallard/Minnesota/31/2000
PB1	253~H10N7_2000	GU050888	A/mallard/Minnesota/518/2000
PB1	254~H3N1_2000	GU051133	A/mallard/Minnesota/417/2000
PB1	255~H3N2_2000	GU051145	A/mallard/Minnesota/282/2000
PB1	256~H3N8_2000	GU051152	A/mallard/Minnesota/448/2000
PB1	257~H3N8_1999	GU051184	A/mallard/Minnesota/231/1999

PB1	258~H3N9_1998	GU051205	A/mallard/Minnesota/182/1998
PB1	259~H4N2_1998	GU051210	A/mallard/Minnesota/95/1998
PB1	26~H4N6_2007	CY032790	A/mallard duck/Minnesota/Sg-00060/2007
PB1	260~H4N3_2000	GU051216	A/mallard/Minnesota/14/2000
PB1	261~H4N6_1998	GU051225	A/mallard/Minnesota/2/1998
PB1	262~H4N6_1999	GU051228	A/mallard/Minnesota/140/1999
PB1	263~H4N6_1999	GU051236	A/mallard/Minnesota/193/1999
PB1	264~H4N6_1999	GU051238	A/mallard/Minnesota/195/1999
PB1	265~H4N6_2000	GU051245	A/mallard/Minnesota/313/2000
PB1	266~H4N8_1998	GU051256	A/mallard/Minnesota/114/1998
PB1	267~H4N8_1999	GU051261	A/mallard/Minnesota/168/1999
PB1	268~H4N8_1999	GU051266	A/mallard/Minnesota/188/1999
PB1	269~H4N8_1999	GU051271	A/mallard/Minnesota/210/1999
PB1	27~H3N8_2007	CY032792	A/mallard duck/Minnesota/Sg-00061/2007
PB1	270~H4N8_1999	GU051273	A/mallard/Minnesota/212/1999
PB1	271~H4N8_1999	GU051279	A/mallard/Minnesota/237/1999
PB1	272~H4N8_1999	GU051286	A/mallard/Minnesota/271/1999
PB1	273~H4N8_1999	GU051292	A/mallard/Minnesota/402/1999
PB1	274~H4N9_1999	GU051295	A/mallard/Minnesota/263/1999
PB1	275~H5N1_2000	GU051307	A/mallard/Minnesota/24/2000
PB1	276~H5N2_2000	GU051313	A/mallard/Minnesota/166/2000
PB1	277~H5N2_2000	GU051318	A/mallard/Minnesota/168/2000
PB1	278~H5N2_2000	GU051322	A/mallard/Minnesota/283/2000
PB1	279~H5N2_2000	GU051328	A/mallard/Minnesota/410/2000
PB1	280~H5N3_2000	GU051337	A/mallard/Minnesota/382/2000
PB1	281~H6N3_2000	GU051376	A/mallard/Minnesota/38/2000
PB1	282~H6N4_1999	GU051386	A/mallard/Minnesota/313/1999
PB1	283~H6N5_1998	GU051421	A/mallard/Minnesota/66/1998
PB1	284~H6N5_1999	GU051424	A/mallard/Minnesota/253/1999
PB1	285~H6N5_1999	GU051430	A/mallard/Minnesota/329/1999
PB1	286~H6N6_1998	GU051438	A/mallard/Minnesota/179/1998
PB1	287~H6N8_1998	GU051442	A/mallard/Minnesota/29/1998
PB1	288~H6N8_1998	GU051448	A/mallard/Minnesota/63/1998
PB1	289~H6N8_1998	GU051452	A/mallard/Minnesota/353/1998
PB1	290~H6N8_1999	GU051456	A/mallard/Minnesota/173/1999
PB1	291~H6N8_1999	GU051458	A/mallard/Minnesota/175/1999
PB1	292~H6N8_1999	GU051463	A/mallard/Minnesota/204/1999
PB1	293~H6N8_1999	GU051464	A/pintail/Minnesota/410/1999
PB1	294~H9N2_1998	GU051522	A/mallard/Minnesota/231/1998
PB1	295~H9N2_1998	GU051525	A/mallard/Minnesota/250/1998
PB1	296~H9N2_1998	GU051527	A/mallard/Minnesota/309/1998
PB1	297~H9N2_1999	GU051530	A/mallard/Minnesota/166/1999
PB1	298~H9N2_1999	GU051531	A/mallard/Minnesota/186/1999
PB1	299~H9N2_1999	GU051534	A/mallard/Minnesota/341/1999
PB1	3~H10N7_1979	CY014745	A/mallard duck/Minnesota/19/1979
PB1	30~H3N8_2007	CY032798	A/mallard duck/Minnesota/Sg-00064/2007
PB1	34~H4N8_2007	CY032814	A/mallard duck/Minnesota/Sg-00071/2007
PB1	36~H4N6_2007	CY032845	A/blue-winged teal/Minnesota/Sg-00095/2007
PB1	41~H6N1_2007	CY032859	A/mallard duck/Minnesota/Sg-00105/2007
PB1	45~H10N7_2007	CY032866	A/mallard duck/Minnesota/Sg-00114/2007
PB1	47~H4N6_2007	CY032874	A/mallard duck/Minnesota/Sg-00120/2007
PB1	49~H4N6_2007	CY033662	A/blue-winged teal/Minnesota/Sg-00029/2007
PB1	5~H4N6_2007	CY032736	A/blue-winged teal/Minnesota/Sg-00032/2007
PB1	52~H4N6_2007	CY033682	A/blue-winged teal/Minnesota/Sg-00040/2007
PB1	53~H4N6_2007	CY033689	A/mallard duck/Minnesota/Sg-00046/2007
PB1	57~H4N6_2007	CY033709	A/ring-necked duck/Minnesota/Sg-00067/2007
PB1	58~H4N6_2007	CY033752	A/mallard duck/Minnesota/Sg-00096/2007
PB1	62~H11N9_2007	CY033770	A/mallard duck/Minnesota/Sg-00115/2007
PB1	63~H3N8_2007	CY033773	A/mallard duck/Minnesota/Sg-00117/2007
PB1	64~H11N9_2007	CY033775	A/mallard/Minnesota/Sg-00118/2007
PB1	67~H1N1_2007	CY033785	A/mallard duck/Minnesota/Sg-00122/2007
PB1	68~H3N2_2007	CY033790	A/mallard/South Dakota/Sg-00125/2007
PB1	69~H3N8_2007	CY035266	A/mallard/Minnesota/Sg-00160/2007
PB1	7~H4N6_2007	CY032740	A/blue-winged teal/Minnesota/Sg-00035/2007
PB1	72~H6N1_2007	CY035280	A/mallard/Minnesota/Sg-00167/2007
PB1	75~H10N6_2007	CY035290	A/mallard/Minnesota/Sg-00172/2007
PB1	76~H3N6_2007	CY035297	A/mallard/Minnesota/Sg-00174/2007
PB1	77~H4N6_2007	CY035301	A/mallard/Minnesota/Sg-00176/2007
PB1	79~H10N3_2007	CY035306	A/mallard/Minnesota/Sg-00178/2007
PB1	80~H10N7_2007	CY035310	A/mallard/Minnesota/Sg-00181/2007
PB1	82~H3N8_2007	CY035316	A/mallard/Minnesota/Sg-00184/2007
PB1	83~H11N2_2007	CY035319	A/mallard/Minnesota/Sg-00187/2007
PB1	84~H3N8_2007	CY035326	A/mallard/Minnesota/Sg-00190/2007
PB1	85~H3N8_2007	CY035329	A/mallard/Minnesota/Sg-00191/2007

PB1	86~H3N8_2007	CY035332	A/mallard/Minnesota/Sg-00192/2007
PB1	87~H6N6_2007	CY035336	A/northern pintail/Minnesota/Sg-00196/2007
PB1	89~H6N2_2007	CY035342	A/green-winged teal/Minnesota/Sg-00199/2007
PB1	9~H3N8_2007	CY032744	A/blue-winged teal/Minnesota/Sg-00037/2007
PB1	92~H3N8_2007	CY035350	A/mallard/Minnesota/Sg-00202/2007
PB1	94~H4N6_2007	CY035373	A/mallard/Minnesota/Sg-00210/2007
PB1	99~H6N1_2007	CY035400	A/mallard/Minnesota/Sg-00220/2007
PA	1~H13N6_1980	CY005863	A/gull/Minnesota/945/1980
PA	10~H3N8_2007	CY033759	A/mallard duck/Minnesota/Sg-00103/2007
PA	100~H3N8_2008	CY042787	A/northern shoveler/Minnesota/Sg-00658/2008
PA	101~H3N8_2008	CY042791	A/northern shoveler/Minnesota/Sg-00659/2008
PA	102~H3N8_2008	CY042795	A/northern shoveler/Minnesota/Sg-00660/2008
PA	103~H3N8_2008	CY042801	A/northern shoveler/Minnesota/Sg-00662/2008
PA	104~H3N8_2008	CY042805	A/northern shoveler/Minnesota/Sg-00663/2008
PA	105~H3N8_2008	CY042809	A/northern shoveler/Minnesota/Sg-00664/2008
PA	106~H3N8_2008	CY042814	A/northern shoveler/Minnesota/Sg-00667/2008
PA	107~H3N8_2008	CY042818	A/northern shoveler/Minnesota/Sg-00668/2008
PA	108~H3N8_2008	CY042822	A/northern shoveler/Minnesota/Sg-00669/2008
PA	109~H3N8_2008	CY042826	A/northern shoveler/Minnesota/Sg-00670/2008
PA	11~H11N9_2007	CY033764	A/mallard/Minnesota/Sg-00110/2007
PA	110~H3N8_2008	CY042830	A/northern shoveler/Minnesota/Sg-00671/2008
PA	111~H3N8_2008	CY042834	A/mallard/Minnesota/Sg-00672/2008
PA	112~H8N4_2008	CY042838	A/mallard/Minnesota/Sg-00673/2008
PA	113~H3N8_2008	CY042842	A/mallard/Minnesota/Sg-00674/2008
PA	114~H8N4_2008	CY042846	A/mallard/Minnesota/Sg-00675/2008
PA	115~H3N8_2008	CY042850	A/mallard/Minnesota/Sg-00676/2008
PA	116~H3N8_2008	CY042854	A/mallard/Minnesota/Sg-00677/2008
PA	117~H8N4_2008	CY042858	A/mallard/Minnesota/Sg-00678/2008
PA	118~H3N4_2008	CY042865	A/mallard/Minnesota/Sg-00680/2008
PA	119~H8N4_2008	CY042869	A/mallard/Minnesota/Sg-00681/2008
PA	12~H11N9_2007	CY033767	A/mallard/Minnesota/Sg-00112/2007
PA	120~H8N4_2008	CY042873	A/mallard/Minnesota/Sg-00682/2008
PA	121~H3N8_2008	CY042877	A/mallard/Minnesota/Sg-00683/2008
PA	122~H8N4_2008	CY042881	A/mallard/Minnesota/Sg-00684/2008
PA	123~H8N4_2008	CY042885	A/mallard/Minnesota/Sg-00685/2008
PA	124~H8N4_2008	CY042889	A/mallard/Minnesota/Sg-00686/2008
PA	125~H3N8_2008	CY042893	A/mallard/Minnesota/Sg-00687/2008
PA	126~H8N4_2008	CY042897	A/mallard/Minnesota/Sg-00688/2008
PA	127~H8N4_2008	CY042901	A/mallard/Minnesota/Sg-00689/2008
PA	128~H8N4_2008	CY042905	A/mallard/Minnesota/Sg-00690/2008
PA	129~H4N8_2008	CY042909	A/blue-winged teal/Minnesota/Sg-00691/2008
PA	13~H10N1_2007	CY033769	A/mallard duck/Minnesota/Sg-00114/2007
PA	130~H2N3_2008	CY042913	A/mallard/Minnesota/Sg-00692/2008
PA	131~H4N6_2008	CY042917	A/mallard/Minnesota/Sg-00693/2008
PA	132~H4N6_2008	CY042921	A/mallard/Minnesota/Sg-00694/2008
PA	133~H4N6_2008	CY042925	A/mallard/Minnesota/Sg-00695/2008
PA	134~H3N8_2008	CY042929	A/mallard/Minnesota/Sg-00696/2008
PA	135~H4N6_2008	CY042933	A/mallard/Minnesota/Sg-00697/2008
PA	136~H4N8_2008	CY042937	A/mallard/Minnesota/Sg-00698/2008
PA	137~H4N8_2008	CY042941	A/mallard/Minnesota/Sg-00700/2008
PA	138~H8N4_2008	CY042945	A/mallard/Minnesota/Sg-00701/2008
PA	139~H3N1_1979	EF554802	A/mallard duck/Minnesota/1979
PA	14~H1N1_2007	CY033786	A/mallard/Minnesota/Sg-00122/2007
PA	140~H11N9_2000	EU743462	A/mallard/Minnesota/42/2000
PA	141~H11N9_2000	EU743470	A/mallard/Minnesota/107/2000
PA	142~H11N9_2000	EU743477	A/mallard/Minnesota/109/2000
PA	143~H3N2_1999	EU743498	A/mallard/Minnesota/68/1999
PA	144~H3N2_1999	EU743505	A/mallard/Minnesota/290/1999
PA	145~H3N2_1999	EU743513	A/mallard/Minnesota/380/1999
PA	146~H3N1_1999	EU743521	A/mallard/MN/330/1999
PA	147~H3N1_1999	EU743527	A/mallard/MN/351/1999
PA	148~H3N5_1999	EU743533	A/mallard/Minnesota/280/1999
PA	149~H3N6_1999	EU743541	A/mallard/MN/259/1999
PA	15~H3N8_2007	CY034629	A/mallard duck/Minnesota/Sg-00047/2007
PA	150~H3N6_2000	EU743549	A/mallard/MN/99/2000
PA	151~H3N6_2000	EU743557	A/mallard/MN/515/2000
PA	152~H3N6_1999	EU743565	A/pintail/Minnesota/479/1999
PA	153~H3N9_2000	EU871873	A/mallard/MN/158/2000
PA	154~H4N6_1999	EU871881	A/mallard/Minnesota/160/1999
PA	155~H4N6_1999	EU871889	A/mallard/Minnesota/269/1999
PA	156~H4N8_1999	EU871897	A/mallard/MN/323/1999
PA	157~H5N2_2000	EU871904	A/mallard/MN/113/2000
PA	158~H5N3_2000	EU871911	A/mallard/MN/479/2000
PA	159~H5N5_2000	EU871918	A/mallard/MN/105/2000

PA	16~H4N6_2007	CY034631	A/mallard duck/Minnesota/Sg-00049/2007
PA	160~H5N2_2000	FJ357074	A/mallard/MN/1/2000
PA	161~H2N3_1998	FJ357082	A/mallard/MN/14/1998
PA	162~H2N3_1998	FJ357088	A/mallard/MN/51/1998
PA	163~H2N3_2000	FJ517272	A/mallard/MN/506/2000
PA	164~H3N8_1999	FJ517286	A/mallard/Minnesota/190/1999
PA	165~H3N8_1999	FJ517293	A/mallard/Minnesota/283/1999
PA	166~H3N8_1999	FJ517299	A/mallard/Minnesota/354/1999
PA	167~H4N2_1998	FJ517303	A/mallard/Minnesota/371/1998
PA	168~H4N6_1999	FJ517307	A/mallard/Minnesota/192/1999
PA	169~H4N6_2000	FJ517311	A/mallard/Minnesota/348/2000
PA	17~H4N6_2007	CY034633	A/mallard duck/Minnesota/Sg-00053/2007
PA	170~H4N8_1999	FJ517319	A/mallard/MN/327/1999
PA	171~H11N9_2000	GQ257378	A/mallard/Minnesota/249/2000
PA	172~H5N2_2006	GQ923218	A/mallard/Minnesota/464334/2006
PA	173~H2N2_1998	GU050728	A/mallard/Minnesota/26/1998
PA	174~H2N2_1998	GU050734	A/mallard/Minnesota/27/1998
PA	175~H2N1_2000	GU050748	A/mallard/Minnesota/550/2000
PA	176~H2N9_1998	GU050804	A/mallard/Minnesota/34/1998
PA	177~H2N9_2000	GU050811	A/mallard/Minnesota/31/2000
PA	178~H10N7_2000	GU050887	A/mallard/Minnesota/518/2000
PA	179~H3N1_2000	GU051132	A/mallard/Minnesota/417/2000
PA	18~H4N6_2007	CY034637	A/mallard duck/Minnesota/Sg-00060/2007
PA	180~H3N2_2000	GU051144	A/mallard/Minnesota/282/2000
PA	181~H3N9_1998	GU051204	A/mallard/Minnesota/182/1998
PA	182~H4N2_1998	GU051209	A/mallard/Minnesota/95/1998
PA	183~H4N3_2000	GU051215	A/mallard/Minnesota/14/2000
PA	184~H4N6_1999	GU051227	A/mallard/Minnesota/140/1999
PA	185~H4N6_1999	GU051230	A/mallard/Minnesota/145/1999
PA	186~H4N6_1999	GU051235	A/mallard/Minnesota/193/1999
PA	187~H4N6_1999	GU051237	A/mallard/Minnesota/195/1999
PA	188~H4N6_2000	GU051244	A/mallard/Minnesota/313/2000
PA	189~H4N6_2000	GU051250	A/mallard/Minnesota/524/2000
PA	19~H8N4_2007	CY034640	A/mallard duck/Minnesota/Sg-00062/2007
PA	190~H4N8_1998	GU051255	A/mallard/Minnesota/114/1998
PA	191~H4N8_1999	GU051265	A/mallard/Minnesota/188/1999
PA	192~H4N8_1999	GU051270	A/mallard/Minnesota/210/1999
PA	193~H4N8_1999	GU051272	A/mallard/Minnesota/212/1999
PA	194~H4N8_1999	GU051278	A/mallard/Minnesota/237/1999
PA	195~H4N8_1999	GU051285	A/mallard/Minnesota/271/1999
PA	196~H4N8_1999	GU051291	A/mallard/Minnesota/402/1999
PA	197~H4N9_1999	GU051294	A/mallard/Minnesota/263/1999
PA	198~H5N1_2000	GU051306	A/mallard/Minnesota/24/2000
PA	199~H5N2_2000	GU051312	A/mallard/Minnesota/166/2000
PA	2~H9N2_1980	CY005878	A/goose/MN/5733-1/1980
PA	20~H3N8_2007	CY034659	A/mallard duck/Minnesota/Sg-00097/2007
PA	200~H5N2_2000	GU051317	A/mallard/Minnesota/168/2000
PA	201~H5N2_2000	GU051321	A/mallard/Minnesota/283/2000
PA	202~H5N2_2000	GU051327	A/mallard/Minnesota/410/2000
PA	203~H5N3_2000	GU051336	A/mallard/Minnesota/382/2000
PA	204~H6N3_2000	GU051375	A/mallard/Minnesota/38/2000
PA	205~H6N4_1999	GU051385	A/mallard/Minnesota/313/1999
PA	206~H6N5_1998	GU051420	A/mallard/Minnesota/66/1998
PA	207~H6N5_1998	GU051422	A/mallard/Minnesota/157/1998
PA	208~H6N5_1999	GU051423	A/mallard/Minnesota/253/1999
PA	209~H6N5_1999	GU051429	A/mallard/Minnesota/329/1999
PA	21~H3N6_2007	CY034660	A/mallard duck/Minnesota/Sg-00098/2007
PA	210~H6N5_1999	GU051431	A/mallard/Minnesota/334/1999
PA	211~H6N6_1998	GU051437	A/mallard/Minnesota/179/1998
PA	212~H6N8_1998	GU051443	A/mallard/Minnesota/31/1998
PA	213~H6N8_1998	GU051447	A/mallard/Minnesota/63/1998
PA	214~H6N8_1998	GU051451	A/mallard/Minnesota/353/1998
PA	215~H6N8_1999	GU051457	A/mallard/Minnesota/175/1999
PA	216~H6N8_1999	GU051462	A/mallard/Minnesota/204/1999
PA	22~H3N3_2007	CY034664	A/mallard duck/Minnesota/Sg-00100/2007
PA	23~H10N3_2007	CY034665	A/mallard duck/Minnesota/Sg-00101/2007
PA	24~H6N1_2007	CY034668	A/mallard duck/Minnesota/Sg-00105/2007
PA	25~H6N2_2007	CY034670	A/mallard duck/Minnesota/Sg-00106/2007
PA	27~H11N9_2007	CY034676	A/mallard/Minnesota/Sg-00115/2007
PA	29~H10N6_2007	CY035291	A/mallard/Minnesota/Sg-00172/2007
PA	3~H6N6_1980	CY005886	A/blue-winged teal/MN/993/1980
PA	30~H10N3_2007	CY035307	A/mallard/Minnesota/Sg-00178/2007
PA	31~H6N1_2007	CY035314	A/mallard/Minnesota/Sg-00182/2007
PA	32~H3N8_2007	CY035327	A/mallard/Minnesota/Sg-00190/2007

PA	33~H6N6_2007	CY035337	A/Northern pintail/Minnesota/Sg-00196/2007
PA	34~H3N8_2007	CY035345	A/mallard/Minnesota/Sg-00200/2007
PA	35~H3N8_2007	CY035390	A/mallard/Minnesota/Sg-00215/2007
PA	37~H4N6_2008	CY038255	A/mallard/Minnesota/Sg-00569/2008
PA	38~H8N4_2008	CY038260	A/mallard/Minnesota/Sg-00570/2008
PA	39~H3N2_2007	CY041886	A/green-winged teal/Minnesota/Sg-00131/2007
PA	4~H10N7_1979	CY014744	A/mallard duck/Minnesota/19/1979
PA	40~H4N6_2007	CY041894	A/mallard/Minnesota/Sg-00133/2007
PA	41~H1N3_2007	CY042076	A/mallard/Minnesota/Sg-00108/2007
PA	42~H1N1_2007	CY042094	A/redheaded duck/Minnesota/Sg-00123/2007
PA	43~H3N8_2007	CY042194	A/mallard/Minnesota/Sg-00209/2007
PA	44~H6N5_2006	CY042218	A/blue-winged teal/Minnesota/Sg-00225/2006
PA	45~H3N8_2006	CY042223	A/blue-winged teal/Minnesota/Sg-00226/2006
PA	46~H1N1_2006	CY042228	A/northern pintail/Minnesota/Sg-00227/2006
PA	47~H6N8_2006	CY042233	A/green-winged teal/Minnesota/Sg-00228/2006
PA	48~H4N6_2006	CY042238	A/blue-winged teal/Minnesota/Sg-00229/2006
PA	49~H7N3_2006	CY042243	A/mallard/Minnesota/Sg-00230/2006
PA	5~H4N6_2007	CY033666	A/blue-winged teal/Minnesota/Sg-00030/2007
PA	50~H4N6_2007	CY042248	A/American wigeon/Minnesota/Sg-00231/2007
PA	51~H1N1_2008	CY042327	A/mallard/Minnesota/Sg-00447/2008
PA	52~H6N1_2008	CY042331	A/mallard/Minnesota/Sg-00448/2008
PA	53~H4N8_2008	CY042335	A/ring-necked duck/Minnesota/Sg-00449/2008
PA	54~H4N8_2008	CY042339	A/blue-winged teal/Minnesota/Sg-00450/2008
PA	55~H4N8_2008	CY042343	A/blue-winged teal/Minnesota/Sg-00451/2008
PA	56~H4N2_2008	CY042347	A/blue-winged teal/Minnesota/Sg-00452/2008
PA	57~H3N8_2008	CY042351	A/blue-winged teal/Minnesota/Sg-00453/2008
PA	58~H6N1_2008	CY042387	A/mallard/Minnesota/Sg-00462/2008
PA	59~H6N1_2008	CY042394	A/mallard/Minnesota/Sg-00464/2008
PA	6~H4N6_2007	CY033686	A/blue-winged teal/Minnesota/Sg-00043/2007
PA	60~H4N8_2008	CY042398	A/mallard/Minnesota/Sg-00465/2008
PA	61~H6N1_2008	CY042402	A/mallard/Minnesota/Sg-00467/2008
PA	62~H3N1_2008	CY042612	A/mallard/Minnesota/Sg-00572/2008
PA	63~H3N8_2008	CY042617	A/mallard/Minnesota/Sg-00573/2008
PA	64~H3N8_2008	CY042622	A/mallard/Minnesota/Sg-00574/2008
PA	65~H3N8_2008	CY042626	A/gadwall duck/Minnesota/Sg-00575/2008
PA	66~H6N1_2008	CY042631	A/mallard/Minnesota/Sg-00576/2008
PA	67~H1N1_2008	CY042635	A/mallard/Minnesota/Sg-00579/2008
PA	68~H3N8_2008	CY042639	A/mallard/Minnesota/Sg-00580/2008
PA	69~H4N8_2008	CY042642	A/mallard/Minnesota/Sg-00624/2008
PA	7~H12N5_2007	CY033699	A/mallard duck/Minnesota/Sg-00055/2007
PA	70~H4N8_2008	CY042652	A/mallard/Minnesota/Sg-00626/2008
PA	71~H1N1_2008	CY042657	A/mallard/Minnesota/Sg-00627/2008
PA	72~H1N1_2008	CY042661	A/mallard/Minnesota/Sg-00628/2008
PA	73~H10N7_2008	CY042670	A/mallard/Minnesota/Sg-00630/2008
PA	74~H4N8_2008	CY042674	A/mallard/Minnesota/Sg-00631/2008
PA	75~H10N7_2008	CY042679	A/mallard/Minnesota/Sg-00632/2008
PA	76~H3N8_2008	CY042683	A/mallard/Minnesota/Sg-00633/2008
PA	77~H3N8_2008	CY042687	A/mallard/Minnesota/Sg-00634/2008
PA	78~H4N8_2008	CY042692	A/mallard/Minnesota/Sg-00635/2008
PA	79~H3N8_2008	CY042697	A/mallard/Minnesota/Sg-00636/2008
PA	8~H3N8_2007	CY033704	A/mallard duck/Minnesota/Sg-00061/2007
PA	80~H3N8_2008	CY042702	A/mallard/Minnesota/Sg-00637/2008
PA	81~H3N8_2008	CY042707	A/mallard/Minnesota/Sg-00638/2008
PA	82~H3N8_2008	CY042712	A/mallard/Minnesota/Sg-00639/2008
PA	83~H3N8_2008	CY042717	A/mallard/Minnesota/Sg-00640/2008
PA	84~H3N8_2008	CY042721	A/mallard/Minnesota/Sg-00641/2008
PA	85~H3N8_2008	CY042725	A/mallard/Minnesota/Sg-00642/2008
PA	86~H3N8_2008	CY042729	A/mallard/Minnesota/Sg-00643/2008
PA	87~H3N8_2008	CY042733	A/mallard/Minnesota/Sg-00644/2008
PA	88~H3N8_2008	CY042737	A/northern shoveler/Minnesota/Sg-00645/2008
PA	89~H4N6_2008	CY042743	A/northern shoveler/Minnesota/Sg-00647/2008
PA	9~H4N6_2007	CY033750	A/blue-winged teal/Minnesota/Sg-00095/2007
PA	90~H8N4_2008	CY042747	A/northern shoveler/Minnesota/Sg-00648/2008
PA	91~H3N8_2008	CY042751	A/blue-winged teal/Minnesota/Sg-00649/2008
PA	92~H3N8_2008	CY042755	A/blue-winged teal/Minnesota/Sg-00650/2008
PA	93~H1N1_2008	CY042759	A/northern shoveler/Minnesota/Sg-00651/2008
PA	94~H3N8_2008	CY042763	A/northern shoveler/Minnesota/Sg-00652/2008
PA	95~H3N8_2008	CY042767	A/northern shoveler/Minnesota/Sg-00653/2008
PA	96~H3N8_2008	CY042771	A/northern shoveler/Minnesota/Sg-00654/2008
PA	97~H1N1_2008	CY042775	A/northern shoveler/Minnesota/Sg-00655/2008
PA	98~H3N8_2008	CY042779	A/northern shoveler/Minnesota/Sg-00656/2008
PA	99~H3N8_2008	CY042783	A/northern shoveler/Minnesota/Sg-00657/2008
HA (H3)	1~H3N8~ALB_1997	AY633132	A/mallard/Alberta/117/97
HA (H3)	10~H3N8~ALB_1985	CY004670	A/blue-winged teal/ALB/569/1985

HA (H3)	100~H3N8~MN_2007	CY035391	A/mallard/Minnesota/Sg-00215/2007
HA (H3)	101~H3N8~LA_2007	CY035395	A/blue-winged teal/Louisiana/Sg-00218/2007
HA (H3)	102~H3N8~AK_2007	CY035799	A/mallard/Interior Alaska/2/2007
HA (H3)	103~H3N8~AK_2007	CY035807	A/American widgeon/Interior Alaska/1/2007
HA (H3)	104~H3N8~AK_2007	CY035815	A/northern shoveler/Interior Alaska/1/2007
HA (H3)	105~H3N8~AK_2007	CY035877	A/mallard/Interior Alaska/1/2007
HA (H3)	106~H3N8~AK_2007	CY036659	A/green-winged teal/Interior Alaska/1/2007
HA (H3)	107~H3N8~MAN_2005	CY037007	A/blue-winged teal/Manitoba/13436/2005
HA (H3)	108~H3N2~DE_2008	CY038031	A/ruddy turnstone/Delaware/Sg-00469/2008
HA (H3)	109~H3N2~NJ_2008	CY038156	A/ruddy turnstone/New Jersey/Sg-00513/2008
HA (H3)	11~H3N5~ALB_1995	CY004692	A/mallard/Alberta/118/1995
HA (H3)	110~H3N2~NJ_2008	CY038161	A/ruddy turnstone/New Jersey/Sg-00518/2008
HA (H3)	111~H3N7~NJ_2008	CY038166	A/ruddy turnstone/New Jersey/Sg-00522/2008
HA (H3)	112~H3N8~NJ_2008	CY038171	A/ruddy turnstone/New Jersey/Sg-00525/2008
HA (H3)	113~H3N8~AK_2007	CY038362	A/mallard/Interior Alaska/3/2007
HA (H3)	114~H3N8~AK_2007	CY038378	A/mallard/Interior Alaska/4/2007
HA (H3)	115~H3N8~AK_2007	CY038386	A/American green-winged teal/Interior Alaska/3/2007
HA (H3)	116~H3N1~CA_2007	CY039620	A/northern shoveler/California/HKWF569/2007
HA (H3)	117~H3N8~AK_2007	CY039755	A/mallard/Interior Alaska/6/2007
HA (H3)	118~H3N8~AK_2007	CY039763	A/northern shoveler/Interior Alaska/2/2007
HA (H3)	119~H3N8~AK_2007	CY039778	A/northern pintail/Interior Alaska/1/2007
HA (H3)	12~H3N8~ALB_2001	CY004702	A/mallard/Alberta/156/2001
HA (H3)	120~H3N8~AK_2007	CY039794	A/northern shoveler/Interior Alaska/3/2007
HA (H3)	121~H3N8~AK_2007	CY039802	A/American green-winged teal/Interior Alaska/4/2007
HA (H3)	122~H3N8~AK_2007	CY039818	A/mallard/Interior Alaska/5/2007
HA (H3)	123~H3N8~AK_2007	CY039826	A/northern shoveler/Interior Alaska/4/2007
HA (H3)	124~H3N8~AK_2007	CY039850	A/mallard/Interior Alaska/7/2007
HA (H3)	125~H3N8~AK_2007	CY039873	A/mallard/Interior Alaska/8/2007
HA (H3)	126~H3N2~SD_2007	CY041855	A/mallard/South Dakota/Sg-00125/2007
HA (H3)	127~H3N2~SD_2007	CY041863	A/northern pintail/South Dakota/Sg-00126/2007
HA (H3)	128~H3N2~SD_2007	CY041871	A/mallard/South Dakota/Sg-00127/2007
HA (H3)	129~H3N2~SD_2007	CY041879	A/mallard/South Dakota/Sg-00128/2007
HA (H3)	13~H3N8~ALB_1979	CY005915	A/mallard duck/ALB/564/1979
HA (H3)	130~H3N2~MN_2007	CY041887	A/green-winged teal/Minnesota/Sg-00131/2007
HA (H3)	131~H3N8~MN_2007	CY042063	A/mallard duck/Minnesota/Sg-00097/2007
HA (H3)	132~H3N8~MN_2007	CY042087	A/mallard duck/Minnesota/Sg-00117/2007
HA (H3)	133~H3N8~MN_2007	CY042119	A/mallard/Minnesota/Sg-00160/2007
HA (H3)	134~H3N8~MN_2007	CY042121	A/mallard/Minnesota/Sg-00161/2007
HA (H3)	135~H3N8~MN_2007	CY042133	A/mallard/Minnesota/Sg-00168/2007
HA (H3)	136~H3N6~MN_2007	CY042156	A/mallard/Minnesota/Sg-00185/2007
HA (H3)	137~H3N8~LA_2007	CY042167	A/blue-winged teal/Louisiana/Sg-00189/2007
HA (H3)	138~H3N8~MN_2007	CY042168	A/mallard/Minnesota/Sg-00190/2007
HA (H3)	139~H3N8~MN_2007	CY042170	A/mallard/Minnesota/Sg-00191/2007
HA (H3)	14~H3N6~ALB_1979	CY005916	A/mallard duck/ALB/676/1979
HA (H3)	140~H3N8~MN_2007	CY042195	A/mallard/Minnesota/Sg-00209/2007
HA (H3)	141~H3N1~SD_2008	CY042324	A/blue-winged teal/South Dakota/Sg-00446/2008
HA (H3)	142~H3N8~MN_2008	CY042352	A/blue-winged teal/Minnesota/Sg-00453/2008
HA (H3)	143~H3N8~SD_2008	CY042384	A/northern pintail/South Dakota/Sg-00461/2008
HA (H3)	144~H3N8~MN_2008	CY042391	A/mallard/Minnesota/Sg-00463/2008
HA (H3)	145~H3N2~NJ_2008	CY042495	A/ruddy turnstone/New Jersey/Sg-00519/2008
HA (H3)	146~H3N2~NJ_2008	CY042514	A/ruddy turnstone/New Jersey/Sg-00529/2008
HA (H3)	147~H3N1~MN_2008	CY042613	A/mallard/Minnesota/Sg-00572/2008
HA (H3)	148~H3N8~MN_2008	CY042618	A/mallard/Minnesota/Sg-00573/2008
HA (H3)	149~H3N8~MN_2008	CY042627	A/gadwall duck/Minnesota/Sg-00575/2008
HA (H3)	15~H3N8~ALB_1976	CY005917	A/mallard duck/ALB/31/1976
HA (H3)	150~H3N8~MN_2008	CY042666	A/mallard/Minnesota/Sg-00629/2008
HA (H3)	151~H3N8~MN_2008	CY042688	A/mallard/Minnesota/Sg-00634/2008
HA (H3)	152~H3N8~MN_2008	CY042698	A/mallard/Minnesota/Sg-00636/2008
HA (H3)	153~H3N8~MN_2008	CY042703	A/mallard/Minnesota/Sg-00637/2008
HA (H3)	154~H3N8~MN_2008	CY042708	A/mallard/Minnesota/Sg-00638/2008
HA (H3)	155~H3N8~MN_2008	CY042722	A/mallard/Minnesota/Sg-00641/2008
HA (H3)	156~H3N8~MN_2008	CY042730	A/mallard/Minnesota/Sg-00643/2008
HA (H3)	157~H3N8~MN_2008	CY042734	A/mallard/Minnesota/Sg-00644/2008
HA (H3)	158~H3N8~MN_2008	CY042738	A/northern shoveler/Minnesota/Sg-00645/2008
HA (H3)	159~H3N8~MN_2008	CY042752	A/blue-winged teal/Minnesota/Sg-00649/2008
HA (H3)	16~H3N2~ALB_1976	CY005935	A/pintail duck/ALB/86/1976
HA (H3)	160~H3N8~MN_2008	CY042756	A/blue-winged teal/Minnesota/Sg-00650/2008
HA (H3)	161~H3N8~MN_2008	CY042768	A/northern shoveler/Minnesota/Sg-00653/2008
HA (H3)	162~H3N8~MN_2008	CY042772	A/northern shoveler/Minnesota/Sg-00654/2008
HA (H3)	163~H3N8~MN_2008	CY042780	A/northern shoveler/Minnesota/Sg-00656/2008
HA (H3)	164~H3N8~MN_2008	CY042788	A/northern shoveler/Minnesota/Sg-00658/2008
HA (H3)	165~H3N8~MN_2008	CY042792	A/northern shoveler/Minnesota/Sg-00659/2008
HA (H3)	166~H3N8~MN_2008	CY042796	A/northern shoveler/Minnesota/Sg-00660/2008
HA (H3)	167~H3N1~MN_2008	CY042798	A/northern shoveler/Minnesota/Sg-00661/2008

HA (H3)	168~H3N8~MN_2008	CY042802	A/northern shoveler/Minnesota/Sg-00662/2008
HA (H3)	169~H3N8~MN_2008	CY042806	A/northern shoveler/Minnesota/Sg-00663/2008
HA (H3)	17~H3N3~ALB_1978	CY005936	A/mallard duck/ALB/712/1978
HA (H3)	170~H3N8~MN_2008	CY042810	A/northern shoveler/Minnesota/Sg-00664/2008
HA (H3)	171~H3N1~MN_2008	CY042811	A/northern shoveler/Minnesota/Sg-00665/2008
HA (H3)	172~H3N8~MN_2008	CY042815	A/northern shoveler/Minnesota/Sg-00667/2008
HA (H3)	173~H3N8~MN_2008	CY042819	A/northern shoveler/Minnesota/Sg-00668/2008
HA (H3)	174~H3N8~MN_2008	CY042823	A/northern shoveler/Minnesota/Sg-00669/2008
HA (H3)	175~H3N8~MN_2008	CY042827	A/northern shoveler/Minnesota/Sg-00670/2008
HA (H3)	176~H3N8~MN_2008	CY042831	A/northern shoveler/Minnesota/Sg-00671/2008
HA (H3)	177~H3N8~MN_2008	CY042835	A/mallard/Minnesota/Sg-00672/2008
HA (H3)	178~H3N8~MN_2008	CY042843	A/mallard/Minnesota/Sg-00674/2008
HA (H3)	179~H3N8~MN_2008	CY042851	A/mallard/Minnesota/Sg-00676/2008
HA (H3)	18~H3N8~ALB_1979	CY005937	A/green-winged teal/ALB/438/1979
HA (H3)	180~H3N8~MN_2008	CY042855	A/mallard/Minnesota/Sg-00677/2008
HA (H3)	181~H3N8~MN_2008	CY042862	A/mallard/Minnesota/Sg-00679/2008
HA (H3)	182~H3N8~MN_2008	CY042878	A/mallard/Minnesota/Sg-00683/2008
HA (H3)	183~H3N8~MN_2008	CY042894	A/mallard/Minnesota/Sg-00687/2008
HA (H3)	184~H3N8~MN_2008	CY042930	A/mallard/Minnesota/Sg-00696/2008
HA (H3)	185~H3N8~ND_2008	CY042958	A/blue-winged teal/North Dakota/Sg-00704/2008
HA (H3)	186~H3N8~ND_2008	CY042986	A/blue-winged teal/North Dakota/Sg-00714/2008
HA (H3)	187~H3N8~ND_2008	CY043022	A/blue-winged teal/North Dakota/Sg-00745/2008
HA (H3)	188~H3N8~ND_2008	CY043023	A/blue-winged teal/North Dakota/Sg-00746/2008
HA (H3)	189~H3N6~MN_2008	CY043027	A/mallard/Minnesota/Sg-00751/2008
HA (H3)	19~H3N6~ALB_1979	CY005938	A/pintail duck/ALB/462/1979
HA (H3)	190~H3N6~ND_2008	CY043034	A/blue-winged teal/North Dakota/Sg-00759/2008
HA (H3)	191~H3N8~MN_2008	CY043039	A/mallard/Minnesota/Sg-00764/2008
HA (H3)	192~H3N8~MN_2008	CY043040	A/mallard/Minnesota/Sg-00765/2008
HA (H3)	193~H3N8~MN_2008	CY043051	A/green-winged teal/Minnesota/Sg-00779/2008
HA (H3)	194~H3N6~MN_2008	CY043052	A/blue-winged teal/Minnesota/Sg-00780/2008
HA (H3)	195~H3N2~MN_2008	CY043054	A/blue-winged teal/Minnesota/Sg-00787/2008
HA (H3)	196~H3N8~MN_2008	CY043055	A/blue-winged teal/Minnesota/Sg-00789/2008
HA (H3)	197~H3N8~MN_2008	CY043056	A/blue-winged teal/Minnesota/Sg-00790/2008
HA (H3)	198~H3N2~MN_2008	CY043061	A/blue-winged teal/Minnesota/Sg-00797/2008
HA (H3)	199~H3N8~MN_2008	CY043068	A/mallard/Minnesota/Sg-00804/2008
HA (H3)	2~H3N8~ALB_2000	AY633148	A/mallard/Alberta/127/00
HA (H3)	20~H3N2~ALB_1979	CY005939	A/pintail duck/ALB/627/1979
HA (H3)	200~H3N8~MN_2008	CY043069	A/mallard/Minnesota/Sg-00805/2008
HA (H3)	201~H3N8~MN_2008	CY043072	A/mallard/Minnesota/Sg-00808/2008
HA (H3)	202~H3N8~AK_2007	CY043936	A/northern pintail/Alaska/7MP0344/2007
HA (H3)	203~H3N8~AK_2007	CY043944	A/northern pintail/Alaska/7MP0608/2007
HA (H3)	204~H3N8~AK_2007	CY043960	A/northern shoveler/Alaska/7MP1026/2007
HA (H3)	205~H3N8~AK_2007	CY043976	A/American widgeon/Alaska/7MP1061/2007
HA (H3)	206~H3N8~AK_2007	CY043984	A/northern pintail/Alaska/7MP1092/2007
HA (H3)	207~H3N8~AK_2007	CY044008	A/northern shoveler/Alaska/7MP1606/2007
HA (H3)	208~H3N8~AK_2007	CY044024	A/northern shoveler/Alaska/7MP1668/2007
HA (H3)	209~H3N3~QUB_2005	CY045271	A/mallard/Quebec/16485/2005
HA (H3)	21~H3N1~ALB_1983	CY005940	A/blue-winged teal/ALB/452/1983
HA (H3)	210~H3N2~SAS_2007	CY045319	A/northern pintail/Saskatchewan/22910/2007
HA (H3)	211~H3N8~WA_2006	CY045367	A/mallard/Washington/20010-002/2006
HA (H3)	212~H3N8~AK_2007	CY045407	A/northern pintail/Interior Alaska/7MP0343/2007
HA (H3)	213~H3N8~AK_2007	CY045431	A/mallard/Interior Alaska/7MP0709/2007
HA (H3)	214~H3N8~AK_2007	CY045439	A/northern shoveler/Alaska/7MP1708/2007
HA (H3)	215~H3N8~AK_2007	CY045447	A/northern pintail/Alaska/7MP0508/2007
HA (H3)	216~H3N8~AK_2007	CY047027	A/American green-winged teal/Interior Alaska/7MP2225/2007
HA (H3)	217~H3N8~AK_2007	CY047035	A/mallard/Interior Alaska/7MP1757/2007
HA (H3)	218~H3N8~BCO_2005	CY047488	A/mallard/British Columbia/07706/2005
HA (H3)	219~H3N8~BCO_2005	CY047504	A/mallard/British Columbia/07569/2005
HA (H3)	22~H3N5~ALB_1983	CY005941	A/mallard/Alberta/635/1983
HA (H3)	220~H3N8~ALB_2005	CY047512	A/mallard/Alberta/11527/2005
HA (H3)	221~H3N8~ALB_2005	CY047520	A/blue-winged teal/Alberta/11646/2005
HA (H3)	222~H3N8~ALB_2005	CY047528	A/northern pintail/Alberta/11701/2005
HA (H3)	223~H3N2~QUB_2006	CY047592	A/mallard/Quebec/11040/2006
HA (H3)	224~H3N2~QUB_2006	CY047600	A/mallard/Quebec/11045/2006
HA (H3)	225~H3N8~QUB_2006	CY047608	A/mallard/Quebec/11082/2006
HA (H3)	226~H3N2~QUB_2006	CY047632	A/mallard/Quebec/11121/2006
HA (H3)	227~H3N2~QUB_2006	CY047648	A/American black duck/Quebec/11235/2006
HA (H3)	228~H3N2~QUB_2006	CY047656	A/mallard/Quebec/11247/2006
HA (H3)	229~H3N2~QUB_2006	CY047672	A/mallard/Quebec/11194/2006
HA (H3)	23~H3N3~ALB_1988	CY005942	A/mallard/ALB/394/1988
HA (H3)	230~H3N6~NEB_2007	CY047696	A/American black duck/New Brunswick/25182/2007
HA (H3)	231~H3N8~OH_2002	CY053829	A/mallard/Ohio/651/2002
HA (H3)	232~H3N8~OH_2002	CY053837	A/green-winged teal/Ohio/960/2002
HA (H3)	233~H3N6~OH_2006	CY053861	A/mallard/Ohio/1506/2006

HA (H3)	235~H3N8~AK_2006	EU557492	A/northern pintail/Alaska/44155-158/2006
HA (H3)	236~H3N8~AK_2006	EU557493	A/northern pintail/Alaska/44160-044/2006
HA (H3)	237~H3N8~AK_2006	EU557494	A/northern pintail/Alaska/44183-067/2006
HA (H3)	238~H3N8~AK_2006	EU557495	A/northern pintail/Alaska/44183-072/2006
HA (H3)	239~H3N6~AK_2006	EU557496	A/northern pintail/Alaska/44187-100/2006
HA (H3)	24~H3N1~ALB_1976	CY005943	A/mallard duck/ALB/26/1976
HA (H3)	240~H3N6~AK_2006	EU557497	A/northern pintail/Alaska/44194-041/2006
HA (H3)	241~H3N6~AK_2006	EU557498	A/northern pintail/Alaska/44194-071/2006
HA (H3)	242~H3N8~AK_2006	EU557499	A/northern pintail/Alaska/44182-129/2006
HA (H3)	243~H3N8~AK_2006	EU557500	A/northern pintail/Alaska/44184-003/2006
HA (H3)	244~H3N8~AK_2006	EU557501	A/northern pintail/Alaska/44244-108/2006
HA (H3)	245~H3N8~AK_2006	EU557502	A/northern pintail/Alaska/44228-129/2006
HA (H3)	246~H3N8~AK_2006	EU557503	A/northern pintail/Alaska/44228-173/2006
HA (H3)	247~H3N8~AK_2006	EU557504	A/northern pintail/Alaska/44228-175/2006
HA (H3)	248~H3N8~AK_2006	EU557505	A/northern pintail/Alaska/44202-126/2006
HA (H3)	249~H3N8~AK_2006	EU557506	A/northern pintail/Alaska/44204-075/2006
HA (H3)	25~H3N8~ALB_1980	CY005977	A/pintail duck/Alberta/169/1980
HA (H3)	250~H3N1~AK_2006	EU557507	A/northern pintail/Alaska/44204-108/2006
HA (H3)	251~H3N8~AK_2006	EU557508	A/northern pintail/Alaska/44202-103/2006
HA (H3)	252~H3N2~MN_1999	EU743495	A/mallard/Minnesota/68/1999
HA (H3)	253~H3N2~MN_1999	EU743501	A/mallard/Minnesota/290/1999
HA (H3)	254~H3N2~MN_1999	EU743508	A/mallard/Minnesota/380/1999
HA (H3)	255~H3N1~MN_1999	EU743516	A/mallard/MN/330/1999
HA (H3)	256~H3N1~MN_1999	EU743524	A/mallard/MN/351/1999
HA (H3)	257~H3N5~MN_1999	EU743530	A/mallard/Minnesota/280/1999
HA (H3)	258~H3N6~MN_1999	EU743536	A/mallard/MN/259/1999
HA (H3)	259~H3N6~MN_2000	EU743544	A/mallard/MN/99/2000
HA (H3)	26~H3N8~OH_2002	CY011028	A/blue-winged teal/Ohio/926/2002
HA (H3)	260~H3N6~MN_2000	EU743552	A/mallard/MN/515/2000
HA (H3)	261~H3N6~MN_1999	EU743560	A/pintail/Minnesota/479/1999
HA (H3)	262~H3N6~NJ_2005	EU871844	A/ruddy turnstone/NJ/335/2005
HA (H3)	263~H3N6~NJ_2005	EU871852	A/ruddy turnstone/NJ/357/2005
HA (H3)	264~H3N6~NJ_2005	EU871860	A/sanderling/NJ/1042/2005
HA (H3)	265~H3N9~MN_2000	EU871868	A/mallard/MN/158/2000
HA (H3)	266~H3N8~MN_1999	FJ517282	A/mallard/Minnesota/190/1999
HA (H3)	267~H3N8~MN_1999	FJ517289	A/mallard/Minnesota/283/1999
HA (H3)	268~H3N8~MN_1999	FJ517296	A/mallard/Minnesota/354/1999
HA (H3)	269~H3N8~ALB_2006	FJ877137	A/mallard/AB/431/2006
HA (H3)	27~H3N2~OH_2002	CY011048	A/blue-winged teal/Ohio/908/2002
HA (H3)	270~H3N8~AK_2006	GQ168610	A/Pacific golden plover/Alaska/44201-109/2006
HA (H3)	271~H3N1~MN_2000	GU051128	A/mallard/Minnesota/417/2000
HA (H3)	272~H3N2~NC_2004	GU051135	A/American black duck/North Carolina/675-075/2004
HA (H3)	273~H3N2~MN_2000	GU051140	A/mallard/Minnesota/282/2000
HA (H3)	274~H3N8~MN_2000	GU051147	A/mallard/Minnesota/448/2000
HA (H3)	275~H3N8~WA_1978	GU052299	A/American black duck/Washington/699/1978
HA (H3)	276~H3N8~NJ_2005	GU186466	A/laughing gull/NJ/768/2005
HA (H3)	277~H3N8~AK_2006	HM060000	A/glaucous gull/Alaska/44201-161/2006
HA (H3)	28~H3N6~AK_2005	CY013263	A/pintail/Alaska/53/2005
HA (H3)	29~H3N6~ALB_1985	CY014548	A/mallard duck/Alberta/331/1985
HA (H3)	3~H3N6~ALB_1999	AY633172	A/mallard/Alberta/199/1999
HA (H3)	30~H3N8~ALB_1991	CY014571	A/blue-winged teal/Alberta/120/1991
HA (H3)	31~H3N6~NY_1986	CY014865	A/mallard duck/New York/157/1986
HA (H3)	32~H3N8~NY_1982	CY014961	A/mallard duck/New York/174/1982
HA (H3)	33~H3N8~AK_2005	CY015492	A/mallard/Alaska/256/2005
HA (H3)	34~H3N8~AK_2005	CY015500	A/pintail/Alaska/279/2005
HA (H3)	35~H3N8~OH_1987	CY016140	A/pintail/Ohio/454/1987
HA (H3)	36~H3N8~AK_2005	CY016180	A/pintail/Alaska/211/2005
HA (H3)	37~H3N8~OH_1986	CY016395	A/mallard/Ohio/264/1986
HA (H3)	38~H3N8~AK_2005	CY016411	A/mallard/Alaska/715/2005
HA (H3)	39~H3N2~OH_1988	CY017411	A/mallard/Ohio/424/1988
HA (H3)	4~H3N8~ALB_1998	AY633244	A/mallard/Alberta/242/98
HA (H3)	40~H3N6~OH_1990	CY017709	A/mallard/Ohio/156/1990
HA (H3)	41~H3N8~AK_2005	CY017757	A/pintail/Alaska/779/2005
HA (H3)	42~H3N8~MD_2005	CY017773	A/longtail duck/Maryland/291/2005
HA (H3)	43~H3N8~OH_1987	CY017837	A/green-winged teal/Ohio/466/1987
HA (H3)	44~H3N8~OH_1987	CY017861	A/northern shoveler/Ohio/454/1987
HA (H3)	45~H3N2~OH_1999	CY017999	A/blue-winged teal/Ohio/31/1999
HA (H3)	46~H3N8~OH_1987	CY019197	A/pintail/Ohio/339/1987
HA (H3)	47~H3N2~OH_1986	CY020717	A/mallard/Ohio/48/1986
HA (H3)	48~H3N8~OH_2002	CY020741	A/mallard/Ohio/654/2002
HA (H3)	49~H3N8~OH_2002	CY020757	A/mallard/Ohio/649/2002
HA (H3)	5~H3N8~ALB_1998	AY633252	A/mallard/Alberta/279/98
HA (H3)	50~H3N8~AK_2005	CY020877	A/pintail/Alaska/49/2005
HA (H3)	51~H3N8~OH_1986	CY020933	A/northern shoveler/Ohio/35/1986

HA (H3)	52~H3N2~MD_2005	CY021261	A/mallard/Maryland/681/2005
HA (H3)	53~H3N2~MD_2005	CY021269	A/mallard/Maryland/691/2005
HA (H3)	54~H3N2~MD_2005	CY021277	A/mallard/Maryland/631/2005
HA (H3)	55~H3N8~OH_2005	CY021317	A/green-winged teal/Ohio/1289/2005
HA (H3)	56~H3N8~OH_2005	CY021341	A/mallard/Ohio/1801/2005
HA (H3)	57~H3N1~OH_1986	CY021429	A/mallard/Ohio/181/1986
HA (H3)	58~H3N2~MD_2005	CY021453	A/mallard/Maryland/615/2005
HA (H3)	59~H3N2~MD_2005	CY021461	A/mallard/Maryland/710/2005
HA (H3)	6~H3N8~ALB_1997	AY633340	A/pintail/Alberta/156/97
HA (H3)	60~H3N6~OH_2005	CY022717	A/mallard/Ohio/1717/2005
HA (H3)	61~H3N2~MD_2005	CY022741	A/mallard/Maryland/712/2005
HA (H3)	62~H3N2~MD_2005	CY024810	A/mallard/Maryland/708/2005
HA (H3)	63~H3N6~MD_2006	CY028692	A/mallard/Maryland/1235/2006
HA (H3)	64~H3N8~OH_2006	CY029929	A/blue-winged teal/Ohio/1864/2006
HA (H3)	65~H3N5~CA_2007	CY032672	A/northern shoveler/California/HKWF1201/2007
HA (H3)	67~H3N3~CA_2007	CY032728	A/northern shoveler/California/HKWF979/2007
HA (H3)	68~H3N7~CA_2007	CY033324	A/northern shoveler/California/HKWF1021/2007
HA (H3)	69~H3N5~CA_2007	CY033332	A/northern shoveler/California/HKWF1046/2007
HA (H3)	7~H3N8~ALB_1999	AY633372	A/pintail/Alberta/37/99
HA (H3)	70~H3N5~CA_2007	CY033348	A/northern shoveler/California/HKWF1131/2007
HA (H3)	71~H3N5~CA_2007	CY033364	A/northern shoveler/California/HKWF1199/2007
HA (H3)	72~H3N7~CA_2007	CY033372	A/northern shoveler/California/HKWF1367/2007
HA (H3)	73~H3N8~MN_2007	CY033691	A/mallard duck/Minnesota/Sg-00048/2007
HA (H3)	74~H3N8~MN_2007	CY033693	A/mallard duck/Minnesota/Sg-00051/2007
HA (H3)	75~H3N8~MN_2007	CY033703	A/mallard duck/Minnesota/Sg-00059/2007
HA (H3)	76~H3N8~MN_2007	CY033707	A/mallard duck/Minnesota/Sg-00064/2007
HA (H3)	77~H3N8~MN_2007	CY033712	A/mallard duck/Minnesota/Sg-00072/2007
HA (H3)	78~H3N8~TX_2007	CY033720	A/blue-winged teal/Texas/Sg-00079/2007
HA (H3)	79~H3N6~TX_2007	CY033727	A/blue-winged teal/Texas/Sg-00083/2007
HA (H3)	8~H3N4~ALB_1985	CY004657	A/mallard duck/Alberta/438/1985
HA (H3)	80~H3N8~TX_2007	CY033731	A/blue-winged teal/Texas/Sg-00084/2007
HA (H3)	81~H3N6~TX_2007	CY033735	A/blue-winged teal/Texas/Sg-00085/2007
HA (H3)	82~H3N3~MN_2007	CY033756	A/mallard duck/Minnesota/Sg-00100/2007
HA (H3)	83~H3N8~MN_2007	CY033760	A/mallard duck/Minnesota/Sg-00103/2007
HA (H3)	84~H3N6~MN_2007	CY033771	A/mallard duck/Minnesota/Sg-00116/2007
HA (H3)	85~H3N8~MN_2007	CY033779	A/mallard duck/Minnesota/Sg-00119/2007
HA (H3)	86~H3N7~CA_2007	CY034186	A/northern shoveler/California/HKWF848/2007
HA (H3)	87~H3N8~MN_2007	CY034626	A/blue-winged teal/Minnesota/Sg-00037/2007
HA (H3)	88~H3N8~MN_2007	CY034630	A/mallard duck/Minnesota/Sg-00047/2007
HA (H3)	89~H3N8~MN_2007	CY034639	A/mallard duck/Minnesota/Sg-00061/2007
HA (H3)	9~H3N8~ALB_1985	CY004662	A/mallard duck/ALB/525/1985
HA (H3)	90~H3N8~MN_2007	CY034642	A/ring-necked duck/Minnesota/Sg-00069/2007
HA (H3)	91~H3N6~MN_2007	CY034661	A/mallard duck/Minnesota/Sg-00098/2007
HA (H3)	93~H3N6~MN_2007	CY035299	A/mallard/Minnesota/Sg-00174/2007
HA (H3)	94~H3N8~MN_2007	CY035317	A/mallard/Minnesota/Sg-00184/2007
HA (H3)	95~H3N8~MN_2007	CY035334	A/mallard/Minnesota/Sg-00192/2007
HA (H3)	96~H3N8~MN_2007	CY035352	A/mallard/Minnesota/Sg-00202/2007
HA (H3)	97~H3N8~MN_2007	CY035356	A/mallard/Minnesota/Sg-00203/2007
HA (H3)	98~H3N8~TX_2007	CY035364	A/blue-winged teal/Texas/Sg-00205/2007
HA (H3)	99~H3N8~MN_2007	CY035379	A/mallard/Minnesota/Sg-00211/2007
HA (H4)	1~H4N2~ALB_1979	AB292406	A/mallard/Alberta/223/1979
HA (H4)	10~H4N1~ALB_1998	CY004925	A/mallard/Alberta/47/98
HA (H4)	100~H4N7~DE_2008	CY038041	A/ruddy turnstone/Delaware/Sg-00474/2008
HA (H4)	101~H4N6~NJ_2008	CY038051	A/ruddy turnstone/New Jersey/Sg-00477/2008
HA (H4)	102~H4N6~NJ_2008	CY038061	A/red knot/New Jersey/Sg-00479/2008
HA (H4)	103~H4N6~NJ_2008	CY038066	A/ruddy turnstone/New Jersey/Sg-00480/2008
HA (H4)	104~H4N6~NJ_2008	CY038091	A/ruddy turnstone/New Jersey/Sg-00489/2008
HA (H4)	105~H4N6~NJ_2008	CY038106	A/ruddy turnstone/New Jersey/Sg-00494/2008
HA (H4)	106~H4N6~NJ_2008	CY038116	A/ruddy turnstone/New Jersey/Sg-00496/2008
HA (H4)	107~H4N6~NJ_2008	CY038141	A/ruddy turnstone/New Jersey/Sg-00506/2008
HA (H4)	108~H4N6~NJ_2008	CY038146	A/ruddy turnstone/New Jersey/Sg-00507/2008
HA (H4)	109~H4N6~NJ_2008	CY038181	A/ruddy turnstone/New Jersey/Sg-00527/2008
HA (H4)	11~H4N8~ALB_1999	CY004933	A/pintail/Alberta/207/1999
HA (H4)	110~H4N6~NJ_2008	CY038186	A/ruddy turnstone/New Jersey/Sg-00533/2008
HA (H4)	111~H4N6~NJ_2008	CY038191	A/ruddy turnstone/New Jersey/Sg-00534/2008
HA (H4)	112~H4N6~DE_2008	CY038196	A/ruddy turnstone/Delaware/Sg-00539/2008
HA (H4)	113~H4N6~NJ_2008	CY038201	A/ruddy turnstone/New Jersey/Sg-00542/2008
HA (H4)	114~H4N6~NJ_2008	CY038206	A/ruddy turnstone/New Jersey/Sg-00550/2008
HA (H4)	115~H4N6~NJ_2008	CY038216	A/ruddy turnstone/New Jersey/Sg-00552/2008
HA (H4)	116~H4N6~NJ_2008	CY038221	A/ruddy turnstone/New Jersey/Sg-00556/2008
HA (H4)	117~H4N6~NJ_2008	CY038226	A/ruddy turnstone/New Jersey/Sg-00558/2008
HA (H4)	118~H4N6~NJ_2008	CY038251	A/ruddy turnstone/New Jersey/Sg-00565/2008
HA (H4)	119~H4N6~MN_2008	CY038256	A/mallard/Minnesota/Sg-00569/2008
HA (H4)	12~H4N8~ALB_2001	CY004939	A/mallard/Alberta/30/2001

HA (H4)	120~H4N8~AK_2007	CY038370	A/northern pintail/Interior Alaska/2/2007
HA (H4)	121~H4N6~CA_2008	CY039739	A/mallard/California/7766/2008
HA (H4)	122~H4N6~AK_2007	CY039771	A/mallard/Interior Alaska/6/2007
HA (H4)	123~H4N6~AK_2007	CY039786	A/mallard/Interior Alaska/4/2007
HA (H4)	124~H4N6~AK_2007	CY039810	A/mallard/Interior Alaska/2/2007
HA (H4)	125~H4N6~AK_2007	CY039834	A/northern shoveler/Interior Alaska/1/2007
HA (H4)	126~H4N5~AK_2007	CY039842	A/mallard/Interior Alaska/1/2007
HA (H4)	127~H4N6~AK_2007	CY039866	A/mallard/Interior Alaska/3/2007
HA (H4)	128~H4N6~MN_2007	CY041895	A/mallard/Minnesota/Sg-00133/2007
HA (H4)	129~H4N6~MN_2007	CY042016	A/mallard duck/Minnesota/Sg-00054/2007
HA (H4)	13~H4N6~ALB_1977	CY005944	A/canvasback duck/ALB/274/1977
HA (H4)	130~H4N8~MN_2007	CY042034	A/mallard duck/Minnesota/Sg-00070/2007
HA (H4)	131~H4N6~TX_2007	CY042096	A/blue-winged teal/Texas/Sg-00124/2007
HA (H4)	132~H4N6~MN_2006	CY042239	A/blue-winged teal/Minnesota/Sg-00229/2006
HA (H4)	133~H4N6~MN_2007	CY042249	A/American wigeon/Minnesota/Sg-00231/2007
HA (H4)	134~H4N8~MN_2008	CY042340	A/blue-winged teal/Minnesota/Sg-00450/2008
HA (H4)	135~H4N2~SD_2008	CY042368	A/mallard/South Dakota/Sg-00457/2008
HA (H4)	136~H4N6~SD_2008	CY042372	A/mallard/South Dakota/Sg-00458/2008
HA (H4)	137~H4N6~SD_2008	CY042376	A/mallard/South Dakota/Sg-00459/2008
HA (H4)	138~H4N6~SD_2008	CY042380	A/mallard/South Dakota/Sg-00460/2008
HA (H4)	139~H4N8~MN_2008	CY042399	A/mallard/Minnesota/Sg-00465/2008
HA (H4)	14~H4N6~ALB_1977	CY005945	A/gadwall duck/ALB/53/1977
HA (H4)	140~H4N6~NJ_2008	CY042506	A/ruddy turnstone/New Jersey/Sg-00524/2008
HA (H4)	141~H4N6~NJ_2008	CY042522	A/ruddy turnstone/New Jersey/Sg-00531/2008
HA (H4)	142~H4N6~DE_2008	CY042549	A/ruddy turnstone/Delaware/Sg-00541/2008
HA (H4)	143~H4N8~MN_2008	CY042643	A/mallard/Minnesota/Sg-00624/2008
HA (H4)	144~H4N8~MN_2008	CY042648	A/mallard/Minnesota/Sg-00625/2008
HA (H4)	145~H4N8~MN_2008	CY042653	A/mallard/Minnesota/Sg-00626/2008
HA (H4)	146~H4N8~MN_2008	CY042675	A/mallard/Minnesota/Sg-00631/2008
HA (H4)	147~H4N8~MN_2008	CY042693	A/mallard/Minnesota/Sg-00635/2008
HA (H4)	148~H4N6~MN_2008	CY042744	A/northern shoveler/Minnesota/Sg-00647/2008
HA (H4)	149~H4N8~MN_2008	CY042910	A/blue-winged teal/Minnesota/Sg-00691/2008
HA (H4)	15~H4N6~ALB_1977	CY005946	A/redhead duck/ALB/74/1977
HA (H4)	150~H4N6~MN_2008	CY042918	A/mallard/Minnesota/Sg-00693/2008
HA (H4)	151~H4N6~MN_2008	CY042922	A/mallard/Minnesota/Sg-00694/2008
HA (H4)	152~H4N6~MN_2008	CY042926	A/mallard/Minnesota/Sg-00695/2008
HA (H4)	153~H4N6~MN_2008	CY042934	A/mallard/Minnesota/Sg-00697/2008
HA (H4)	154~H4N8~MN_2008	CY042938	A/mallard/Minnesota/Sg-00698/2008
HA (H4)	155~H4N8~MN_2008	CY042942	A/mallard/Minnesota/Sg-00700/2008
HA (H4)	156~H4N6~ND_2008	CY042950	A/blue-winged teal/North Dakota/Sg-00702/2008
HA (H4)	157~H4N6~ND_2008	CY042962	A/blue-winged teal/North Dakota/Sg-00705/2008
HA (H4)	158~H4N6~ND_2008	CY042970	A/blue-winged teal/North Dakota/Sg-00708/2008
HA (H4)	159~H4N8~ND_2008	CY042974	A/blue-winged teal/North Dakota/Sg-00711/2008
HA (H4)	16~H4N6~ALB_1977	CY005947	A/blue-winged teal/ALB/243/1977
HA (H4)	160~H4N6~ND_2008	CY042978	A/blue-winged teal/North Dakota/Sg-00712/2008
HA (H4)	161~H4N6~ND_2008	CY042982	A/blue-winged teal/North Dakota/Sg-00713/2008
HA (H4)	162~H4N6~ND_2008	CY042990	A/blue-winged teal/North Dakota/Sg-00715/2008
HA (H4)	163~H4N6~ND_2008	CY042994	A/blue-winged teal/North Dakota/Sg-00716/2008
HA (H4)	164~H4N6~ND_2008	CY042998	A/blue-winged teal/North Dakota/Sg-00717/2008
HA (H4)	165~H4N6~ND_2008	CY043005	A/blue-winged teal/North Dakota/Sg-00724/2008
HA (H4)	166~H4N6~ND_2008	CY043008	A/blue-winged teal/North Dakota/Sg-00727/2008
HA (H4)	167~H4N6~ND_2008	CY043011	A/blue-winged teal/North Dakota/Sg-00731/2008
HA (H4)	168~H4N6~ND_2008	CY043012	A/blue-winged teal/North Dakota/Sg-00732/2008
HA (H4)	169~H4N6~ND_2008	CY043014	A/blue-winged teal/North Dakota/Sg-00734/2008
HA (H4)	17~H4N3~ALB_1977	CY005948	A/mallard/Alberta/300/1977
HA (H4)	170~H4N8~ND_2008	CY043018	A/blue-winged teal/North Dakota/Sg-00741/2008
HA (H4)	171~H4N6~ND_2008	CY043020	A/blue-winged teal/North Dakota/Sg-00743/2008
HA (H4)	172~H4N6~ND_2008	CY043021	A/American wigeon/North Dakota/Sg-00744/2008
HA (H4)	173~H4N6~ND_2008	CY043026	A/blue-winged teal/North Dakota/Sg-00750/2008
HA (H4)	174~H4N6~ND_2008	CY043028	A/blue-winged teal/North Dakota/Sg-00752/2008
HA (H4)	175~H4N6~ND_2008	CY043029	A/blue-winged teal/North Dakota/Sg-00753/2008
HA (H4)	176~H4N6~ND_2008	CY043030	A/blue-winged teal/North Dakota/Sg-00754/2008
HA (H4)	177~H4N6~ND_2008	CY043031	A/blue-winged teal/North Dakota/Sg-00756/2008
HA (H4)	178~H4N6~NJ_2008	CY043035	A/mallard/New Jersey/Sg-00760/2008
HA (H4)	179~H4N6~NJ_2008	CY043037	A/mallard/New Jersey/Sg-00762/2008
HA (H4)	18~H4N8~ALB_1977	CY005950	A/mallard duck/ALB/210/1977
HA (H4)	180~H4N8~MN_2008	CY043038	A/mallard/Minnesota/Sg-00763/2008
HA (H4)	181~H4N8~MN_2008	CY043041	A/mallard/Minnesota/Sg-00767/2008
HA (H4)	182~H4N6~MN_2008	CY043043	A/mallard/Minnesota/Sg-00769/2008
HA (H4)	183~H4N6~MN_2008	CY043046	A/mallard/Minnesota/Sg-00774/2008
HA (H4)	184~H4N6~MN_2008	CY043057	A/mallard/Minnesota/Sg-00793/2008
HA (H4)	185~H4N6~MN_2008	CY043058	A/mallard/Minnesota/Sg-00794/2008
HA (H4)	186~H4N2~MN_2008	CY043062	A/blue-winged teal/Minnesota/Sg-00798/2008
HA (H4)	187~H4N6~MN_2008	CY043063	A/blue-winged teal/Minnesota/Sg-00799/2008

HA (H4)	188~H4N6~MN_2008	CY043064	A/blue-winged teal/Minnesota/Sg-00800/2008
HA (H4)	189~H4N6~MN_2008	CY043071	A/mallard/Minnesota/Sg-00807/2008
HA (H4)	19~H4N6~ALB_1977	CY005951	A/mallard duck/ALB/161/1977
HA (H4)	190~H4N6~MN_2008	CY043073	A/mallard/Minnesota/Sg-00809/2008
HA (H4)	191~H4N6~AK_2007	CY043952	A/northern shoveler/Alaska/7MP1113/2007
HA (H4)	192~H4N8~AK_2007	CY043968	A/mallard/Alaska/7MP1028/2007
HA (H4)	193~H4N6~AK_2007	CY043992	A/northern pintail/Alaska/7MP1393/2007
HA (H4)	194~H4N8~AK_2007	CY044000	A/least sandpiper/Alaska/7KW0411/2007
HA (H4)	195~H4N6~AK_2007	CY044016	A/northern shoveler/Alaska/7MP0954/2007
HA (H4)	196~H4N6~NBR_2006	CY045303	A/American black duck/New Brunswick/19347/2006
HA (H4)	197~H4N6~NBR_2006	CY045311	A/American black duck/New Brunswick/19502/2006
HA (H4)	198~H4N6~CA_2005	CY045335	A/mallard/California/GL30/2005
HA (H4)	199~H4N6~CA_2005	CY045351	A/green-winged teal/California/K218/2005
HA (H4)	2~H4N6~ALB_1999	AY633124	A/mallard/Alberta/111/99
HA (H4)	20~H4N8~ALB_1977	CY005952	A/pintail duck/ALB/220/1977
HA (H4)	200~H4N6~AK_2007	CY045415	A/mallard/Interior Alaska/7MP0372/2007
HA (H4)	201~H4N6~AK_2007	CY045423	A/mallard/Interior Alaska/7MP1050R1/2007
HA (H4)	202~H4N6~AK_2007	CY045455	A/northern shoveler/Interior Alaska/7MP1601/2007
HA (H4)	203~H4N5~AK_2007	CY045463	A/mallard/Interior Alaska/7MP1718/2007
HA (H4)	204~H4N6~ALB_2005	CY047536	A/green-winged teal/Alberta/11383/2005
HA (H4)	205~H4N6~ALB_2005	CY047544	A/redhead/Alberta/11817/2005
HA (H4)	206~H4N6~NSC_2005	CY047568	A/green-winged teal/Nova Scotia/14687/2005
HA (H4)	207~H4N6~QBC_2006	CY047576	A/mallard/Quebec/11102/2006
HA (H4)	208~H4N6~QBC_2006	CY047584	A/mallard/Quebec/11002/2006
HA (H4)	209~H4N6~QBC_2006	CY047616	A/mallard/Quebec/11103/2006
HA (H4)	21~H4N2~ALB_1978	CY005953	A/mallard duck/Alberta/354/1978
HA (H4)	210~H4N6~QBC_2006	CY047624	A/mallard/Quebec/11106/2006
HA (H4)	211~H4N6~QBC_2006	CY047640	A/mallard/Quebec/11182/2006
HA (H4)	212~H4N6~MAN_2007	CY047680	A/American green-winged teal/Manitoba/23884/2007
HA (H4)	213~H4N7~MAN_2007	CY047688	A/mallard/Manitoba/23912/2007
HA (H4)	214~H4N6~OH_2002	CY053845	A/mallard/Ohio/660/2002
HA (H4)	215~H4N6~AK_2006	EU557509	A/northern pintail/Alaska/44161-173/2006
HA (H4)	216~H4N6~AK_2006	EU557510	A/northern pintail/Alaska/44191-123/2006
HA (H4)	217~H4N6~AK_2006	EU557511	A/northern pintail/Alaska/44188-158/2006
HA (H4)	218~H4N6~AK_2006	EU557512	A/northern pintail/Alaska/44188-175/2006
HA (H4)	219~H4N6~AK_2006	EU557513	A/northern pintail/Alaska/44189-069/2006
HA (H4)	22~H4N2~ALB_1979	CY005954	A/mallard duck/Alberta/106/1979
HA (H4)	220~H4N6~AK_2006	EU557514	A/northern pintail/Alaska/44243-063/2006
HA (H4)	221~H4N6~AK_2006	EU557515	A/northern pintail/Alaska/44203-079/2006
HA (H4)	222~H4N6~MN_1999	EU871876	A/mallard/Minnesota/160/1999
HA (H4)	223~H4N6~MN_1999	EU871884	A/mallard/Minnesota/269/1999
HA (H4)	224~H4N8~MN_1999	EU871892	A/mallard/MN/323/1999
HA (H4)	225~H4N8~MN_1999	FJ517314	A/mallard/MN/327/1999
HA (H4)	226~H4N6~CA_2006	FJ520092	A/northern pintail/California/44249-38/2006
HA (H4)	227~H4N2~CA_2007	FJ520103	A/northern pintail/California/44345-762/2007
HA (H4)	228~H4N6~CA_2007	FJ520105	A/northern pintail/California/44355-442/2007
HA (H4)	229~H4N6~QBC_2006	FJ877136	A/mallard/QC/147/2006
HA (H4)	23~H4N2~ALB_1979	CY005955	A/blue-winged teal/Alberta/580/1979
HA (H4)	230~H4N8~MN_1999	GU051281	A/mallard/Minnesota/271/1999
HA (H4)	24~H4N2~ALB_1979	CY005956	A/pintail duck/ALB/599/1979
HA (H4)	25~H4N4~ALB_1983	CY005957	A/mallard duck/Alberta/581/1983
HA (H4)	27~H4N2~ALB_1984	CY005959	A/mallard duck/Alberta/630/1984
HA (H4)	28~H4N6~ALB_1985	CY005961	A/pintail duck/ALB/623/1985
HA (H4)	29~H4N6~DE_1988	CY005962	A/ruddy turnstone/DE/512/1988
HA (H4)	3~H4N6~ALB_2000	AY633141	A/mallard/Alberta/119/00
HA (H4)	30~H4N5~ALB_1990	CY005963	A/blue-winged teal/ALB/103/1990
HA (H4)	31~H4N3~ALB_1990	CY005964	A/blue-winged teal/ALB/136/1990
HA (H4)	32~H4N6~ALB_1994	CY005965	A/blue-winged teal/ALB/293/1994
HA (H4)	33~H4N6~ALB_2001	CY005966	A/pintail/Alberta/269/2001
HA (H4)	34~H4N6~ALB_2003	CY005967	A/blue-winged teal/Alberta/293/2003
HA (H4)	35~H4N1~ALB_1977	CY005968	A/mallard duck/Alberta/291/1977
HA (H4)	36~H4N6~OH_2002	CY011036	A/mallard/Ohio/657/2002
HA (H4)	37~H4N2~OH_1987	CY011056	A/mallard/Ohio/275/1987
HA (H4)	38~H4N6~OH_1987	CY012808	A/mallard/Ohio/298/1987
HA (H4)	39~H4N2~OH_1989	CY012816	A/mallard/Ohio/97/1989
HA (H4)	4~H4N6~ALB_2000	AY633156	A/mallard/Alberta/136/00
HA (H4)	40~H4N8~OH_1986	CY013248	A/mallard/Ohio/338/1986
HA (H4)	41~H4N6~ALB_2003	CY014562	A/mallard/Alberta/254/2003
HA (H4)	42~H4N4~ALB_1977	CY014579	A/mallard duck/Alberta/299/1977
HA (H4)	43~H4N9~NY_1986	CY014857	A/mallard duck/New York/180/1986
HA (H4)	44~H4N2~NY_1979	CY014922	A/blue-winged teal/New York/370ac/1979
HA (H4)	45~H4N8~NY_1982	CY014929	A/mallard duck/New York/194/1982
HA (H4)	46~H4N3~NY_1982	CY014937	A/pintail duck/New York/155/1982
HA (H4)	47~H4N2~OH_1986	CY015459	A/green-winged teal/Ohio/344/1986

HA (H4)	48~H4N6~OH_1986	CY015467	A/mallard/Ohio/83/1986
HA (H4)	49~H4N2~OH_1989	CY016148	A/mallard/Ohio/94/1989
HA (H4)	5~H4N6~ALB_1998	AY633260	A/mallard/Alberta/295/98
HA (H4)	50~H4N6~OH_1988	CY017701	A/mallard/Ohio/324/1988
HA (H4)	51~H4N6~AK_2005	CY017741	A/pintail/Alaska/310/2005
HA (H4)	52~H4N6~OH_2002	CY020725	A/mallard/Ohio/667/2002
HA (H4)	53~H4N6~MD_2002	CY020733	A/mallard/Maryland/750/2002
HA (H4)	54~H4N6~OH_2002	CY020749	A/blue-winged teal/Ohio/989/2002
HA (H4)	55~H4N8~MD_2002	CY020765	A/black duck/Maryland/834/2002
HA (H4)	56~H4N6~OH_2002	CY020773	A/mallard/Ohio/655/2002
HA (H4)	57~H4N6~OH_2002	CY020789	A/mallard/Ohio/668/2002
HA (H4)	58~H4N6~OH_2002	CY020797	A/mallard/Ohio/671/2002
HA (H4)	59~H4N2~OH_2002	CY020805	A/mallard/Ohio/686/2002
HA (H4)	6~H4N6~ALB_1998	AY633268	A/mallard/Alberta/30/98
HA (H4)	60~H4N6~OH_1989	CY020981	A/mallard/Ohio/129/1989
HA (H4)	61~H4N6~OH_1990	CY021213	A/mallard/Ohio/178/1990
HA (H4)	62~H4N6~OH_2005	CY021221	A/blue-winged teal/Ohio/1339/2005
HA (H4)	63~H4N6~OH_2005	CY021325	A/green-winged teal/Ohio/1324/2005
HA (H4)	64~H4N6~MD_2005	CY021333	A/mallard/Maryland/1241/2005
HA (H4)	65~H4N6~OH_2005	CY021349	A/green-winged teal/Ohio/1292/2005
HA (H4)	66~H4N6~MD_2005	CY021893	A/white-winged scoter/Maryland/301/2005
HA (H4)	67~H4N8~CA_2007	CY032892	A/bufflehead/California/HKWF205/2007
HA (H4)	68~H4N7~CA_2007	CY032908	A/American wigeon/California/HKWF450/2007
HA (H4)	69~H4N6~MN_2007	CY033667	A/blue-winged teal/Minnesota/Sg-00030/2007
HA (H4)	70~H4N6~MN_2007	CY033671	A/blue-winged teal/Minnesota/Sg-00032/2007
HA (H4)	71~H4N6~MN_2007	CY033675	A/blue-winged teal/Minnesota/Sg-00034/2007
HA (H4)	72~H4N6~MN_2007	CY033677	A/blue-winged teal/Minnesota/Sg-00035/2007
HA (H4)	73~H4N6~MN_2007	CY033681	A/blue-winged teal/Minnesota/Sg-00039/2007
HA (H4)	74~H4N6~MN_2007	CY033685	A/blue-winged teal/Minnesota/Sg-00042/2007
HA (H4)	75~H4N6~MN_2007	CY033695	A/mallard duck/Minnesota/Sg-00052/2007
HA (H4)	76~H4N6~MN_2007	CY033702	A/mallard duck/Minnesota/Sg-00058/2007
HA (H4)	77~H4N6~MN_2007	CY033706	A/mallard duck/Minnesota/Sg-00063/2007
HA (H4)	78~H4N6~MN_2007	CY033711	A/ring-necked duck/Minnesota/Sg-00067/2007
HA (H4)	79~H4N6~MN_2007	CY033754	A/mallard duck/Minnesota/Sg-00096/2007
HA (H4)	8~H4N6~ALB_1985	CY004847	A/blue-winged teal/ALB/562/1985
HA (H4)	80~H4N6~MN_2007	CY034623	A/blue-winged teal/Minnesota/Sg-00033/2007
HA (H4)	81~H4N6~MN_2007	CY034624	A/blue-winged teal/Minnesota/Sg-00036/2007
HA (H4)	82~H4N6~MN_2007	CY034638	A/mallard duck/Minnesota/Sg-00060/2007
HA (H4)	83~H4N6~TX_2007	CY034647	A/blue-winged teal/Texas/Sg-00077/2007
HA (H4)	84~H4N6~TX_2007	CY034649	A/blue-winged teal/Texas/Sg-00081/2007
HA (H4)	85~H4N6~TX_2007	CY035257	A/blue-winged teal/Texas/Sg-00157/2007
HA (H4)	86~H4N6~TX_2007	CY035261	A/blue-winged teal/Texas/Sg-00158/2007
HA (H4)	87~H4N8~TX_2007	CY035265	A/blue-winged teal/Texas/Sg-00159/2007
HA (H4)	88~H4N8~LA_2007	CY035273	A/blue-winged teal/Louisiana/Sg-00163/2007
HA (H4)	89~H4N6~MN_2007	CY035303	A/mallard/Minnesota/Sg-00176/2007
HA (H4)	9~H4N6~ALB_1995	CY004911	A/mallard/ALB/49/1995
HA (H4)	90~H4N8~TX_2007	CY035324	A/blue-winged teal/Texas/Sg-00188/2007
HA (H4)	91~H4N6~TX_2007	CY035360	A/blue-winged teal/Texas/Sg-00204/2007
HA (H4)	92~H4N6~TX_2007	CY035368	A/blue-winged teal/Texas/Sg-00206/2007
HA (H4)	93~H4N6~MN_2007	CY035375	A/mallard/Minnesota/Sg-00210/2007
HA (H4)	94~H4N5~TX_2007	CY035382	A/blue-winged teal/Texas/Sg-00212/2007
HA (H4)	95~H4N8~AK_2007	CY035783	A/least sandpiper/South Central Alaska/2/2007
HA (H4)	96~H4N8~AK_2007	CY035791	A/least sandpiper/South Central Alaska/3/2007
HA (H4)	97~H4N8~AK_2007	CY035869	A/least sandpiper/South Central Alaska/1/2007
HA (H4)	98~H4N6~AK_2007	CY036651	A/northern pintail/Interior Alaska/1/2007
HA (H4)	99~H4N6~DE_2008	CY038036	A/ruddy turnstone/Delaware/Sg-00470/2008
NP	1~H13N6_1980	CY005861	A/gull/Minnesota/945/1980
NP	10~H3N2_1999	EU743504	A/mallard/Minnesota/290/1999
NP	11~H3N2_1999	EU743511	A/mallard/Minnesota/380/1999
NP	12~H3N1_1999	EU743519	A/mallard/MN/330/1999
NP	13~H3N1_1999	EU743526	A/mallard/MN/351/1999
NP	14~H3N5_1999	EU743532	A/mallard/Minnesota/280/1999
NP	15~H3N6_1999	EU743539	A/mallard/MN/259/1999
NP	16~H3N6_2000	EU743547	A/mallard/MN/99/2000
NP	17~H3N6_2000	EU743555	A/mallard/MN/515/2000
NP	18~H3N6_1999	EU743563	A/pintail/Minnesota/479/1999
NP	19~H3N9_2000	EU871871	A/mallard/MN/158/2000
NP	2~H6N6_1980	CY005884	A/blue-winged teal/MN/993/1980
NP	20~H4N6_1999	EU871879	A/mallard/Minnesota/160/1999
NP	21~H4N6_1999	EU871887	A/mallard/Minnesota/269/1999
NP	22~H4N8_1999	EU871895	A/mallard/MN/323/1999
NP	23~H5N2_2000	EU871902	A/mallard/MN/113/2000
NP	24~H5N3_2000	EU871909	A/mallard/MN/479/2000
NP	25~H5N5_2000	EU871916	A/mallard/MN/105/2000

NP	26~H5N2_2000	FJ357072	A/mallard/MN/1/2000
NP	27~H2N3_1998	FJ357080	A/mallard/MN/14/1998
NP	28~H2N3_1998	FJ357087	A/mallard/MN/51/1998
NP	29~H2N3_2000	FJ517270	A/mallard/MN/506/2000
NP	3~H10N7_1979	CY014742	A/mallard duck/Minnesota/19/1979
NP	30~H3N8_1999	FJ517285	A/mallard/Minnesota/190/1999
NP	31~H3N8_1999	FJ517291	A/mallard/Minnesota/283/1999
NP	32~H3N8_1999	FJ517298	A/mallard/Minnesota/354/1999
NP	33~H4N8_1999	FJ517317	A/mallard/MN/327/1999
NP	34~H11N9_2000	GQ257376	A/mallard/Minnesota/249/2000
NP	35~H5N2_2006	GQ923144	A/waterfowl/Minnesota/459675/2006
NP	36~H5N2_2006	GQ923216	A/mallard/Minnesota/464334/2006
NP	37~H2N2_1998	GU050727	A/mallard/Minnesota/26/1998
NP	38~H2N2_1998	GU050733	A/mallard/Minnesota/27/1998
NP	39~H2N1_2000	GU050746	A/mallard/Minnesota/550/2000
NP	4~H3N2_2007	CY041888	A/green-winged teal/Minnesota/Sg-00131/2007
NP	40~H2N9_1998	GU050802	A/mallard/Minnesota/34/1998
NP	41~H2N9_2000	GU050809	A/mallard/Minnesota/31/2000
NP	42~H10N7_2000	GU050885	A/mallard/Minnesota/518/2000
NP	43~H3N1_2000	GU051130	A/mallard/Minnesota/417/2000
NP	44~H3N2_2000	GU051142	A/mallard/Minnesota/282/2000
NP	45~H3N8_2000	GU051150	A/mallard/Minnesota/448/2000
NP	46~H3N8_1999	GU051183	A/mallard/Minnesota/231/1999
NP	47~H3N9_1998	GU051203	A/mallard/Minnesota/182/1998
NP	48~H4N2_1998	GU051207	A/mallard/Minnesota/95/1998
NP	49~H4N3_2000	GU051213	A/mallard/Minnesota/14/2000
NP	5~H4N6_2007	CY041896	A/mallard/Minnesota/Sg-00133/2007
NP	50~H4N6_2000	GU051242	A/mallard/Minnesota/313/2000
NP	51~H4N6_2000	GU051248	A/mallard/Minnesota/524/2000
NP	52~H4N8_1998	GU051253	A/mallard/Minnesota/114/1998
NP	53~H4N8_1999	GU051259	A/mallard/Minnesota/168/1999
NP	54~H4N8_1999	GU051263	A/mallard/Minnesota/188/1999
NP	55~H4N8_1999	GU051268	A/mallard/Minnesota/210/1999
NP	56~H4N8_1999	GU051276	A/mallard/Minnesota/237/1999
NP	57~H4N8_1999	GU051283	A/mallard/Minnesota/271/1999
NP	58~H4N8_1999	GU051289	A/mallard/Minnesota/402/1999
NP	59~H5N1_2000	GU051304	A/mallard/Minnesota/24/2000
NP	6~H11N9_2000	EU743460	A/mallard/Minnesota/42/2000
NP	60~H5N2_2000	GU051310	A/mallard/Minnesota/166/2000
NP	61~H5N2_2000	GU051315	A/mallard/Minnesota/168/2000
NP	62~H5N2_2000	GU051319	A/mallard/Minnesota/283/2000
NP	63~H5N2_2000	GU051325	A/mallard/Minnesota/410/2000
NP	64~H5N3_2000	GU051334	A/mallard/Minnesota/382/2000
NP	65~H6N5_1998	GU051418	A/mallard/Minnesota/66/1998
NP	66~H6N5_1999	GU051427	A/mallard/Minnesota/329/1999
NP	67~H6N6_1998	GU051435	A/mallard/Minnesota/179/1998
NP	68~H6N8_1998	GU051440	A/mallard/Minnesota/29/1998
NP	69~H6N8_1998	GU051445	A/mallard/Minnesota/63/1998
NP	7~H11N9_2000	EU743468	A/mallard/Minnesota/107/2000
NP	70~H6N8_1998	GU051449	A/mallard/Minnesota/353/1998
NP	71~H6N8_1999	GU051454	A/mallard/Minnesota/173/1999
NP	72~H6N8_1999	GU051460	A/mallard/Minnesota/204/1999
NP	73~H7N3_1999	GU051496	A/pintail/Minnesota/423/1999
NP	8~H11N9_2000	EU743475	A/mallard/Minnesota/109/2000
NP	9~H3N2_1999	EU743497	A/mallard/Minnesota/68/1999
NA(N6)	1~H1N6~AK_1976	AY207535	A/murre/Alaska/305/1976
NA(N6)	10~H4N6~ALB_1999	AY633358	A/pintail/Alberta/210/99
NA(N6)	100~H4N6~AK_2007	CY039812	A/mallard/Interior Alaska/2/2007
NA(N6)	101~H4N6~AK_2007	CY039836	A/northern shoveler/Interior Alaska/1/2007
NA(N6)	102~H4N6~AK_2007	CY039860	A/mallard/Interior Alaska/5/2007
NA(N6)	103~H4N6~AK_2007	CY039868	A/mallard/Interior Alaska/3/2007
NA(N6)	104~H4N6~MN_2007	CY041897	A/mallard/Minnesota/Sg-00133/2007
NA(N6)	105~H4N6~MN_2007	CY041988	A/blue-winged teal/Minnesota/Sg-00028/2007
NA(N6)	106~H4N6~MN_2007	CY041989	A/blue-winged teal/Minnesota/Sg-00029/2007
NA(N6)	107~H4N6~MN_2007	CY041990	A/blue-winged teal/Minnesota/Sg-00031/2007
NA(N6)	108~H4N6~MN_2007	CY041991	A/blue-winged teal/Minnesota/Sg-00032/2007
NA(N6)	109~H4N6~MN_2007	CY041992	A/blue-winged teal/Minnesota/Sg-00033/2007
NA(N6)	11~H3N6~ALB_1979	CY004123	A/mallard duck/ALB/676/1979
NA(N6)	110~H4N6~MN_2007	CY041993	A/blue-winged teal/Minnesota/Sg-00034/2007
NA(N6)	111~H4N6~MN_2007	CY041994	A/blue-winged teal/Minnesota/Sg-00035/2007
NA(N6)	112~H4N6~MN_2007	CY041995	A/blue-winged teal/Minnesota/Sg-00036/2007
NA(N6)	113~H4N6~MN_2007	CY041998	A/blue-winged teal/Minnesota/Sg-00038/2007
NA(N6)	114~H4N6~MN_2007	CY041999	A/blue-winged teal/Minnesota/Sg-00039/2007
NA(N6)	115~H4N6~MN_2007	CY042000	A/blue-winged teal/Minnesota/Sg-00040/2007

NA(N6)	116~H4N6~MN_2007	CY042001	A/blue-winged teal/Minnesota/Sg-00041/2007
NA(N6)	117~H4N6~MN_2007	CY042002	A/blue-winged teal/Minnesota/Sg-00042/2007
NA(N6)	118~H4N6~MN_2007	CY042003	A/blue-winged teal/Minnesota/Sg-00043/2007
NA(N6)	119~H4N6~MN_2007	CY042005	A/mallard duck/Minnesota/Sg-00045/2007
NA(N6)	12~H6N6~ALB_1982	CY004148	A/pintail/Alberta/189/82
NA(N6)	120~H4N6~MN_2007	CY042006	A/mallard duck/Minnesota/Sg-00046/2007
NA(N6)	121~H4N6~MN_2007	CY042009	A/mallard duck/Minnesota/Sg-00049/2007
NA(N6)	122~H4N6~MN_2007	CY042010	A/mallard duck/Minnesota/Sg-00050/2007
NA(N6)	123~H4N6~MN_2007	CY042012	A/mallard duck/Minnesota/Sg-00052/2007
NA(N6)	124~H4N6~MN_2007	CY042013	A/mallard duck/Minnesota/Sg-00053/2007
NA(N6)	125~H4N6~MN_2007	CY042017	A/mallard duck/Minnesota/Sg-00054/2007
NA(N6)	126~H4N6~MN_2007	CY042021	A/mallard duck/Minnesota/Sg-00058/2007
NA(N6)	127~H4N6~MN_2007	CY042023	A/mallard duck/Minnesota/Sg-00060/2007
NA(N6)	128~H4N6~MN_2007	CY042027	A/mallard duck/Minnesota/Sg-00063/2007
NA(N6)	129~H4N6~MN_2007	CY042031	A/ring-necked duck/Minnesota/Sg-00067/2007
NA(N6)	13~H6N6~ALB_1982	CY004156	A/widgeon/ALB/256/1982
NA(N6)	130~H4N6~TX_2007	CY042040	A/blue-winged teal/Texas/Sg-00075/2007
NA(N6)	131~H4N6~TX_2007	CY042041	A/blue-winged teal/Texas/Sg-00076/2007
NA(N6)	132~H4N6~TX_2007	CY042042	A/blue-winged teal/Texas/Sg-00077/2007
NA(N6)	133~H4N6~TX_2007	CY042046	A/blue-winged teal/Texas/Sg-00080/2007
NA(N6)	134~H3N6~TX_2007	CY042048	A/blue-winged teal/Texas/Sg-00083/2007
NA(N6)	135~H4N6~TX_2007	CY042052	A/cinnamon teal/Texas/Sg-00088/2007
NA(N6)	136~H4N6~TX_2007	CY042053	A/blue-winged teal/Texas/Sg-00089/2007
NA(N6)	137~H4N6~MN_2007	CY042061	A/blue-winged teal/Minnesota/Sg-00095/2007
NA(N6)	138~H4N6~MN_2007	CY042062	A/mallard duck/Minnesota/Sg-00096/2007
NA(N6)	139~H3N6~MN_2007	CY042065	A/mallard duck/Minnesota/Sg-00098/2007
NA(N6)	14~H6N6~ALB_1982	CY004164	A/blue-winged teal/ALB/266/1982
NA(N6)	140~H4N6~MN_2007	CY042069	A/blue-winged teal/Minnesota/Sg-00102/2007
NA(N6)	141~H3N6~MN_2007	CY042086	A/mallard duck/Minnesota/Sg-00116/2007
NA(N6)	142~H4N6~MN_2007	CY042091	A/mallard duck/Minnesota/Sg-00120/2007
NA(N6)	143~H4N6~TX_2007	CY042097	A/blue-winged teal/Texas/Sg-00124/2007
NA(N6)	144~H3N6~MN_2007	CY042100	A/mallard/Minnesota/Sg-00132/2007
NA(N6)	145~H4N6~MN_2007	CY042101	A/mallard/Minnesota/Sg-00134/2007
NA(N6)	146~H4N6~MN_2007	CY042105	A/blue-winged teal/Minnesota/Sg-00140/2007
NA(N6)	147~H4N6~MN_2007	CY042114	A/blue-winged teal/Minnesota/Sg-00153/2007
NA(N6)	148~H4N6~TX_2007	CY042116	A/blue-winged teal/Texas/Sg-00157/2007
NA(N6)	149~H4N6~TX_2007	CY042117	A/blue-winged teal/Texas/Sg-00158/2007
NA(N6)	15~H6N6~ALB_1982	CY004172	A/mallard/Alberta/289/82
NA(N6)	150~H4N6~LA_2007	CY042128	A/blue-winged teal/Louisiana/Sg-00165/2007
NA(N6)	151~H4N6~MN_2007	CY042138	A/mallard/Minnesota/Sg-00171/2007
NA(N6)	152~H3N6~MN_2007	CY042140	A/mallard/Minnesota/Sg-00174/2007
NA(N6)	153~H4N6~MN_2007	CY042141	A/mallard/Minnesota/Sg-00176/2007
NA(N6)	154~H3N6~MN_2007	CY042157	A/mallard/Minnesota/Sg-00185/2007
NA(N6)	155~H6N6~MN_2007	CY042177	A/Northern pintail/Minnesota/Sg-00196/2007
NA(N6)	156~H4N6~MN_2007	CY042185	A/blue-winged teal/Minnesota/Sg-00201/2007
NA(N6)	157~H4N6~TX_2007	CY042188	A/blue-winged teal/Texas/Sg-00204/2007
NA(N6)	158~H4N6~TX_2007	CY042190	A/blue-winged teal/Texas/Sg-00206/2007
NA(N6)	159~H4N6~TX_2007	CY042191	A/blue-winged teal/Texas/Sg-00207/2007
NA(N6)	16~H3N6~ALB_2003	CY004306	A/pintail/Alberta/166/2003
NA(N6)	160~H4N6~MN_2006	CY042240	A/blue-winged teal/Minnesota/Sg-00229/2006
NA(N6)	161~H4N6~MN_2007	CY042250	A/American wigeon/Minnesota/Sg-00231/2007
NA(N6)	162~H10N6~DE_2008	CY042420	A/ruddy turnstone/Delaware/Sg-00473/2008
NA(N6)	163~H4N6~NJ_2008	CY042422	A/ruddy turnstone/New Jersey/Sg-00475/2008
NA(N6)	164~H4N6~NJ_2008	CY042443	A/ruddy turnstone/New Jersey/Sg-00492/2008
NA(N6)	165~H4N6~NJ_2008	CY042451	A/ruddy turnstone/New Jersey/Sg-00499/2008
NA(N6)	166~H4N6~NJ_2008	CY042455	A/ruddy turnstone/New Jersey/Sg-00502/2008
NA(N6)	167~H4N6~NJ_2008	CY042469	A/ruddy turnstone/New Jersey/Sg-00510/2008
NA(N6)	168~H4N6~NJ_2008	CY042507	A/ruddy turnstone/New Jersey/Sg-00524/2008
NA(N6)	169~H4N6~NJ_2008	CY042523	A/ruddy turnstone/New Jersey/Sg-00531/2008
NA(N6)	17~H4N6~ALB_1976	CY004311	A/mallard duck/ALB/20/1976
NA(N6)	170~H4N6~DE_2008	CY042550	A/ruddy turnstone/Delaware/Sg-00541/2008
NA(N6)	171~H4N6~NJ_2008	CY042558	A/ruddy turnstone/New Jersey/Sg-00545/2008
NA(N6)	172~H4N6~NJ_2008	CY042561	A/ruddy turnstone/New Jersey/Sg-00546/2008
NA(N6)	173~H1N6~DE_2008	CY042572	A/ruddy turnstone/Delaware/Sg-00549/2008
NA(N6)	174~H4N6~NJ_2008	CY042599	A/ruddy turnstone/New Jersey/Sg-00562/2008
NA(N6)	175~H4N6~AK_2007	CY043954	A/northern shoveler/Alaska/7MP1113/2007
NA(N6)	176~H4N6~AK_2007	CY043994	A/northern pintail/Alaska/7MP1393/2007
NA(N6)	177~H4N6~AK_2007	CY044018	A/northern shoveler/Alaska/7MP0954/2007
NA(N6)	178~H4N6~NBR_2006	CY045305	A/American black duck/New Brunswick/19347/2006
NA(N6)	179~H4N6~NBR_2006	CY045313	A/American black duck/New Brunswick/19502/2006
NA(N6)	18~H1N6~ALB_1977	CY004460	A/mallard/Alberta/42/1977
NA(N6)	180~H4N6~CA_2005	CY045337	A/mallard/California/GL30/2005
NA(N6)	181~H4N6~CA_2005	CY045353	A/green-winged teal/California/K218/2005
NA(N6)	182~H4N6~AK_2007	CY045417	A/mallard/Interior Alaska/7MP0372/2007

NA(N6)	183~H4N6~AK_2007	CY045425	A/mallard/Interior Alaska/7MP1050R1/2007
NA(N6)	184~H4N6~AK_2007	CY045457	A/northern shoveler/Interior Alaska/7MP1601/2007
NA(N6)	185~H4N6~ALB_2005	CY047538	A/green-winged teal/Alberta/11383/2005
NA(N6)	186~H4N6~ALB_2005	CY047546	A/redhead/Alberta/11817/2005
NA(N6)	187~H4N6~NSC_2005	CY047570	A/green-winged teal/Nova Scotia/14687/2005
NA(N6)	188~H4N6~QBC_2006	CY047578	A/mallard/Quebec/11102/2006
NA(N6)	189~H4N6~QBC_2006	CY047586	A/mallard/Quebec/11002/2006
NA(N6)	19~H9N6~DE_1988	CY004569	A/ruddy turnstone/DE/773/1988
NA(N6)	190~H4N6~QBC_2006	CY047618	A/mallard/Quebec/11103/2006
NA(N6)	191~H4N6~QBC_2006	CY047626	A/mallard/Quebec/11106/2006
NA(N6)	192~H4N6~QBC_2006	CY047642	A/mallard/Quebec/11182/2006
NA(N6)	193~H4N6~MAN_2007	CY047682	A/American green-winged teal/Manitoba/23884/2007
NA(N6)	194~H3N6~NBR_2007	CY047698	A/American black duck/New Brunswick/25182/2007
NA(N6)	195~H4N6~MN_2007	CY048648	A/blue-winged teal/Minnesota/Sg-00030/2007
NA(N6)	196~H3N6~TX_2007	CY048650	A/blue-winged teal/Texas/Sg-00085/2007
NA(N6)	197~H10N6~MN_2007	CY048667	A/mallard/Minnesota/Sg-00172/2007
NA(N6)	198~H4N6~MN_2008	CY048839	A/northern shoveler/Minnesota/Sg-00647/2008
NA(N6)	199~H4N6~OH_2002	CY053847	A/mallard/Ohio/660/2002
NA(N6)	2~H3N6~ALB_1980	AY207536	A/pintail duck/Alberta/712/80
NA(N6)	20~H3N6~ALB_1977	CY004589	A/blue-winged teal/ALB/286/1977
NA(N6)	200~H3N6~OH_2006	CY053863	A/mallard/Ohio/1506/2006
NA(N6)	201~H4N6~MN_2008	CY054490	A/mallard/Minnesota/Sg-00695/2008
NA(N6)	202~H4N6~MN_2008	CY054492	A/mallard/Minnesota/Sg-00697/2008
NA(N6)	203~H4N6~ND_2008	CY054493	A/blue-winged teal/North Dakota/Sg-00702/2008
NA(N6)	204~H4N6~ND_2008	CY054495	A/blue-winged teal/North Dakota/Sg-00705/2008
NA(N6)	205~H4N6~ND_2008	CY054498	A/blue-winged teal/North Dakota/Sg-00717/2008
NA(N6)	206~H4N6~ND_2008	CY054500	A/blue-winged teal/North Dakota/Sg-00720/2008
NA(N6)	207~H4N6~ND_2008	CY054503	A/blue-winged teal/North Dakota/Sg-00731/2008
NA(N6)	208~H4N6~ND_2008	CY054504	A/blue-winged teal/North Dakota/Sg-00732/2008
NA(N6)	209~H4N6~ND_2008	CY054508	A/blue-winged teal/North Dakota/Sg-00743/2008
NA(N6)	21~H3N6~ALB_1979	CY004615	A/pintail duck/ALB/462/1979
NA(N6)	210~H4N6~ND_2008	CY054512	A/blue-winged teal/North Dakota/Sg-00748/2008
NA(N6)	211~H4N6~ND_2008	CY054513	A/blue-winged teal/North Dakota/Sg-00750/2008
NA(N6)	212~H3N6~MN_2008	CY054514	A/mallard/Minnesota/Sg-00751/2008
NA(N6)	213~H4N6~ND_2008	CY054516	A/blue-winged teal/North Dakota/Sg-00753/2008
NA(N6)	214~H3N6~ND_2008	CY054520	A/blue-winged teal/North Dakota/Sg-00759/2008
NA(N6)	215~H4N6~NJ_2008	CY054521	A/mallard/New Jersey/Sg-00760/2008
NA(N6)	216~H4N6~NJ_2008	CY054523	A/mallard/New Jersey/Sg-00762/2008
NA(N6)	217~H4N6~MN_2008	CY054528	A/mallard/Minnesota/Sg-00769/2008
NA(N6)	218~H4N6~MN_2008	CY054529	A/mallard/Minnesota/Sg-00774/2008
NA(N6)	219~H3N6~MN_2008	CY054532	A/blue-winged teal/Minnesota/Sg-00780/2008
NA(N6)	22~H4N6~ALB_1977	CY004723	A/canvasback duck/ALB/274/1977
NA(N6)	220~H4N6~MN_2008	CY054533	A/mallard/Minnesota/Sg-00793/2008
NA(N6)	221~H4N6~MN_2008	CY054534	A/mallard/Minnesota/Sg-00794/2008
NA(N6)	222~H3N6~MN_2008	CY054535	A/mallard/Minnesota/Sg-00795/2008
NA(N6)	223~H4N6~MN_2008	CY054539	A/blue-winged teal/Minnesota/Sg-00799/2008
NA(N6)	224~H4N6~MN_2008	CY054540	A/blue-winged teal/Minnesota/Sg-00800/2008
NA(N6)	225~H4N6~MN_2008	CY054543	A/mallard/Minnesota/Sg-00807/2008
NA(N6)	226~H4N6~MN_2008	CY054544	A/mallard/Minnesota/Sg-00809/2008
NA(N6)	227~H4N6~SD_2008	CY055111	A/mallard/South Dakota/Sg-00458/2008
NA(N6)	228~H4N6~SD_2008	CY055112	A/mallard/South Dakota/Sg-00459/2008
NA(N6)	229~H4N6~SD_2008	CY055113	A/mallard/South Dakota/Sg-00460/2008
NA(N6)	23~H4N6~ALB_1977	CY004730	A/gadwall duck/ALB/53/1977
NA(N6)	230~H13N6~AK_2009	CY070852	A/glaucous-winged gull/Southcentral Alaska/9JR0691R1/2009
NA(N6)	231~H13N6~AK_2009	CY070860	A/glaucous-winged gull/Southcentral Alaska/9JR0738R1/2009
NA(N6)	232~H13N6~AK_2009	CY070868	A/glaucous-winged gull/Southcentral Alaska/9JR0747R1/2009
NA(N6)	233~H13N6~AK_2009	CY070876	A/glaucous-winged gull/Southcentral Alaska/9JR0769R1/2009
NA(N6)	234~H13N6~AK_2009	CY070884	A/glaucous-winged gull/Southcentral Alaska/9JR0781R1/2009
NA(N6)	235~H4N6~AK_2006	EU557566	A/northern pintail/Alaska/44161-173/2006
NA(N6)	236~H3N6~AK_2006	EU557567	A/northern pintail/Alaska/44187-100/2006
NA(N6)	237~H4N6~AK_2006	EU557568	A/northern pintail/Alaska/44191-123/2006
NA(N6)	238~H3N6~AK_2006	EU557569	A/northern pintail/Alaska/44194-041/2006
NA(N6)	239~H3N6~AK_2006	EU557570	A/northern pintail/Alaska/44194-071/2006
NA(N6)	24~H4N6~ALB_1977	CY004737	A/redhead duck/ALB/74/1977
NA(N6)	240~H4N6~AK_2006	EU557571	A/northern pintail/Alaska/44188-158/2006
NA(N6)	241~H4N6~AK_2006	EU557572	A/northern pintail/Alaska/44188-175/2006
NA(N6)	242~H4N6~AK_2006	EU557573	A/northern pintail/Alaska/44189-069/2006
NA(N6)	243~H4N6~AK_2006	EU557574	A/northern pintail/Alaska/44243-063/2006
NA(N6)	244~H4N6~AK_2006	EU557575	A/northern pintail/Alaska/44203-079/2006
NA(N6)	245~H3N6~MN_1999	EU743538	A/mallard/MN/259/1999
NA(N6)	246~H3N6~MN_2000	EU743546	A/mallard/MN/99/2000
NA(N6)	247~H3N6~MN_2000	EU743554	A/mallard/MN/515/2000
NA(N6)	248~H3N6~MN_1999	EU743562	A/pintail/Minnesota/479/1999
NA(N6)	249~H3N6~NJ_2005	EU871846	A/ruddy turnstone/NJ/335/2005

NA(N6)	25~H4N6~ALB_1977	CY004744	A/blue-winged teal/ALB/243/1977
NA(N6)	250~H3N6~NJ_2005	EU871854	A/ruddy turnstone/NJ/357/2005
NA(N6)	251~H3N6~NJ_2005	EU871862	A/sanderling/NJ/1042/2005
NA(N6)	252~H4N6~MN_1999	EU871878	A/mallard/Minnesota/160/1999
NA(N6)	253~H4N6~MN_1999	EU871886	A/mallard/Minnesota/269/1999
NA(N6)	254~H4N6~MN_1999	FJ517306	A/mallard/Minnesota/192/1999
NA(N6)	255~H4N6~MN_2000	FJ517310	A/mallard/Minnesota/348/2000
NA(N6)	256~H3N6~CA_2006	FJ520138	A/northern pintail/California/44242-711/2006
NA(N6)	257~H3N6~CA_2006	FJ520150	A/northern pintail/California/44242-836/2006
NA(N6)	258~H4N6~CA_2006	FJ520151	A/northern pintail/California/44249-38/2006
NA(N6)	259~H4N6~CA_2007	FJ520163	A/northern pintail/California/44355-442/2007
NA(N6)	26~H4N6~ALB_1977	CY004764	A/pintail duck/ALB/159/1977
NA(N6)	260~H11N6~NJ_2005	GU051091	A/ruddy turnstone/New Jersey/1058/2005
NA(N6)	261~H3N6~NJ_2005	GU051154	A/ruddy turnstone/New Jersey/327/2005
NA(N6)	262~H3N6~NJ_2005	GU051165	A/ruddy turnstone/New Jersey/950/2005
NA(N6)	263~H3N6~NJ_2005	GU051172	A/ruddy turnstone/New Jersey/975/2005
NA(N6)	264~H4N6~MN_1999	GU051229	A/mallard/Minnesota/145/1999
NA(N6)	265~H4N6~MN_1999	GU051233	A/mallard/Minnesota/193/1999
NA(N6)	266~H4N6~MN_2000	GU051241	A/mallard/Minnesota/313/2000
NA(N6)	267~H4N6~MN_2000	GU051247	A/mallard/Minnesota/524/2000
NA(N6)	268~H13N6~MA_1980	GU051930	A/gull/Massachusetts/26/1980
NA(N6)	269~H2N6~OH_2002	GU053446	A/mallard/Ohio/669/2002
NA(N6)	27~H4N6~ALB_1977	CY004771	A/mallard duck/ALB/161/1977
NA(N6)	270~H4N6~OH_2002	GU053454	A/mallard/Ohio/684/2002
NA(N6)	271~H4N6~AK_2007	GU168383	A/northern pintail/Alaska/44344-191/2007
NA(N6)	272~H4N6~AK_2008	GU168384	A/northern pintail/Alaska/44430-290/2008
NA(N6)	273~H3N6~AK_2008	GU168385	A/northern pintail/Alaska/44421-431/2008
NA(N6)	274~H4N6~AK_2008	GU168386	A/northern pintail/Alaska/44421-444/2008
NA(N6)	275~H3N6~AK_2007	GU168387	A/northern pintail/Alaska/44344-730/2007
NA(N6)	276~H3N6~AK_2007	GU168388	A/northern pintail/Alaska/44344-881/2007
NA(N6)	277~H3N6~AK_2008	GU168389	A/northern pintail/Alaska/44419-104/2008
NA(N6)	278~H4N6~AK_2008	GU168390	A/northern pintail/Alaska/44419-175/2008
NA(N6)	279~H3N6~AK_2008	GU168392	A/northern pintail/Alaska/44419-062/2008
NA(N6)	28~H4N6~ALB_1985	CY004839	A/mallard duck/ALB/25/1985
NA(N6)	280~H3N6~AK_2007	GU168393	A/northern pintail/Alaska/44344-503/2007
NA(N6)	281~H3N6~AK_2008	GU168394	A/northern pintail/Alaska/44421-446/2008
NA(N6)	282~H4N6~AK_2007	GU168395	A/northern pintail/Alaska/44344-821/2007
NA(N6)	283~H3N6~AK_2007	GU168397	A/northern pintail/Alaska/44344-798/2007
NA(N6)	284~H3N6~AK_2007	GU168398	A/northern pintail/Alaska/44340-648/2007
NA(N6)	285~H4N6~AK_2007	GU168399	A/northern pintail/Alaska/44344-880/2007
NA(N6)	286~H7N6~NC_2005	GU186476	A/Northern shoveler/NC/6412-052/2005
NA(N6)	287~H2N6~AK_2006	HM060031	A/thick-billed murre/Alaska/44145-199/2006
NA(N6)	29~H4N6~ALB_1985	CY004844	A/redhead duck/ALB/116/1985
NA(N6)	3~H4N6~ALB_1998	AY207554	A/mallard/Alberta/213/98
NA(N6)	30~H4N6~ALB_1985	CY004855	A/pintail duck/ALB/623/1985
NA(N6)	31~H4N6~DE_1988	CY004862	A/ruddy turnstone/DE/512/1988
NA(N6)	32~H4N6~DE_1988	CY004869	A/knot/DE/541/1988
NA(N6)	33~H4N6~DE_1988	CY004876	A/herring gull/DE/665/1988
NA(N6)	34~H4N6~ALB_1994	CY004905	A/blue-winged teal/ALB/293/1994
NA(N6)	35~H4N6~ALB_1995	CY004913	A/mallard/ALB/49/1995
NA(N6)	36~H4N6~ALB_1996	CY004920	A/pintail/ALB/275/1996
NA(N6)	37~H4N6~ALB_2001	CY004948	A/pintail/Alberta/269/2001
NA(N6)	38~H4N6~ALB_2003	CY004955	A/blue-winged teal/Alberta/293/2003
NA(N6)	39~H9N6~DE_1988	CY005135	A/ruddy turnstone/DE/510/1988
NA(N6)	4~H4N6~ALB_1999	AY633126	A/mallard/Alberta/111/99
NA(N6)	40~H10N6~ALB_1984	CY005201	A/mallard duck/ALB/40/1984
NA(N6)	41~H10N6~ALB_1984	CY005208	A/pintail duck/ALB/584/1984
NA(N6)	42~H11N6~ALB_1999	CY005319	A/mallard/Alberta/125/1999
NA(N6)	43~H13N6~DE_1988	CY005387	A/herring gull/Delaware/660/1988
NA(N6)	44~H6N6~DE_1986	CY005422	A/sanderling/Delaware/1258/1986
NA(N6)	45~H13N6~MN_1980	CY005860	A/gull/Minnesota/945/1980
NA(N6)	46~H6N6~MN_1980	CY005883	A/blue-winged teal/MN/993/1980
NA(N6)	47~H4N6~OH_2002	CY011038	A/mallard/Ohio/657/2002
NA(N6)	48~H4N6~OH_1987	CY012810	A/mallard/Ohio/298/1987
NA(N6)	49~H3N6~AK_2005	CY013265	A/pintail/Alaska/53/2005
NA(N6)	5~H4N6~ALB_2000	AY633140	A/mallard/Alberta/119/00
NA(N6)	50~H3N6~ALB_1985	CY014550	A/mallard duck/Alberta/331/1985
NA(N6)	51~H13N6~MD_1977	CY014696	A/gull/Maryland/704/1977
NA(N6)	52~H3N6~NY_1986	CY014867	A/mallard duck/New York/157/1986
NA(N6)	53~H13N6~DE_1997	CY015148	A/shorebird/Delaware/224/1997
NA(N6)	54~H4N6~OH_1986	CY015469	A/mallard/Ohio/83/1986
NA(N6)	55~H4N6~OH_1988	CY017703	A/mallard/Ohio/324/1988
NA(N6)	56~H3N6~OH_1990	CY017711	A/mallard/Ohio/156/1990
NA(N6)	57~H4N6~AK_2005	CY017743	A/pintail/Alaska/310/2005

NA(N6)	58~H4N6~OH_2002	CY020727	A/mallard/Ohio/667/2002
NA(N6)	59~H4N6~MD_2002	CY020735	A/mallard/Maryland/750/2002
NA(N6)	6~H4N6~ALB_2000	AY633158	A/mallard/Alberta/136/00
NA(N6)	60~H4N6~OH_2002	CY020751	A/blue-winged teal/Ohio/989/2002
NA(N6)	61~H4N6~OH_2002	CY020775	A/mallard/Ohio/655/2002
NA(N6)	62~H4N6~OH_2002	CY020791	A/mallard/Ohio/668/2002
NA(N6)	63~H4N6~OH_2002	CY020799	A/mallard/Ohio/671/2002
NA(N6)	64~H6N6~OH_2002	CY020855	A/mallard/Ohio/664/2002
NA(N6)	65~H1N6~OH_2002	CY020863	A/blue-winged teal/Ohio/907/2002
NA(N6)	66~H4N6~OH_1989	CY020983	A/mallard/Ohio/129/1989
NA(N6)	67~H4N6~OH_1990	CY021215	A/mallard/Ohio/178/1990
NA(N6)	68~H4N6~OH_2005	CY021223	A/blue-winged teal/Ohio/1339/2005
NA(N6)	69~H4N6~OH_2005	CY021327	A/green-winged teal/Ohio/1324/2005
NA(N6)	7~H3N6~ALB_1999	AY633174	A/mallard/Alberta/199/1999
NA(N6)	70~H4N6~MD_2005	CY021335	A/mallard/Maryland/1241/2005
NA(N6)	71~H4N6~MD_2005	CY021895	A/white-winged scoter/Maryland/301/2005
NA(N6)	72~H4N6~OH_2005	CY022607	A/green-winged teal/Ohio/1292/2005
NA(N6)	73~H3N6~OH_2005	CY022719	A/mallard/Ohio/1717/2005
NA(N6)	74~H3N6~MD_2006	CY028694	A/mallard/Maryland/1235/2006
NA(N6)	75~H4N6~AK_2007	CY036653	A/northern pintail/Interior Alaska/1/2007
NA(N6)	76~H4N6~DE_2008	CY038037	A/ruddy turnstone/Delaware/Sg-00470/2008
NA(N6)	77~H4N6~NJ_2008	CY038052	A/ruddy turnstone/New Jersey/Sg-00477/2008
NA(N6)	78~H4N6~NJ_2008	CY038062	A/red knot/New Jersey/Sg-00479/2008
NA(N6)	79~H4N6~NJ_2008	CY038067	A/ruddy turnstone/New Jersey/Sg-00480/2008
NA(N6)	8~H4N6~ALB_1998	AY633262	A/mallard/Alberta/295/98
NA(N6)	80~H4N6~NJ_2008	CY038092	A/ruddy turnstone/New Jersey/Sg-00489/2008
NA(N6)	81~H4N6~NJ_2008	CY038107	A/ruddy turnstone/New Jersey/Sg-00494/2008
NA(N6)	82~H4N6~NJ_2008	CY038117	A/ruddy turnstone/New Jersey/Sg-00496/2008
NA(N6)	83~H4N6~NJ_2008	CY038142	A/ruddy turnstone/New Jersey/Sg-00506/2008
NA(N6)	84~H4N6~NJ_2008	CY038147	A/ruddy turnstone/New Jersey/Sg-00507/2008
NA(N6)	85~H4N6~NJ_2008	CY038182	A/ruddy turnstone/New Jersey/Sg-00527/2008
NA(N6)	86~H4N6~NJ_2008	CY038187	A/ruddy turnstone/New Jersey/Sg-00533/2008
NA(N6)	87~H4N6~NJ_2008	CY038192	A/ruddy turnstone/New Jersey/Sg-00534/2008
NA(N6)	88~H4N6~DE_2008	CY038197	A/ruddy turnstone/Delaware/Sg-00539/2008
NA(N6)	89~H4N6~NJ_2008	CY038202	A/ruddy turnstone/New Jersey/Sg-00542/2008
NA(N6)	9~H4N6~ALB_1998	AY633270	A/mallard/Alberta/30/98
NA(N6)	90~H4N6~NJ_2008	CY038207	A/ruddy turnstone/New Jersey/Sg-00550/2008
NA(N6)	91~H6N6~NJ_2008	CY038212	A/ruddy turnstone/New Jersey/Sg-00551/2008
NA(N6)	92~H4N6~NJ_2008	CY038217	A/ruddy turnstone/New Jersey/Sg-00552/2008
NA(N6)	93~H4N6~NJ_2008	CY038222	A/ruddy turnstone/New Jersey/Sg-00556/2008
NA(N6)	94~H4N6~NJ_2008	CY038227	A/ruddy turnstone/New Jersey/Sg-00558/2008
NA(N6)	95~H4N6~NJ_2008	CY038252	A/ruddy turnstone/New Jersey/Sg-00565/2008
NA(N6)	96~H4N6~MN_2008	CY038257	A/mallard/Minnesota/Sg-00569/2008
NA(N6)	97~H4N6~CA_2008	CY039741	A/mallard/California/7766/2008
NA(N6)	98~H4N6~AK_2007	CY039772	A/mallard/Interior Alaska/6/2007
NA(N6)	99~H4N6~AK_2007	CY039788	A/mallard/Interior Alaska/4/2007
NA(N8)	1~H3N8~ALB_1997	AY633134	A/mallard/Alberta/117/97
NA(N8)	10~H3N8~ALB_1999	AY633374	A/pintail/Alberta/37/99
NA(N8)	100~H3N8~MN_2007	CY042008	A/mallard duck/Minnesota/Sg-00048/2007
NA(N8)	101~H3N8~MN_2007	CY042011	A/mallard duck/Minnesota/Sg-00051/2007
NA(N8)	102~H3N8~MN_2007	CY042022	A/mallard duck/Minnesota/Sg-00059/2007
NA(N8)	103~H3N8~MN_2007	CY042024	A/mallard duck/Minnesota/Sg-00061/2007
NA(N8)	104~H3N8~MN_2007	CY042028	A/mallard duck/Minnesota/Sg-00064/2007
NA(N8)	105~H3N8~MN_2007	CY042030	A/ring-necked duck/Minnesota/Sg-00066/2007
NA(N8)	106~H3N8~MN_2007	CY042033	A/ring-necked duck/Minnesota/Sg-00069/2007
NA(N8)	107~H4N8~MN_2007	CY042035	A/mallard duck/Minnesota/Sg-00070/2007
NA(N8)	108~H4N8~MN_2007	CY042037	A/mallard duck/Minnesota/Sg-00071/2007
NA(N8)	109~H3N8~MN_2007	CY042038	A/mallard duck/Minnesota/Sg-00072/2007
NA(N8)	11~H6N8~ALB_1994	AY633382	A/Redhead/Alberta/291/94
NA(N8)	110~H3N8~TX_2007	CY042043	A/blue-winged teal/Texas/Sg-00078/2007
NA(N8)	111~H3N8~TX_2007	CY042045	A/blue-winged teal/Texas/Sg-00079/2007
NA(N8)	112~H3N8~TX_2007	CY042047	A/blue-winged teal/Texas/Sg-00082/2007
NA(N8)	113~H3N8~TX_2007	CY042049	A/blue-winged teal/Texas/Sg-00084/2007
NA(N8)	114~H3N8~LA_2007	CY042058	A/green-winged teal/Louisiana/Sg-00092/2007
NA(N8)	115~H3N8~MN_2007	CY042064	A/mallard duck/Minnesota/Sg-00097/2007
NA(N8)	116~H3N8~MN_2007	CY042070	A/mallard duck/Minnesota/Sg-00103/2007
NA(N8)	117~H3N8~MN_2007	CY042088	A/mallard duck/Minnesota/Sg-00117/2007
NA(N8)	118~H3N8~MN_2007	CY042090	A/mallard duck/Minnesota/Sg-00119/2007
NA(N8)	119~H4N8~TX_2007	CY042115	A/blue-winged teal/Texas/Sg-00155/2007
NA(N8)	12~H2N8~DE_1988	CY003916	A/herring gull/DE/703/1988
NA(N8)	120~H4N8~TX_2007	CY042118	A/blue-winged teal/Texas/Sg-00159/2007
NA(N8)	121~H3N8~MN_2007	CY042120	A/mallard/Minnesota/Sg-00160/2007
NA(N8)	122~H3N8~MN_2007	CY042122	A/mallard/Minnesota/Sg-00161/2007
NA(N8)	123~H4N8~LA_2007	CY042126	A/blue-winged teal/Louisiana/Sg-00163/2007

NA(N8)	124~H3N8~MN_2007	CY042127	A/mallard/Minnesota/Sg-00164/2007
NA(N8)	125~H4N8~TX_2007	CY042139	A/blue-winged teal/Texas/Sg-00173/2007
NA(N8)	126~H3N8~MN_2007	CY042152	A/mallard/Minnesota/Sg-00184/2007
NA(N8)	127~H4N8~TX_2007	CY042164	A/blue-winged teal/Texas/Sg-00188/2007
NA(N8)	128~H3N8~MN_2007	CY042169	A/mallard/Minnesota/Sg-00190/2007
NA(N8)	129~H3N8~MN_2007	CY042171	A/mallard/Minnesota/Sg-00191/2007
NA(N8)	13~H6N8~ALB_1978	CY004056	A/mallard duck/ALB/761/1978
NA(N8)	130~H3N8~MN_2007	CY042172	A/mallard/Minnesota/Sg-00192/2007
NA(N8)	131~H3N8~MN_2007	CY042184	A/mallard/Minnesota/Sg-00200/2007
NA(N8)	132~H3N8~MN_2007	CY042186	A/mallard/Minnesota/Sg-00202/2007
NA(N8)	133~H3N8~MN_2007	CY042187	A/mallard/Minnesota/Sg-00203/2007
NA(N8)	134~H3N8~TX_2007	CY042189	A/blue-winged teal/Texas/Sg-00205/2007
NA(N8)	135~H3N8~MN_2007	CY042196	A/mallard/Minnesota/Sg-00209/2007
NA(N8)	136~H3N8~MN_2007	CY042198	A/mallard/Minnesota/Sg-00211/2007
NA(N8)	137~H3N8~MN_2007	CY042201	A/mallard/Minnesota/Sg-00215/2007
NA(N8)	138~H3N8~LA_2007	CY042210	A/blue-winged teal/Louisiana/Sg-00218/2007
NA(N8)	139~H3N8~LA_2007	CY042215	A/blue-winged teal/Louisiana/Sg-00224/2007
NA(N8)	14~H6N8~DE_1988	CY004090	A/knot/DE/526/1988
NA(N8)	140~H3N8~MN_2006	CY042225	A/blue-winged teal/Minnesota/Sg-00226/2006
NA(N8)	141~H6N8~MN_2006	CY042235	A/green-winged teal/Minnesota/Sg-00228/2006
NA(N8)	142~H6N8~NJ_2008	CY042411	A/ruddy turnstone/New Jersey/Sg-00471/2008
NA(N8)	143~H6N8~NJ_2008	CY042426	A/ruddy turnstone/New Jersey/Sg-00482/2008
NA(N8)	144~H3N8~MN_2008	CY042619	A/mallard/Minnesota/Sg-00573/2008
NA(N8)	145~H3N8~MN_2008	CY042623	A/mallard/Minnesota/Sg-00574/2008
NA(N8)	146~H3N8~MN_2008	CY042628	A/gadwall duck/Minnesota/Sg-00575/2008
NA(N8)	147~H4N8~MN_2008	CY042644	A/mallard/Minnesota/Sg-00624/2008
NA(N8)	148~H4N8~MN_2008	CY042649	A/mallard/Minnesota/Sg-00625/2008
NA(N8)	149~H4N8~MN_2008	CY042654	A/mallard/Minnesota/Sg-00626/2008
NA(N8)	15~H6N8~NJ_1989	CY004096	A/laughing gull/NJ/276/1989
NA(N8)	150~H3N8~MN_2008	CY042667	A/mallard/Minnesota/Sg-00629/2008
NA(N8)	151~H4N8~MN_2008	CY042676	A/mallard/Minnesota/Sg-00631/2008
NA(N8)	152~H3N8~MN_2008	CY042684	A/mallard/Minnesota/Sg-00633/2008
NA(N8)	153~H3N8~MN_2008	CY042689	A/mallard/Minnesota/Sg-00634/2008
NA(N8)	154~H4N8~MN_2008	CY042694	A/mallard/Minnesota/Sg-00635/2008
NA(N8)	155~H3N8~MN_2008	CY042699	A/mallard/Minnesota/Sg-00636/2008
NA(N8)	156~H3N8~MN_2008	CY042704	A/mallard/Minnesota/Sg-00637/2008
NA(N8)	157~H3N8~MN_2008	CY042709	A/mallard/Minnesota/Sg-00638/2008
NA(N8)	158~H3N8~MN_2008	CY042714	A/mallard/Minnesota/Sg-00639/2008
NA(N8)	159~H6N8~CA_2005	CY043810	A/ring-necked duck/California/K90/2005
NA(N8)	16~H3N8~ALB_1979	CY004108	A/mallard duck/ALB/564/1979
NA(N8)	160~H3N8~AK_2007	CY043938	A/northern pintail/Alaska/7MP0344/2007
NA(N8)	161~H3N8~AK_2007	CY043946	A/northern pintail/Alaska/7MP0608/2007
NA(N8)	162~H3N8~AK_2007	CY043962	A/northern shoveler/Alaska/7MP1026/2007
NA(N8)	163~H4N8~AK_2007	CY043970	A/mallard/Alaska/7MP1028/2007
NA(N8)	164~H3N8~AK_2007	CY043978	A/American widgeon/Alaska/7MP1061/2007
NA(N8)	165~H3N8~AK_2007	CY043986	A/northern pintail/Alaska/7MP1092/2007
NA(N8)	166~H4N8~AK_2007	CY044002	A/least sandpiper/Alaska/7KW0411/2007
NA(N8)	167~H3N8~AK_2007	CY044010	A/northern shoveler/Alaska/7MP1606/2007
NA(N8)	168~H3N8~AK_2007	CY044026	A/northern shoveler/Alaska/7MP1668/2007
NA(N8)	169~H3N8~WA_2006	CY045369	A/mallard/Washington/20010-002/2006
NA(N8)	17~H6N8~ALB_1979	CY004116	A/pintail duck/ALB/628/1979
NA(N8)	170~H3N8~AK_2007	CY045409	A/northern pintail/Interior Alaska/7MP0343/2007
NA(N8)	171~H3N8~AK_2007	CY045433	A/mallard/Interior Alaska/7MP0709/2007
NA(N8)	172~H3N8~AK_2007	CY045441	A/northern shoveler/Alaska/7MP1708/2007
NA(N8)	173~H3N8~AK_2007	CY045449	A/northern pintail/Alaska/7MP0508/2007
NA(N8)	174~H3N8~AK_2007	CY047029	A/American green-winged teal/Interior Alaska/7MP2225/2007
NA(N8)	175~H3N8~AK_2007	CY047037	A/mallard/Interior Alaska/7MP1757/2007
NA(N8)	176~H3N8~BCO_2005	CY047490	A/mallard/British Columbia/07706/2005
NA(N8)	177~H3N8~BCO_2005	CY047506	A/mallard/British Columbia/07569/2005
NA(N8)	178~H3N8~ALB_2005	CY047514	A/mallard/Alberta/11527/2005
NA(N8)	179~H3N8~ALB_2005	CY047522	A/blue-winged teal/Alberta/11646/2005
NA(N8)	18~H6N8~ALB_1996	CY004268	A/Mallard/Alberta/206/1996
NA(N8)	180~H3N8~ALB_2005	CY047530	A/northern pintail/Alberta/11701/2005
NA(N8)	181~H3N8~QBC_2006	CY047610	A/mallard/Quebec/11082/2006
NA(N8)	182~H4N8~TX_2007	CY048649	A/blue-winged teal/Texas/Sg-00074/2007
NA(N8)	183~H3N8~MN_2007	CY048662	A/mallard/Minnesota/Sg-00168/2007
NA(N8)	184~H4N8~MN_2008	CY048775	A/ring-necked duck/Minnesota/Sg-00449/2008
NA(N8)	185~H4N8~MN_2008	CY048776	A/blue-winged teal/Minnesota/Sg-00450/2008
NA(N8)	186~H4N8~MN_2008	CY048777	A/blue-winged teal/Minnesota/Sg-00451/2008
NA(N8)	187~H3N8~SD_2008	CY048781	A/northern pintail/South Dakota/Sg-00461/2008
NA(N8)	188~H3N8~MN_2008	CY048782	A/mallard/Minnesota/Sg-00463/2008
NA(N8)	189~H4N8~MN_2008	CY048784	A/mallard/Minnesota/Sg-00465/2008
NA(N8)	19~H6N8~DE_2004	CY004284	A/shorebird/DE/12/2004
NA(N8)	190~H3N8~MN_2008	CY048826	A/mallard/Minnesota/Sg-00640/2008

NA(N8)	191~H3N8~MN_2008	CY048828	A/mallard/Minnesota/Sg-00641/2008
NA(N8)	192~H3N8~MN_2008	CY048830	A/mallard/Minnesota/Sg-00642/2008
NA(N8)	193~H3N8~MN_2008	CY048831	A/mallard/Minnesota/Sg-00643/2008
NA(N8)	194~H3N8~MN_2008	CY048833	A/mallard/Minnesota/Sg-00644/2008
NA(N8)	195~H3N8~MN_2008	CY048835	A/northern shoveler/Minnesota/Sg-00645/2008
NA(N8)	196~H3N8~MN_2008	CY048843	A/blue-winged teal/Minnesota/Sg-00649/2008
NA(N8)	197~H3N8~MN_2008	CY048845	A/blue-winged teal/Minnesota/Sg-00650/2008
NA(N8)	198~H3N8~MN_2008	CY048849	A/northern shoveler/Minnesota/Sg-00652/2008
NA(N8)	199~H3N8~MN_2008	CY048852	A/northern shoveler/Minnesota/Sg-00654/2008
NA(N8)	2~H3N8~ALB_2000	AY633150	A/mallard/Alberta/127/00
NA(N8)	20~H3N8~ALB_1976	CY004301	A/mallard duck/ALB/31/1976
NA(N8)	200~H3N8~MN_2008	CY048856	A/northern shoveler/Minnesota/Sg-00656/2008
NA(N8)	201~H3N8~MN_2008	CY048858	A/northern shoveler/Minnesota/Sg-00657/2008
NA(N8)	202~H3N8~MN_2008	CY048859	A/northern shoveler/Minnesota/Sg-00658/2008
NA(N8)	203~H3N8~MN_2008	CY048861	A/northern shoveler/Minnesota/Sg-00659/2008
NA(N8)	204~H3N8~MN_2008	CY048863	A/northern shoveler/Minnesota/Sg-00660/2008
NA(N8)	205~H3N8~MN_2008	CY048866	A/northern shoveler/Minnesota/Sg-00662/2008
NA(N8)	206~H3N8~MN_2008	CY048868	A/northern shoveler/Minnesota/Sg-00663/2008
NA(N8)	207~H3N8~MN_2008	CY048870	A/northern shoveler/Minnesota/Sg-00664/2008
NA(N8)	208~H3N8~MN_2008	CY048873	A/northern shoveler/Minnesota/Sg-00666/2008
NA(N8)	209~H3N8~MN_2008	CY048874	A/northern shoveler/Minnesota/Sg-00667/2008
NA(N8)	21~H2N8~DE_1988	CY004556	A/herring gull/DE/692/1988
NA(N8)	210~H3N8~MN_2008	CY048876	A/northern shoveler/Minnesota/Sg-00668/2008
NA(N8)	211~H3N8~MN_2008	CY048878	A/northern shoveler/Minnesota/Sg-00669/2008
NA(N8)	212~H3N8~MN_2008	CY048881	A/northern shoveler/Minnesota/Sg-00671/2008
NA(N8)	213~H3N8~MN_2008	CY048883	A/mallard/Minnesota/Sg-00672/2008
NA(N8)	214~H3N8~MN_2008	CY048887	A/mallard/Minnesota/Sg-00674/2008
NA(N8)	215~H3N8~MN_2008	CY048891	A/mallard/Minnesota/Sg-00676/2008
NA(N8)	216~H3N8~MN_2008	CY048893	A/mallard/Minnesota/Sg-00677/2008
NA(N8)	217~H3N8~MN_2008	CY048897	A/mallard/Minnesota/Sg-00679/2008
NA(N8)	218~H3N8~MN_2008	CY048905	A/mallard/Minnesota/Sg-00683/2008
NA(N8)	219~H3N8~MN_2008	CY048911	A/mallard/Minnesota/Sg-00687/2008
NA(N8)	22~H3N8~ALB_1979	CY004608	A/green-winged teal/ALB/438/1979
NA(N8)	220~H3N8~OH_2002	CY053831	A/mallard/Ohio/651/2002
NA(N8)	221~H3N8~OH_2002	CY053839	A/green-winged teal/Ohio/960/2002
NA(N8)	222~H4N8~MN_2008	CY054488	A/blue-winged teal/Minnesota/Sg-00691/2008
NA(N8)	223~H3N8~MN_2008	CY054491	A/mallard/Minnesota/Sg-00696/2008
NA(N8)	224~H3N8~ND_2008	CY054494	A/blue-winged teal/North Dakota/Sg-00704/2008
NA(N8)	225~H4N8~ND_2008	CY054496	A/blue-winged teal/North Dakota/Sg-00707/2008
NA(N8)	226~H4N8~ND_2008	CY054497	A/blue-winged teal/North Dakota/Sg-00711/2008
NA(N8)	227~H3N8~ND_2008	CY054499	A/blue-winged teal/North Dakota/Sg-00719/2008
NA(N8)	228~H3N8~ND_2008	CY054502	A/blue-winged teal/North Dakota/Sg-00723/2008
NA(N8)	229~H6N8~ND_2008	CY054505	A/mallard/North Dakota/Sg-00737/2008
NA(N8)	23~H3N8~ALB_1985	CY004664	A/mallard duck/ALB/525/1985
NA(N8)	230~H3N8~ND_2008	CY054506	A/blue-winged teal/North Dakota/Sg-00739/2008
NA(N8)	231~H4N8~ND_2008	CY054507	A/blue-winged teal/North Dakota/Sg-00741/2008
NA(N8)	232~H3N8~ND_2008	CY054511	A/blue-winged teal/North Dakota/Sg-00747/2008
NA(N8)	233~H4N8~ND_2008	CY054518	A/blue-winged teal/North Dakota/Sg-00757/2008
NA(N8)	234~H3N8~ND_2008	CY054519	A/blue-winged teal/North Dakota/Sg-00758/2008
NA(N8)	235~H4N8~NJ_2008	CY054522	A/mallard/New Jersey/Sg-00761/2008
NA(N8)	236~H3N8~MN_2008	CY054524	A/mallard/Minnesota/Sg-00764/2008
NA(N8)	237~H3N8~MN_2008	CY054525	A/mallard/Minnesota/Sg-00765/2008
NA(N8)	238~H4N8~MN_2008	CY054526	A/mallard/Minnesota/Sg-00767/2008
NA(N8)	239~H3N8~MN_2008	CY054531	A/green-winged teal/Minnesota/Sg-00779/2008
NA(N8)	24~H3N8~ALB_1985	CY004672	A/blue-winged teal/ALB/569/1985
NA(N8)	240~H3N8~MN_2008	CY054542	A/mallard/Minnesota/Sg-00804/2008
NA(N8)	241~H3N8~MN_2008	CY055109	A/blue-winged teal/Minnesota/Sg-00453/2008
NA(N8)	242~H3N8~MN_2008	CY055120	A/mallard/Minnesota/Sg-00580/2008
NA(N8)	243~H3N8~MN_2008	CY055121	A/northern shoveler/Minnesota/Sg-00653/2008
NA(N8)	244~H3N8~MN_2008	CY055123	A/northern shoveler/Minnesota/Sg-00670/2008
NA(N8)	245~H3N8~AK_2006	EU557579	A/northern pintail/Alaska/44155-158/2006
NA(N8)	246~H3N8~AK_2006	EU557580	A/northern pintail/Alaska/44160-044/2006
NA(N8)	247~H3N8~AK_2006	EU557581	A/northern pintail/Alaska/44183-067/2006
NA(N8)	248~H3N8~AK_2006	EU557582	A/northern pintail/Alaska/44183-072/2006
NA(N8)	249~H3N8~AK_2006	EU557583	A/northern pintail/Alaska/44182-129/2006
NA(N8)	25~H3N8~ALB_2001	CY004704	A/mallard/Alberta/156/2001
NA(N8)	250~H3N8~AK_2006	EU557584	A/northern pintail/Alaska/44184-003/2006
NA(N8)	251~H3N8~AK_2006	EU557585	A/northern pintail/Alaska/44244-108/2006
NA(N8)	252~H3N8~AK_2006	EU557586	A/northern pintail/Alaska/44228-129/2006
NA(N8)	253~H3N8~AK_2006	EU557587	A/northern pintail/Alaska/44228-173/2006
NA(N8)	254~H3N8~AK_2006	EU557588	A/northern pintail/Alaska/44228-175/2006
NA(N8)	255~H6N8~AK_2006	EU557589	A/northern pintail/Alaska/44203-078/2006
NA(N8)	256~H3N8~AK_2006	EU557590	A/northern pintail/Alaska/44202-126/2006
NA(N8)	257~H3N8~AK_2006	EU557591	A/northern pintail/Alaska/44204-075/2006

NA(N8)	258~H3N8~AK_2006	EU557592	A/northern pintail/Alaska/44202-103/2006
NA(N8)	259~H4N8~MN_1999	EU871894	A/mallard/MN/323/1999
NA(N8)	26~H4N8~ALB_1977	CY004757	A/mallard duck/ALB/210/1977
NA(N8)	260~H3N8~MN_1999	FJ517284	A/mallard/Minnesota/190/1999
NA(N8)	261~H3N8~MN_1999	FJ517290	A/mallard/Minnesota/283/1999
NA(N8)	262~H3N8~MN_1999	FJ517297	A/mallard/Minnesota/354/1999
NA(N8)	263~H4N8~MN_1999	FJ517316	A/mallard/MN/327/1999
NA(N8)	264~H4N8~OH_1986	FJ517338	A/mallard/Ohio/338/1986
NA(N8)	265~H10N8~CA_2006	FJ520145	A/northern pintail/California/44221-656/2006
NA(N8)	266~H3N8~CA_2006	FJ520155	A/northern pintail/California/44221-794/2006
NA(N8)	267~H3N8~CA_2006	FJ520157	A/northern pintail/California/44241-743/2006
NA(N8)	268~H3N8~AK_2006	GQ168612	A/Pacific golden plover/Alaska/44201-109/2006
NA(N8)	269~H11N8~NJ_2005	GU051103	A/ruddy turnstone/New Jersey/680/2005
NA(N8)	27~H4N8~ALB_1977	CY004778	A/pintail duck/ALB/220/1977
NA(N8)	270~H3N8~MN_2000	GU051149	A/mallard/Minnesota/448/2000
NA(N8)	271~H3N8~MN_1999	GU051182	A/mallard/Minnesota/231/1999
NA(N8)	272~H3N8~NJ_2005	GU051192	A/ruddy turnstone/New Jersey/918/2005
NA(N8)	273~H3N8~WA_1978	GU052301	A/American black duck/Washington/699/1978
NA(N8)	274~H3N8~AK_2007	GU168403	A/northern pintail/Alaska/44340-347/2007
NA(N8)	275~H3N8~AK_2007	GU168404	A/northern pintail/Alaska/44340-093/2007
NA(N8)	276~H3N8~AK_2008	GU168405	A/northern pintail/Alaska/44430-114/2008
NA(N8)	277~H3N8~AK_2007	GU168406	A/northern pintail/Alaska/44340-071/2007
NA(N8)	278~H3N8~AK_2008	GU168407	A/northern pintail/Alaska/44421-445/2008
NA(N8)	279~H1N8~AK_2008	GU168408	A/northern pintail/Alaska/44415-640/2008
NA(N8)	28~H4N8~ALB_1999	CY004935	A/pintail/Alberta/207/1999
NA(N8)	280~H3N8~AK_2007	GU168409	A/northern pintail/Alaska/44344-581/2007
NA(N8)	281~H3N8~AK_2007	GU168410	A/northern pintail/Alaska/44340-268/2007
NA(N8)	282~H3N8~AK_2008	GU168412	A/northern pintail/Alaska/44430-266/2008
NA(N8)	283~H3N8~AK_2007	GU168413	A/northern pintail/Alaska/44344-490/2007
NA(N8)	284~H3N8~AK_2007	GU168414	A/northern pintail/Alaska/44340-297/2007
NA(N8)	285~H3N8~AK_2008	GU168415	A/northern pintail/Alaska/44421-385/2008
NA(N8)	286~H3N8~AK_2007	GU168416	A/northern pintail/Alaska/44344-506/2007
NA(N8)	287~H3N8~AK_2007	GU168417	A/northern pintail/Alaska/44340-184/2007
NA(N8)	288~H3N8~NJ_2005	GU186468	A/laughing gull/NJ/768/2005
NA(N8)	289~H3N8~AK_2006	HM060032	A/glaucous gull/Alaska/44201-161/2006
NA(N8)	29~H4N8~ALB_2001	CY004941	A/mallard/Alberta/30/2001
NA(N8)	290~H2N8~DE_1988	L06585	A/herring gull/DE/677/1988
NA(N8)	291~H3N8~ALB_1990	L06586	A/mallard/Edmonton/220/1990
NA(N8)	3~H6N8~ALB_1999	AY633222	A/mallard/Alberta/215/99
NA(N8)	30~H7N8~ALB_1979	CY005040	A/pintail duck/Alberta/121/1979
NA(N8)	31~H3N8~ALB_1980	CY005045	A/pintail duck/Alberta/169/1980
NA(N8)	32~H3N8~OH_2002	CY011030	A/blue-winged teal/Ohio/926/2002
NA(N8)	33~H3N8~ALB_1991	CY014573	A/blue-winged teal/Alberta/120/1991
NA(N8)	34~H4N8~NY_1982	CY014931	A/mallard duck/New York/194/1982
NA(N8)	35~H6N8~NY_1982	CY014947	A/wood duck/New York/60/1982
NA(N8)	36~H6N8~NY_1982	CY014955	A/mallard duck/New York/90/1982
NA(N8)	37~H3N8~NY_1982	CY014963	A/mallard duck/New York/174/1982
NA(N8)	38~H6N8~DE_1998	CY015129	A/ruddy turnstone/Delaware/105/1998
NA(N8)	39~H3N8~AK_2005	CY015494	A/mallard/Alaska/256/2005
NA(N8)	4~H6N8~ALB_1994	AY633238	A/mallard/Alberta/232/94
NA(N8)	40~H3N8~AK_2005	CY015502	A/pintail/Alaska/279/2005
NA(N8)	41~H3N8~OH_1987	CY016142	A/pintail/Ohio/454/1987
NA(N8)	42~H6N8~OH_1989	CY016174	A/green-winged teal/Ohio/59/1989
NA(N8)	43~H3N8~AK_2005	CY016182	A/pintail/Alaska/211/2005
NA(N8)	44~H3N8~OH_1986	CY016397	A/mallard/Ohio/264/1986
NA(N8)	45~H3N8~AK_2005	CY016413	A/mallard/Alaska/715/2005
NA(N8)	46~H6N8~OH_1989	CY016621	A/mallard/Ohio/64/1989
NA(N8)	47~H3N8~AK_2005	CY017759	A/pintail/Alaska/779/2005
NA(N8)	48~H3N8~MD_2005	CY017775	A/longtail duck/Maryland/291/2005
NA(N8)	49~H3N8~OH_1987	CY017839	A/green-winged teal/Ohio/466/1987
NA(N8)	5~H3N8~ALB_1998	AY633246	A/mallard/Alberta/242/98
NA(N8)	50~H3N8~OH_1987	CY017863	A/northern shoveler/Ohio/454/1987
NA(N8)	51~H3N8~OH_1987	CY019199	A/pintail/Ohio/339/1987
NA(N8)	52~H3N8~OH_2002	CY020743	A/mallard/Ohio/654/2002
NA(N8)	53~H3N8~OH_2002	CY020759	A/mallard/Ohio/649/2002
NA(N8)	54~H4N8~MD_2002	CY020767	A/black duck/Maryland/834/2002
NA(N8)	55~H6N8~OH_1998	CY020871	A/mallard/Ohio/217/1998
NA(N8)	56~H3N8~AK_2005	CY020879	A/pintail/Alaska/49/2005
NA(N8)	57~H10N8~MD_2005	CY020903	A/longtail duck/Maryland/295/2005
NA(N8)	58~H10N8~MD_2005	CY020911	A/common scoter/Maryland/297/2005
NA(N8)	59~H3N8~OH_1986	CY020935	A/northern shoveler/Ohio/35/1986
NA(N8)	6~H3N8~ALB_1998	AY633254	A/mallard/Alberta/279/98
NA(N8)	60~H6N8~OH_1998	CY020959	A/mallard/Ohio/242/1998
NA(N8)	61~H6N8~OH_1989	CY020975	A/mallard/Ohio/115/1989

NA(N8)	62~H6N8~OH_1993	CY021199	A/green-winged teal/Ohio/80/1993
NA(N8)	63~H6N8~OH_1989	CY021207	A/mallard/Ohio/123/1989
NA(N8)	64~H3N8~OH_2005	CY021319	A/green-winged teal/Ohio/1289/2005
NA(N8)	65~H3N8~OH_2005	CY021343	A/mallard/Ohio/1801/2005
NA(N8)	66~H6N8~MD_2005	CY021479	A/mallard/Maryland/2022/2005
NA(N8)	67~H7N8~OH_1987	CY021623	A/mallard/Ohio/421/1987
NA(N8)	68~H6N8~OH_1989	CY021679	A/Canada goose/Ohio/127/1989
NA(N8)	69~H6N8~MD_2005	CY024796	A/mallard/Maryland/1983/2005
NA(N8)	7~H6N8~ALB_1994	AY633302	A/mallard/Alberta/76/94
NA(N8)	70~H3N8~OH_2006	CY029931	A/blue-winged teal/Ohio/1864/2006
NA(N8)	71~H3N8~CA_2007	CY032722	A/northern pintail/California/HKWF792/2007
NA(N8)	72~H4N8~CA_2007	CY032894	A/bufflehead/California/HKWF205/2007
NA(N8)	73~H4N8~AK_2007	CY035785	A/least sandpiper/South Central Alaska/2/2007
NA(N8)	74~H4N8~AK_2007	CY035793	A/least sandpiper/South Central Alaska/3/2007
NA(N8)	75~H3N8~AK_2007	CY035801	A/mallard/Interior Alaska/2/2007
NA(N8)	76~H3N8~AK_2007	CY035809	A/American widgeon/Interior Alaska/1/2007
NA(N8)	77~H3N8~AK_2007	CY035817	A/northern shoveler/Interior Alaska/1/2007
NA(N8)	78~H4N8~AK_2007	CY035871	A/least sandpiper/South Central Alaska/1/2007
NA(N8)	79~H3N8~AK_2007	CY035879	A/mallard/Interior Alaska/1/2007
NA(N8)	8~H6N8~ALB_1994	AY633326	A/pintail/Alberta/155/94
NA(N8)	80~H3N8~AK_2007	CY036661	A/green-winged teal/Interior Alaska/1/2007
NA(N8)	81~H3N8~MAN_2005	CY037009	A/blue-winged teal/Manitoba/13436/2005
NA(N8)	82~H3N8~NJ_2008	CY038172	A/ruddy turnstone/New Jersey/Sg-00525/2008
NA(N8)	83~H3N8~AK_2007	CY038364	A/mallard/Interior Alaska/3/2007
NA(N8)	84~H4N8~AK_2007	CY038372	A/northern pintail/Interior Alaska/2/2007
NA(N8)	85~H3N8~AK_2007	CY038380	A/mallard/Interior Alaska/4/2007
NA(N8)	86~H3N8~AK_2007	CY038388	A/American green-winged teal/Interior Alaska/3/2007
NA(N8)	87~H3N8~AK_2007	CY039757	A/mallard/Interior Alaska/6/2007
NA(N8)	88~H3N8~AK_2007	CY039765	A/northern shoveler/Interior Alaska/2/2007
NA(N8)	89~H3N8~AK_2007	CY039780	A/northern pintail/Interior Alaska/1/2007
NA(N8)	9~H3N8~ALB_1997	AY633342	A/pintail/Alberta/156/97
NA(N8)	90~H3N8~AK_2007	CY039796	A/northern shoveler/Interior Alaska/3/2007
NA(N8)	91~H3N8~AK_2007	CY039804	A/American green-winged teal/Interior Alaska/4/2007
NA(N8)	92~H3N8~AK_2007	CY039820	A/mallard/Interior Alaska/5/2007
NA(N8)	93~H3N8~AK_2007	CY039828	A/northern shoveler/Interior Alaska/4/2007
NA(N8)	94~H3N8~AK_2007	CY039852	A/mallard/Interior Alaska/7/2007
NA(N8)	95~H3N8~AK_2007	CY039875	A/mallard/Interior Alaska/8/2007
NA(N8)	96~H6N8~DE_2006	CY041284	A/shorebird/Delaware/133/2006
NA(N8)	97~H3N8~MN_2007	CY041997	A/blue-winged teal/Minnesota/Sg-00037/2007
NA(N8)	98~H3N8~MN_2007	CY042004	A/gadwall duck/Minnesota/Sg-00044/2007
NA(N8)	99~H3N8~MN_2007	CY042007	A/mallard duck/Minnesota/Sg-00047/2007
M1	1~H13N6_1980	CY005859	A/gull/Minnesota/945/1980
M1	100~H9N2_1998	DQ021715	A/mallard/MN/281/98
M1	101~H3N8_1999	DQ021716	A/mallard/Minnesota/283/1999
M1	102~H7N3_1998	DQ021717	A/mallard/MN/284/98
M1	103~H9N2_1998	DQ021718	A/mallard/MN/304/98
M1	104~H6N8_1998	DQ021719	A/mallard/Minnesota/31/1998
M1	105~H6N5_1999	DQ021720	A/mallard/Minnesota/334/1999
M1	106~H3N4_1999	DQ021721	A/mallard/MN/351/99
M1	107~H3N8_1999	DQ021722	A/mallard/Minnesota/354/1999
M1	108~H9N2_1999	DQ021723	A/mallard/MN/365/99
M1	109~H11N9_1999	DQ021724	A/mallard/Minnesota/403/99
M1	11~H4N6_2007	CY048670	A/mallard/Minnesota/Sg-00176/2007
M1	110~H2N3_1998	DQ021725	A/mallard/MN/51/1998
M1	111~H3N2_1999	DQ021726	A/mallard/Minnesota/68/1999
M1	112~H11N2_1998	DQ021727	A/mallard/MN/7/98
M1	113~H7N3_1998	DQ021728	A/mallard/MN/88/98
M1	114~H6N8_1999	DQ021731	A/pintail/Minnesota/410/1999
M1	115~H7N3_1999	DQ021732	A/pintail/Minnesota/423/1999
M1	116~H6N2_1999	DQ021733	A/pintail/Minnesota/431/1999
M1	117~H3N6_1999	DQ021734	A/pintail/Minnesota/479/1999
M1	118~H6N3_2000	DQ021751	A/mallard/MN/346233/00
M1	119~H4N6_1998	DQ021770	A/mallard/Minnesota/220/1998
M1	12~H12N9_2007	CY048671	A/mallard/Minnesota/Sg-00177/2007
M1	120~H4N2_1998	DQ021771	A/mallard/Minnesota/371/1998
M1	121~H4N6_2000	DQ021773	A/mallard/Minnesota/348/2000
M1	122~H4N8_1999	DQ021779	A/mallard/MN/347/99
M1	123~H4N8_1999	DQ021780	A/mallard/MN/352/99
M1	124~H11N9_2000	EU743458	A/mallard/Minnesota/42/2000
M1	125~H11N9_2000	EU743466	A/mallard/Minnesota/107/2000
M1	126~H11N9_2000	EU743473	A/mallard/Minnesota/109/2000
M1	127~H3N2_1999	EU743502	A/mallard/Minnesota/290/1999
M1	128~H3N2_1999	EU743509	A/mallard/Minnesota/380/1999
M1	129~H3N1_1999	EU743517	A/mallard/MN/330/1999

M1	130~H3N6_1999	EU743537	A/mallard/MN/259/1999
M1	131~H3N6_2000	EU743545	A/mallard/MN/99/2000
M1	132~H3N6_2000	EU743553	A/mallard/MN/515/2000
M1	133~H3N6_1999	EU743561	A/pintail/Minnesota/479/1999
M1	134~H3N9_2000	EU871869	A/mallard/MN/158/2000
M1	135~H4N6_1999	EU871877	A/mallard/Minnesota/160/1999
M1	136~H4N6_1999	EU871885	A/mallard/Minnesota/269/1999
M1	137~H4N8_1999	EU871893	A/mallard/MN/323/1999
M1	138~H5N2_2000	EU871900	A/mallard/MN/113/2000
M1	139~H5N3_2000	EU871907	A/mallard/MN/479/2000
M1	14~H11N9_2007	CY048673	A/mallard/Minnesota/Sg-00179/2007
M1	140~H5N5_2000	EU871914	A/mallard/MN/105/2000
M1	141~H5N2_2000	FJ357070	A/mallard/MN/1/2000
M1	142~H2N3_1998	FJ357078	A/mallard/MN/14/1998
M1	143~H2N3_2000	FJ517268	A/mallard/MN/506/2000
M1	144~H3N8_1999	FJ517283	A/mallard/Minnesota/190/1999
M1	145~H4N8_1999	FJ517315	A/mallard/MN/327/1999
M1	146~H11N9_2000	GQ257374	A/mallard/Minnesota/249/2000
M1	147~H5N2_2006	GQ923214	A/mallard/Minnesota/464334/2006
M1	148~H2N2_1998	GU050725	A/mallard/Minnesota/26/1998
M1	149~H2N2_1998	GU050731	A/mallard/Minnesota/27/1998
M1	150~H2N1_2000	GU050744	A/mallard/Minnesota/550/2000
M1	151~H2N9_1998	GU050800	A/mallard/Minnesota/34/1998
M1	152~H2N9_2000	GU050807	A/mallard/Minnesota/31/2000
M1	153~H10N7_2000	GU050883	A/mallard/Minnesota/518/2000
M1	154~H3N1_2000	GU051129	A/mallard/Minnesota/417/2000
M1	155~H3N2_2000	GU051141	A/mallard/Minnesota/282/2000
M1	156~H3N8_2000	GU051148	A/mallard/Minnesota/448/2000
M1	157~H4N2_1998	GU051206	A/mallard/Minnesota/95/1998
M1	158~H4N3_2000	GU051212	A/mallard/Minnesota/14/2000
M1	159~H4N6_1999	GU051226	A/mallard/Minnesota/140/1999
M1	16~H3N8_2007	CY048676	A/mallard/Minnesota/Sg-00184/2007
M1	160~H4N6_1999	GU051232	A/mallard/Minnesota/193/1999
M1	161~H4N6_2000	GU051240	A/mallard/Minnesota/313/2000
M1	162~H4N6_2000	GU051246	A/mallard/Minnesota/524/2000
M1	163~H4N8_1998	GU051252	A/mallard/Minnesota/114/1998
M1	164~H4N8_1999	GU051258	A/mallard/Minnesota/168/1999
M1	165~H4N8_1999	GU051262	A/mallard/Minnesota/188/1999
M1	166~H4N8_1999	GU051267	A/mallard/Minnesota/210/1999
M1	167~H4N8_1999	GU051275	A/mallard/Minnesota/237/1999
M1	168~H4N8_1999	GU051282	A/mallard/Minnesota/271/1999
M1	169~H4N8_1999	GU051288	A/mallard/Minnesota/402/1999
M1	17~H3N6_2007	CY048677	A/mallard/Minnesota/Sg-00185/2007
M1	170~H5N1_2000	GU051302	A/mallard/Minnesota/24/2000
M1	171~H5N2_2000	GU051309	A/mallard/Minnesota/166/2000
M1	172~H5N2_2000	GU051314	A/mallard/Minnesota/168/2000
M1	173~H5N2_2000	GU051324	A/mallard/Minnesota/410/2000
M1	174~H5N3_2000	GU051333	A/mallard/Minnesota/382/2000
M1	175~H6N4_1999	GU051383	A/mallard/Minnesota/313/1999
M1	176~H6N5_1998	GU051417	A/mallard/Minnesota/66/1998
M1	177~H6N5_1999	GU051426	A/mallard/Minnesota/329/1999
M1	178~H6N6_1998	GU051434	A/mallard/Minnesota/179/1998
M1	179~H6N8_1998	GU051439	A/mallard/Minnesota/29/1998
M1	180~H6N8_1998	GU051444	A/mallard/Minnesota/63/1998
M1	181~H6N8_1999	GU051453	A/mallard/Minnesota/173/1999
M1	182~H6N8_1999	GU051459	A/mallard/Minnesota/204/1999
M1	183~H9N2_1998	GU051515	A/mallard/Minnesota/38/1998
M1	184~H9N2_1998	GU051517	A/mallard/Minnesota/59/1998
M1	185~H9N2_1998	GU051519	A/mallard/Minnesota/153/1998
M1	186~H9N2_1998	GU051520	A/mallard/Minnesota/231/1998
M1	187~H9N2_1998	GU051523	A/mallard/Minnesota/250/1998
M1	188~H9N2_1998	GU051526	A/mallard/Minnesota/309/1998
M1	189~H9N2_1999	GU051528	A/mallard/Minnesota/166/1999
M1	190~H9N2_1999	GU051532	A/mallard/Minnesota/341/1999
M1	2~H9N2_1980	CY005874	A/goose/MN/5733-1/1980
M1	20~H3N8_2007	CY048682	A/mallard/Minnesota/Sg-00190/2007
M1	22~H10N3_2007	CY048684	A/mallard/Minnesota/Sg-00194/2007
M1	26~H4N6_2007	CY048689	A/mallard/Minnesota/Sg-00210/2007
M1	27~H11N9_2007	CY048692	A/green-winged teal/Minnesota/Sg-00213/2007
M1	3~H6N6_1980	CY005882	A/blue-winged teal/MN/993/1980
M1	30~H10N7_2007	CY048695	A/mallard/Minnesota/Sg-00221/2007
M1	31~H6N2_2007	CY048696	A/green-winged teal/Minnesota/Sg-00222/2007
M1	32~H4N8_2008	CY048810	A/mallard/Minnesota/Sg-00625/2008
M1	33~H4N8_2008	CY048811	A/mallard/Minnesota/Sg-00626/2008

M1	34~H1N1_2008	CY048813	A/mallard/Minnesota/Sg-00627/2008
M1	37~H10N7_2008	CY048819	A/mallard/Minnesota/Sg-00632/2008
M1	38~H3N8_2008	CY048821	A/mallard/Minnesota/Sg-00634/2008
M1	39~H4N8_2008	CY048822	A/mallard/Minnesota/Sg-00635/2008
M1	4~H10N7_1979	CY014740	A/mallard duck/Minnesota/19/1979
M1	40~H3N8_2008	CY048823	A/mallard/Minnesota/Sg-00637/2008
M1	41~H3N8_2008	CY048824	A/mallard/Minnesota/Sg-00638/2008
M1	43~H3N8_2008	CY048827	A/mallard/Minnesota/Sg-00640/2008
M1	44~H3N8_2008	CY048829	A/mallard/Minnesota/Sg-00641/2008
M1	45~H3N8_2008	CY048832	A/mallard/Minnesota/Sg-00643/2008
M1	46~H3N8_2008	CY048834	A/mallard/Minnesota/Sg-00644/2008
M1	47~H3N8_2008	CY048836	A/northern shoveler/Minnesota/Sg-00645/2008
M1	48~H4N4_2008	CY048838	A/northern shoveler/Minnesota/Sg-00646/2008
M1	5~H3N2_2007	CY041890	A/green-winged teal/Minnesota/Sg-00131/2007
M1	50~H8N4_2008	CY048842	A/northern shoveler/Minnesota/Sg-00648/2008
M1	51~H3N8_2008	CY048846	A/blue-winged teal/Minnesota/Sg-00650/2008
M1	52~H1N1_2008	CY048848	A/northern shoveler/Minnesota/Sg-00651/2008
M1	53~H3N8_2008	CY048850	A/northern shoveler/Minnesota/Sg-00652/2008
M1	55~H3N8_2008	CY048853	A/northern shoveler/Minnesota/Sg-00654/2008
M1	59~H3N8_2008	CY048862	A/northern shoveler/Minnesota/Sg-00659/2008
M1	6~H4N6_2007	CY041898	A/mallard/Minnesota/Sg-00133/2007
M1	60~H3N8_2008	CY048864	A/northern shoveler/Minnesota/Sg-00660/2008
M1	62~H3N8_2008	CY048867	A/northern shoveler/Minnesota/Sg-00662/2008
M1	64~H3N8_2008	CY048871	A/northern shoveler/Minnesota/Sg-00664/2008
M1	65~H3N8_2008	CY048875	A/northern shoveler/Minnesota/Sg-00667/2008
M1	67~H3N8_2008	CY048879	A/northern shoveler/Minnesota/Sg-00669/2008
M1	68~H3N8_2008	CY048880	A/northern shoveler/Minnesota/Sg-00670/2008
M1	69~H3N8_2008	CY048882	A/northern shoveler/Minnesota/Sg-00671/2008
M1	7~H1N3_2007	CY048655	A/mallard/Minnesota/Sg-00162/2007
M1	70~H3N8_2008	CY048884	A/mallard/Minnesota/Sg-00672/2008
M1	71~H8N4_2008	CY048886	A/mallard/Minnesota/Sg-00673/2008
M1	72~H3N8_2008	CY048888	A/mallard/Minnesota/Sg-00674/2008
M1	73~H8N4_2008	CY048890	A/mallard/Minnesota/Sg-00675/2008
M1	74~H3N8_2008	CY048892	A/mallard/Minnesota/Sg-00676/2008
M1	75~H3N8_2008	CY048894	A/mallard/Minnesota/Sg-00677/2008
M1	76~H6N8_1998	DQ021691	A/mallard/MN/107/98
M1	77~H5N2_1998	DQ021692	A/mallard/MN/133/1998
M1	78~H4N6_1999	DQ021693	A/mallard/Minnesota/145/1999
M1	80~H4N8_1999	DQ021695	A/mallard/MN/154/99
M1	81~H6N5_1998	DQ021696	A/mallard/Minnesota/157/1998
M1	82~H7N7_1999	DQ021697	A/mallard/Minnesota/17/1999
M1	83~H6N8_1999	DQ021698	A/mallard/Minnesota/175/1999
M1	84~H3N9_1998	DQ021699	A/mallard/Minnesota/182/1998
M1	85~H9N2_1999	DQ021700	A/mallard/Minnesota/186/1999
M1	86~H7N3_1998	DQ021701	A/mallard/MN/187/98
M1	87~H7N3_1998	DQ021702	A/mallard/MN/190/98
M1	88~H4N6_1999	DQ021703	A/mallard/Minnesota/192/1999
M1	89~H4N6_1999	DQ021704	A/mallard/Minnesota/195/1999
M1	9~H6N1_2007	CY048661	A/mallard/Minnesota/Sg-00167/2007
M1	90~H4N6_1999	DQ021705	A/mallard/Minnesota/198/1999
M1	91~H4N6_1998	DQ021706	A/mallard/Minnesota/2/1998
M1	92~H4N8_1999	DQ021707	A/mallard/Minnesota/212/1999
M1	93~H3N8_1999	DQ021708	A/mallard/Minnesota/218/1999
M1	94~H3N8_1999	DQ021709	A/mallard/Minnesota/225/1999
M1	95~H3N8_1999	DQ021710	A/mallard/Minnesota/231/1999
M1	96~H9N2_1998	DQ021711	A/mallard/MN/232/98
M1	97~H6N5_1999	DQ021712	A/mallard/Minnesota/253/1999
M1	98~H4N9_1999	DQ021713	A/mallard/Minnesota/263/1999
M1	99~H3N5_1999	DQ021714	A/mallard/Minnesota/280/1999
NS	1~H13N6_1980	CY005862	A/gull/Minnesota/945/1980
NS	10~H9N2_1998	DQ021558	A/mallard/Minnesota/153/1998
NS	100~H4N8_1999	GU051290	A/mallard/Minnesota/402/1999
NS	101~H5N1_2000	GU051305	A/mallard/Minnesota/24/2000
NS	102~H5N2_2000	GU051311	A/mallard/Minnesota/166/2000
NS	103~H5N2_2000	GU051316	A/mallard/Minnesota/168/2000
NS	104~H5N2_2000	GU051320	A/mallard/Minnesota/283/2000
NS	105~H5N2_2000	GU051326	A/mallard/Minnesota/410/2000
NS	106~H5N3_2000	GU051335	A/mallard/Minnesota/382/2000
NS	107~H6N4_1999	GU051384	A/mallard/Minnesota/313/1999
NS	108~H6N5_1998	GU051419	A/mallard/Minnesota/66/1998
NS	109~H6N5_1999	GU051428	A/mallard/Minnesota/329/1999
NS	11~H7N7_1999	DQ021559	A/mallard/Minnesota/17/1999
NS	110~H6N6_1998	GU051436	A/mallard/Minnesota/179/1998
NS	111~H6N8_1998	GU051441	A/mallard/Minnesota/29/1998

NS	112~H6N8_1998	GU051446	A/mallard/Minnesota/63/1998
NS	113~H6N8_1998	GU051450	A/mallard/Minnesota/353/1998
NS	114~H6N8_1999	GU051455	A/mallard/Minnesota/173/1999
NS	115~H6N8_1999	GU051461	A/mallard/Minnesota/204/1999
NS	116~H7N3_1999	GU051497	A/pintail/Minnesota/423/1999
NS	117~H9N2_1998	GU051516	A/mallard/Minnesota/38/1998
NS	118~H9N2_1998	GU051518	A/mallard/Minnesota/59/1998
NS	119~H9N2_1998	GU051521	A/mallard/Minnesota/231/1998
NS	12~H4N6_1998	DQ021560	A/mallard/Minnesota/2/1998
NS	120~H9N2_1998	GU051524	A/mallard/Minnesota/250/1998
NS	121~H9N2_1999	GU051529	A/mallard/Minnesota/166/1999
NS	122~H9N2_1999	GU051533	A/mallard/Minnesota/341/1999
NS	123~H13N6_1981	M80958	A/gull/Minnesota/1352/1981
NS	13~H4N6_1998	DQ021561	A/mallard/Minnesota/220/1998
NS	14~H6N5_1999	DQ021562	A/mallard/Minnesota/253/1999
NS	15~H2N2_1998	DQ021563	A/mallard/Minnesota/26/1998
NS	16~H3N5_1999	DQ021564	A/mallard/Minnesota/280/1999
NS	17~H3N8_1999	DQ021565	A/mallard/Minnesota/281/1999
NS	18~H9N2_1998	DQ021566	A/mallard/Minnesota/309/1998
NS	19~H6N8_1998	DQ021567	A/mallard/Minnesota/31/1998
NS	2~H9N2_1980	CY005877	A/goose/MN/5733-1/1980
NS	20~H6N8_1998	DQ021568	A/mallard/Minnesota/327/1998
NS	21~H4N2_1998	DQ021570	A/mallard/Minnesota/371/1998
NS	22~H2N3_1998	DQ021571	A/mallard/MN/51/1998
NS	23~H4N6_2000	DQ021572	A/mallard/Minnesota/530/2000
NS	24~H11N2_1998	DQ021573	A/mallard/MN/7/98
NS	25~H6N8_1999	DQ021580	A/pintail/Minnesota/410/1999
NS	26~H6N8_1998	DQ021591	A/mallard/MN/154/98
NS	27~H6N5_1998	DQ021592	A/mallard/Minnesota/157/1998
NS	28~H6N8_1999	DQ021593	A/mallard/Minnesota/175/1999
NS	29~H3N9_1998	DQ021594	A/mallard/Minnesota/182/1998
NS	3~H6N6_1980	CY005885	A/blue-winged teal/MN/993/1980
NS	30~H9N2_1999	DQ021595	A/mallard/Minnesota/186/1999
NS	31~H7N3_1998	DQ021596	A/mallard/MN/187/98
NS	32~H4N6_1999	DQ021597	A/mallard/Minnesota/192/1999
NS	33~H4N6_1999	DQ021598	A/mallard/Minnesota/195/1999
NS	34~H4N6_1999	DQ021599	A/mallard/Minnesota/198/1999
NS	35~H9N2_1998	DQ021600	A/mallard/MN/232/98
NS	36~H2N2_1998	DQ021601	A/mallard/Minnesota/27/1998
NS	37~H9N2_1998	DQ021602	A/mallard/MN/281/98
NS	38~H7N3_1998	DQ021603	A/mallard/MN/284/98
NS	39~H9N2_1998	DQ021604	A/mallard/MN/304/98
NS	4~H10N7_1979	CY014743	A/mallard duck/Minnesota/19/1979
NS	40~H6N5_1999	DQ021605	A/mallard/Minnesota/334/1999
NS	41~H9N2_1999	DQ021606	A/mallard/MN/365/99
NS	42~H7N3_1998	DQ021607	A/mallard/MN/88/98
NS	43~H3N6_1999	DQ021608	A/pintail/Minnesota/479/1999
NS	44~H3N8_1999	DQ021623	A/mallard/Minnesota/225/1999
NS	45~H3N2_1999	DQ021624	A/mallard/Minnesota/290/1999
NS	46~H6N3_2000	DQ021626	A/mallard/MN/346233/00
NS	47~H4N8_1999	DQ021627	A/mallard/MN/347/99
NS	48~H4N6_2000	DQ021628	A/mallard/Minnesota/348/2000
NS	49~H4N8_1999	DQ021629	A/mallard/MN/352/99
NS	5~H3N2_2007	CY041891	A/green-winged teal/Minnesota/Sg-00131/2007
NS	50~H3N8_1999	DQ021630	A/mallard/Minnesota/354/1999
NS	51~H11N9_1999	DQ021631	A/mallard/Minnesota/403/99
NS	52~H6N2_1999	DQ021637	A/pintail/Minnesota/431/1999
NS	53~H3N8_1999	DQ021642	A/mallard/Minnesota/190/1999
NS	54~H4N8_1999	DQ021643	A/mallard/Minnesota/212/1999
NS	55~H3N8_1999	DQ021644	A/mallard/Minnesota/218/1999
NS	56~H4N9_1999	DQ021645	A/mallard/Minnesota/263/1999
NS	57~H3N4_1999	DQ021646	A/mallard/MN/351/99
NS	58~H3N2_1999	DQ021647	A/mallard/Minnesota/68/1999
NS	59~H11N9_2000	EU743461	A/mallard/Minnesota/42/2000
NS	6~H4N6_2007	CY041899	A/mallard/Minnesota/Sg-00133/2007
NS	60~H11N9_2000	EU743469	A/mallard/Minnesota/107/2000
NS	61~H11N9_2000	EU743476	A/mallard/Minnesota/109/2000
NS	62~H3N2_1999	EU743512	A/mallard/Minnesota/380/1999
NS	63~H3N1_1999	EU743520	A/mallard/MN/330/1999
NS	64~H3N6_1999	EU743540	A/mallard/MN/259/1999
NS	65~H3N6_2000	EU743548	A/mallard/MN/99/2000
NS	66~H3N6_2000	EU743556	A/mallard/MN/515/2000
NS	67~H3N6_1999	EU743564	A/pintail/Minnesota/479/1999
NS	68~H3N9_2000	EU871872	A/mallard/MN/158/2000

NS	69~H4N6_1999	EU871880	A/mallard/Minnesota/160/1999
NS	7~H6N8_1998	DQ021555	A/mallard/MN/107/98
NS	70~H4N6_1999	EU871888	A/mallard/Minnesota/269/1999
NS	71~H4N8_1999	EU871896	A/mallard/MN/323/1999
NS	72~H5N2_2000	EU871903	A/mallard/MN/113/2000
NS	73~H5N3_2000	EU871910	A/mallard/MN/479/2000
NS	74~H5N5_2000	EU871917	A/mallard/MN/105/2000
NS	75~H5N2_2000	FJ357073	A/mallard/MN/1/2000
NS	76~H2N3_1998	FJ357081	A/mallard/MN/14/1998
NS	77~H2N3_2000	FJ517271	A/mallard/MN/506/2000
NS	78~H3N8_1999	FJ517292	A/mallard/Minnesota/283/1999
NS	79~H4N8_1999	FJ517318	A/mallard/MN/327/1999
NS	8~H5N2_1998	DQ021556	A/mallard/MN/133/1998
NS	80~H11N9_2000	GQ257377	A/mallard/Minnesota/249/2000
NS	81~H5N2_2006	GQ923217	A/mallard/Minnesota/464334/2006
NS	82~H2N1_2000	GU050747	A/mallard/Minnesota/550/2000
NS	83~H2N9_1998	GU050803	A/mallard/Minnesota/34/1998
NS	84~H2N9_2000	GU050810	A/mallard/Minnesota/31/2000
NS	85~H10N7_2000	GU050886	A/mallard/Minnesota/518/2000
NS	86~H3N1_2000	GU051131	A/mallard/Minnesota/417/2000
NS	87~H3N2_2000	GU051143	A/mallard/Minnesota/282/2000
NS	88~H3N8_2000	GU051151	A/mallard/Minnesota/448/2000
NS	89~H4N2_1998	GU051208	A/mallard/Minnesota/95/1998
NS	9~H4N6_1999	DQ021557	A/mallard/Minnesota/145/1999
NS	90~H4N3_2000	GU051214	A/mallard/Minnesota/14/2000
NS	91~H4N6_1999	GU051234	A/mallard/Minnesota/193/1999
NS	92~H4N6_2000	GU051243	A/mallard/Minnesota/313/2000
NS	93~H4N6_2000	GU051249	A/mallard/Minnesota/524/2000
NS	94~H4N8_1998	GU051254	A/mallard/Minnesota/114/1998
NS	95~H4N8_1999	GU051260	A/mallard/Minnesota/168/1999
NS	96~H4N8_1999	GU051264	A/mallard/Minnesota/188/1999
NS	97~H4N8_1999	GU051269	A/mallard/Minnesota/210/1999
NS	98~H4N8_1999	GU051277	A/mallard/Minnesota/237/1999
NS	99~H4N8_1999	GU051284	A/mallard/Minnesota/271/1999
